# Unusual substrate and halide versatility of phenolic halogenase PltM

**DOI:** 10.1038/s41467-019-09215-9

**Published:** 2019-03-19

**Authors:** Shogo Mori, Allan H. Pang, Nishad Thamban Chandrika, Sylvie Garneau-Tsodikova, Oleg V. Tsodikov

**Affiliations:** Department of Pharmaceutical Sciences, College of Pharmacy, University of Kentucky, Lexington, KY 40536-0596 USA

**Keywords:** Enzymes, X-ray crystallography

## Abstract

Controlled halogenation of chemically versatile substrates is difficult to achieve. Here we describe a unique flavin-dependent halogenase, PltM, which is capable of utilizing a wide range of halides for installation on a diverse array of phenolic compounds, including FDA-approved drugs and natural products, such as terbutaline, fenoterol, resveratrol, and catechin. Crystal structures of PltM in complex with phloroglucinol and FAD in different states yield insight into substrate recognition and the FAD recycling mechanism of this halogenase.

## Abstract (web summary)

Halogenase enzymes are of interest as halogenating tools for organic synthesis. Here the authors show that the bacterial FAD-dependent phenolic halogenase PltM chlorinates, brominates and iodinates a variety of substrates and reveal the structural basis for its substrate versatility and provide insights into the FAD recycling mechanism of PltM.

## Introduction

Halogenation is an important chemical modification with a potential to increase biological activity and bioavailability of molecules^1,2^. Moreover, halogen groups can be further synthetically elaborated by transition metal-catalyzed coupling reactions^3–5^. Halogenase enzymes are attractive potential halogenating tools, because, unlike synthetic halogenation, these enzymes ensure both regiospecificity and green chemistry.

Flavin adenine dinucleotide (FAD)-dependent tryptophan (Trp) halogenases have been the focus of development as halogenation tools^6^. Mutagenesis of Trp halogenase RebH increased its stability, catalytic efficiency^7^, and substrate scope, to halogenate natural products and drug-like molecules^8^. Furthermore, halogenation on a gram scale by this enzyme was achieved by cross-linking it to coupled enzymes^9^. A recent study of the detailed substrate profile of several bacterial Trp halogenases (including RebH) and two fungal phenolic halogenases (Rdc2 and GsfI) indicated that Trp halogenases displayed preference towards indole, phenylpiperidine, phenylpyrrole, and phenoxyaniline derivatives as substrates, while phenolic halogenases had a narrow substrate profile of some anilines, phenol derivatives^10^, and natural products such as macrolactones and curcumin^11,12^. While the substrate profiles of some FAD-dependent Trp halogenases appear to be quite broad^10^, the halide spectrum of characterized Trp and phenolic halogenases has been limited to at most two halides: most commonly chloride (Cl^−^) and bromide (Br^−^) ions, and for a phenolic halogenase Bmp5, bromide (Br^−^) and iodide (I^−^)^13^.

In these enzymes, the enzyme–FAD complex catalyzes conversion of a halide ion into a highly reactive hypohalous acid HOX, which diffuses through a protein channel protected from solvent to the substrate binding site, where it is proposed to react with a catalytic lysine residue to form a haloamine adduct^14–16^, or to form hydrogen bonds with catalytic lysine and glutamic acid residues to act as an active oxidant^17^, with subsequent halogenation of the substrate. FAD is usually a prosthetic group that is tightly and, in some cases, covalently bound to the enzyme, co-purifying with it^18^. Some FAD-dependent halogenases use FAD that can dissociate from the enzyme for reduction (Supplementary Table 1)^14,19,20^. How FAD can dissociate and rebind into the confines of its binding site remains unclear.

**Fig. 1:**
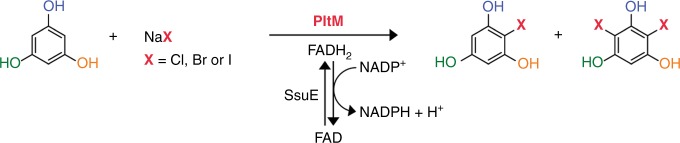
Halogenation by PltM. Schematic representation of phloroglucinol (**1**) halogenation by PltM

A bacterial halogenase PltM was originally identified as one of three putative halogenases encoded in the biosynthetic gene cluster of pyoluteorin, an antifungal compound containing a dichloropyrrole moiety^21^. Recently, PltM was elegantly demonstrated to catalyze mono- and dichlorination of phloroglucinol (**1**) (Fig. 1), yielding, instead of a biosynthetic intermediate, a compound that serves as a potent transcriptional regulator of the pyolyteorin biosynthesis^22^, whereas another halogenase, PltA, which acted on a peptidyl carrier protein loaded pyrrole, was shown to generate dichloropyrrole^23,24^. Herein, we report the wide substrate profile and the unusually large halide versatility of PltM and its crystal structures that reveal a unique recycling mechanism of FAD.

## Results

### Halide versatility of PltM

**Fig. 2:**
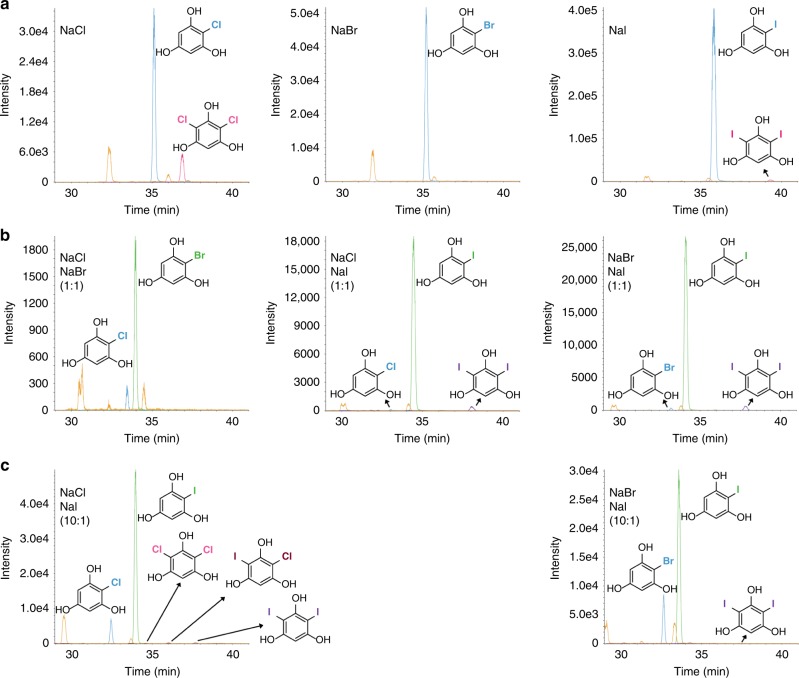
Halogenation of phloroglucinol (**1**) by PltM. **a** XIC traces showing the homo-halogenation of **1** by PltM with NaCl (left), NaBr (middle), and NaI (right) as halide sources. Blue and pink traces depict mono-halogenation and dihalogenation, respectively. **b** Halogenation of **1** by PltM with equimolar ratio of NaCl/NaBr (left), NaCl/NaI (middle), and NaBr/NaI (right). Blue and green traces show mono-halogenation with smaller and larger halogens, respectively; while purple trace indicates dehalogenation. **c** Halogenation of **1** by PltM with NaCl/NaI (left) and NaBr/NaI (right) in a 10:1 ratio. Blue and green traces show mono-halogenation with smaller and larger halogens, respectively; while purple and pink traces indicate homo-dihalogenation with smaller and larger halogens, respectively. The brown trace displays hetero-dihalogenated products. The orange trace shows the unreacted substrate **1**

To explore the halide profile of PltM, we first tested halogenation of **1** by PltM with NaF, NaCl, NaBr, and NaI used individually in a reaction mixture. We identified chlorinated, brominated, and iodinated, but not fluorinated **1** as products (Fig. 2a, Supplementary Fig. 1, Supplementary Table 2). To our knowledge, PltM is the only example of an FAD-dependent halogenase that is able to use three different halides, Cl^−^, Br,^−^ and I^−^. We observed mono- and dihalogenation of **1** with chloride and iodide, but only mono-halogenation with bromide; trihalogenation was never observed. We then carried out competitive halogenation assays of **1**, where two different halides (Cl^−^/Br^−^, Cl^−^/I^−^, or Br^−^/I^−^) were present in the reaction at equimolar ratios (Fig. 2b, Supplementary Figs. 2–4, Supplementary Table 2). Each of these reactions yielded mono-halogenated products of either halogen and diiodinated **1** where NaI was used, whereas products halogenated by two different halides were not observed. In an attempt to obtain a hetero-dihalogenated product, we used compound **1** in the presence of a 10-fold molar excess of NaCl or NaBr over NaI (Fig. 2c, Supplementary Figs. 5, 6, Supplementary Table 2). For the NaCl/NaI mixture, we identified all possible mono- and dihalogenated products, including chloro-iodinated **1**. For NaBr/NaI, we identified mono-brominated, mono-iodinated, and diiodinated **1**, and no additional products were observed. In fact, no further halogenation of the mono-brominated species was observed in any reaction.

### Substrate profile of PltM

**Fig. 3:**
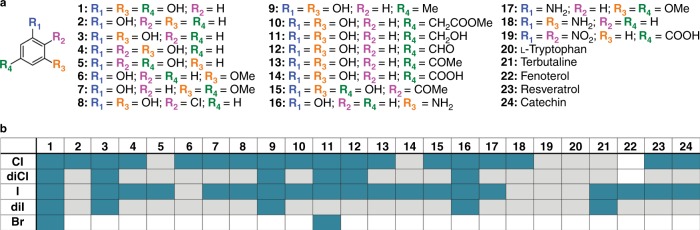
Substrate profile of PltM by LC-MS. **a** Compounds tested as potential substrates of PltM. **b** Summary of halogenation assay results. The top row and left column indicate the tested substrate and expected halogenation, respectively. Observed and unobserved halogenation are indicated by blue and gray boxes, respectively, while white boxes indicate untested halogenation

Having established the halide versatility of PltM, we next set out to investigate its substrate profile. We first tested a set of 20 structurally diverse small molecules, most, but not all of which were, like **1**, phenolic (phenolic derivatives, anilines, nitrobenzene derivative) and included l-Trp (Fig. 3a, Supplementary Fig. 7). All compounds were tested for chlorination and iodination (Fig. 3b, Supplementary Figs. 8–30, Supplementary Table 3). The products were detected and identified by liquid chromatography-mass spectrometry (LC-MS); chlorinated products were identified by the calculated mass and isotope ratio, and iodinated products were identified by the calculated mass, also using the corresponding chlorination reaction as a control. PltM catalyzed halogenation of 18 of the 20 compounds tested, exhibiting remarkable substrate versatility for phenolic compounds (Fig. 3b). The enzyme halogenated all phenolic (**1–16**) and aniline (**16–18**) derivatives tested, while it did not halogenate the nitrobenzene derivative **19**. These data suggest that the phenyl compounds with electron donating groups can be accepted by PltM as substrates even when one hydroxyl group is substituted with a moderate electron withdrawing group, such as aldehyde (**12**), ketone (**13**), and carboxylic acid (**14**). On the other hand, the strongly electron withdrawing nitro group is not tolerated. This correlation of the substrate electron withdrawing character with halogenation activity is consistent with other phenolic halogenases^10^. The halogenated l-Trp (**20**) was not observed, indicating that PltM is not a Trp halogenase, and that it is indeed a bona fide phenolic halogenase. As besides **1** the reaction with compound **11** showed very clear signals of chlorinated and iodinated **11**, we also tested compound **11** for bromination and fluorination (Supplementary Fig. 19, Supplementary Table 3). We observed mono-bromination of **11**, but not dibromination or fluorination, which is consistent with the halogenation profile on the natural substrate **1**. Encouraged by the wide substrate versatility of PltM as established with compounds **1–20**, we tested its halogenase activity on four larger molecules containing a phenolic derivative group. We tested the FDA-approved drugs terbutaline (**21**) and fenoterol (**22**), both short-acting β_2_ adrenoreceptor agonists that contain a resorcinol moiety in their structure (Supplementary Fig. 7). Iodinated terbutaline (both mono and di) and mono-iodinated fenoterol were obtained (Fig. 3b). We also tested the dietary natural products resveratrol (**23**) and catechin (**24**), which were both mono-chlorinated and mono-iodinated by PltM. These results demonstrate that PltM can be utilized for halogenation of larger drug-like molecules and natural products.

### Crystal structure of PltM and its complex with phloroglucinol

**Fig. 4:**
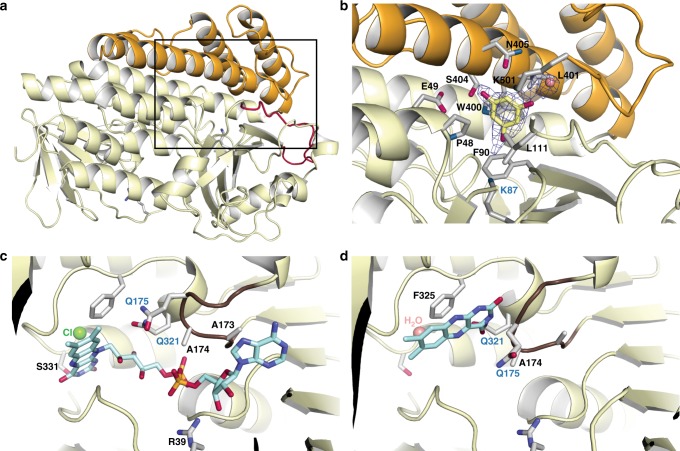
Crystal structures of PltM. **a** Full view of the structure of PltM with the conserved halogenase fold in pale yellow and the unique C-terminal region in orange. The red loop indicates the N-terminal unconserved region after the 3rd β-sheet. The substrate binding region is shown by a box. **b** A zoomed in view of the substrate binding site of the structure of PltM-compound **1** complex (yellow sticks). Residues lining the substrate binding pocket are shown as gray sticks and the *mF*_o_–*DF*_c_ polder omit map contoured at 5.5*σ* is shown by the gray mesh. **c** The FAD bound in the holoenzyme state of PltM. **d** The FAD bound in a putative FAD binding intermediate state. FAD is represented as turquoise sticks in panels **c** and **d**. The flexible loop that changes conformation upon FAD binding is shown in brown. Key FAD interacting residues are shown as sticks. Bound Cl^−^ and water are shown as green and salmon spheres, respectively

In addition to its remarkable halide versatility and a very broad substrate profile for a phenolic halogenase, PltM is at most ~15% identical in sequence to other structurally characterized FAD-dependent halogenases, and it contains a unique C-terminal region (residues 390–502) (Supplementary Fig. 31). These intriguing properties prompted us to obtain a 1.80 Å-resolution crystal structure of this enzyme (Supplementary Fig. 32, Supplementary Table 4). The crystal structure of PltM was obtained by the single anomalous dispersion (SAD) method by using ethylmercury derivatized crystals. PltM is a monomer in solution (Supplementary Fig. 32); the crystals of PltM contain four nearly structurally identical monomers per asymmetric unit. A monomer of PltM (Fig. 4a) consists of a large FAD binding fold that is conserved in FAD-dependent halogenases (residues 1–389). FAD and halide were not found in the FAD binding site, consistent with the lack of color of the protein and its crystals (Supplementary Fig. 32). The C-terminal quarter of the protein is a unique helical region not found in other halogenases (Supplementary Fig. 33). The putative substrate binding cleft located in the interface of the FAD binding fold and the C-terminal region leads to a conserved catalytic lysine residue (Lys87), based on structural superimposition of PltM with structures of Trp halogenases bound to l-Trp (Supplementary Fig. 33). Indeed, mutating Lys87 to an alanine yielded a catalytically inactive protein (Supplementary Fig. 34). The C-terminal region then likely helps define the substrate specificity. Soaking crystals of PltM with compound **1** yielded a strong and featureful polder omit *mF*_o_–*DF*_c_ electron density^25^ in three out of four substrate binding sites in the asymmetric unit, corresponding to a molecule of compound **1** and a water molecule that bridged it with the protein (Fig. 4b). The binding site of compound **1** is analogous to that of l-Trp in the crystal structure of RebH^14^ and PrnA^16^ (Supplementary Fig. 33). The nearest carbon atom of compound **1** that can be halogenated is ~4.5 Å away from the Nε of Lys87, further supporting the model. At its entrance, the substrate binding cavity is lined by charged and polar side chains (Glu115, Glu49, Lys501, and Asn405) (Fig. 4b), which would interact favorably with hydroxyl and amino groups on PltM substrates indicated by the activity profile (Fig. 3b). One face of the phenyl ring of **1** is in nonpolar contacts with and Pro48 and Leu111 and the other face stacks approximately orthogonally Trp400 and interacts with Leu401. The phenyl ring of compound **1** is stacked nearly orthogonally against Phe90. This residue likely helps orient the substrate for halogenation. The hydroxyl groups of bound **1** are within hydrogen bonding distances from the side chains of Lys501, Asn405, Glu49, Ser404 and the main chain nitrogen of Phe90 and one hydroxyl is bridged to a carbonyl oxygen of Ile499 by a water molecule. These interactions underscore the importance of the unique C-terminal region in substrate recognition. The substrate binding site is large enough to accommodate a diiodinated **1** (Supplementary Fig. 35). The substrate binding site is situated relatively close to the protein surface, which could allow access to larger substrates, like resveratrol (**23**). The halogenation center is nevertheless restricted by the helical C-terminal region to addition of up to two halogens; a trihalogenated product cannot be sterically accommodated and neither can halogenated l-Trp (Supplementary Fig. 33).

### Crystal structures of PltM with FAD bound in different states

PltM represents a type of FAD-dependent enzymes, where FAD dissociates out of its binding site for reduction. To gain structural insight into this enigmatic process, we sought to determine a crystal structure of PltM–FAD complex by soaking the crystals of apo PltM with FAD. We obtained two different crystal forms of PltM–FAD complexes, where a molecule of FAD was bound to PltM in two different states (Fig. 4c, d, Supplementary Table 4). In one state, an FAD molecule was bound at a site and orientation analogous to those observed in structures of other FAD-dependent halogenases, where the isoalloxazine group of the FAD was fully encased by the enzyme (Fig. 4c, Supplementary Fig. 36). A chloride ion was well resolved at a conserved site near the FAD. In the other state, the FAD molecule was bound near the mouth of the FAD binding cleft, with the clearly resolved isoalloxazine ring in the same plane, but oriented perpendicularly to the fully bound state, also making extensive contacts with the protein (Fig. 4d). The electron density for the rest of the FAD molecule is not observed due to disorder (Supplementary Fig. 37), as in this state the adenine nucleotide moiety is directed into the solvent. This structure may represent an intermediate between the apo and the fully bound FAD state. The crystals of PltM–FAD complexes in this state belong to the same crystal form as the crystals of all other complexes in this study; therefore, crystal packing interactions have no effect on the FAD binding state. A short nonconserved loop containing three Ala, a Gly and a Ser (residues 172–178) and the side chain of Gln321 are in two different conformations in these two structures (Fig. 4c, d). In the holoenzyme state, the loop and Gln321 form one side of the narrow cleft holding the adenine nucleotide portion of FAD in place: the side chain of Ala173 interacts with the adenine ring of the FAD, Ala174 interacts with the phosphosugar bridge, and the aliphatic portion of Gln321 holds the riboflavin bridge. In the state with partially bound FAD, this cleft is collapsed, and filled with water. In this state, the isoalloxazine ring is sandwiched between Phe325 and the backbone of loop residues Ala174 and Gln175, including the C_β_ of the latter residue. The FAD binding pocket does not contain a Cl^−^, indicating that a halide ion binds upon the final steps of FAD binding. Previous kinetic experiments with RebH^26^ and *p*-hydroxybenzoate hydroxylase^27^ suggested that kinetically significant conformational changes involving FAD dynamics occurred in FAD recycling. For both enzymes, it was proposed that a distinct mechanistically important state exists where the flavin ring of FAD can undergo redox chemistry, while being sufficiently shielded away from the solvent. This structure may represent such intermediate; future extensive kinetic studies probing the FAD recycling mechanism are needed to address this directly.

### Halogenation assays in fermentation culture

**Fig. 5:**
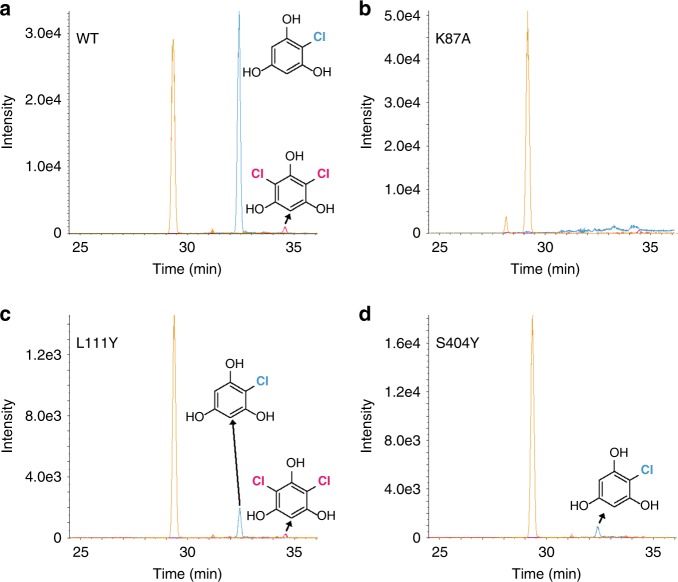
Halogenation by PltM and its mutants in a cell-based assay. XIC traces of the cell-based halogenation assay using wild-type PltM (**a**), PltM K87A (**b**), PltM L111Y (**c**), and PltM S404Y (**d**). The blue trace refers to the mono-chlorinated **1** while the pink trace shows the dichlorinated **1**. The orange trace refers to unmodified starting compound **1**

As a preliminary assessment of potential use of PltM in a fermentation setting, we tested whether phloroglucinol (**1**) can be halogenated upon adding it to the culture of *Escherichia coli* BL21(DE3) overexpressing PltM. We also validated the substrate binding cavity observed in the crystal structures by testing halogenation by two point mutants of PltM, L111Y and S404Y, in this setting. These two residues (one from the FAD binding fold and one from the C-terminal region) line the substrate binding cavity, and their bulkier substitutions are predicted to block binding of **1** (Supplementary Fig. 38). In addition, as negative controls, we used PltM K87A that was demonstrated to be inactive in vitro as well as PltA. All five proteins were expressed at the same level. The cells expressing wild-type PltM generated mono- and dichlorinated **1** (Fig. 5, Supplementary Fig. 39). No halogenated product was observed in cultures expressing PltM K87A and PltA, validating the PltM as the sole source of halogenation activity. For the cells expressing L111Y and S404Y mutants, the product yield was significantly reduced compared to wild-type; the effect of the S404Y mutation was especially severe. We determined a crystal structure of PltM L111Y, which showed that the overall protein structure is unperturbed and the only effect of the mutation was to obstruct the access to the substrate binding pocket, as predicted (Supplementary Fig. 38). S404Y caused a more drastic effect than L111Y because Y404 was predicted to sterically clash with the bound substrate. These data further validated the structure-based definitions of the substrate binding site and suggested a potential for halogenation in a fermentation setting. Future fermentation experiments to maximize the yield of halogenated product will require optimization of the producing host strain and other culture growth conditions.

### Kinetics and regiospecificity of PltM in optimized reactions

For quantitative analysis of enzyme kinetics and detailed structural characterization of reaction products, as well as for potential future biotechnological use, we extensively optimized in vitro enzymatic reaction conditions and coupled enzymes to maximize product yield. The critical factors of the optimized conditions were introducing glucose dehydrogenase (GDH) for NADPH regeneration^28^ and lowering the concentrations of NADPH and halide salts. This optimization significantly improved reaction yields, resulting in full conversion of several substrates (Supplementary Table 7). This additional information corroborated the preference for substrates containing electron withdrawing groups and showed preference of PltM for substrates with 1- and 3-hydroxyl or amino groups. We determined the halide preference and evaluated quantitatively the kinetics of chlorination and bromination of substrates **3**, **11**, and **16**, which showed 100% conversion upon overnight reaction (Table 1 and Supplementary Fig. 40). Kinetic of iodination could not be analyzed quantitatively due to gradual enzyme precipitation in the presence of iodide. These data indicated that PltM preferred chlorination for all substrates that were eventually dichlorinated. The preference for bromination versus iodination depended on particular substrates, with **3** and **16** showing preference for bromination, and **11** for iodination. In fact, both **3** and **16** were dibrominated by PltM. Chlorination and bromination of **3** and **16** occurred with similar efficiencies, whereas **11** was chlorinated much better than brominated or iodinated. Interestingly, two mono-halogenated products were observed for iodination of **11** and for halogenation of **16**. No fluorination was still observed for any substrates at the optimized conditions.

**Table 1:** Kinetic parameters for halogenations of selected substrates

Substrate	Halogen	Substrate/product	*k*_cat_ (min^−1^)	*K*_m_ (μM)	*k*_cat_/*K*_m_ (min^−1^ μM^−1^)
**3**	Cl	**3**/Cl-**3**	2.3 ± 0.1^a^	(7.6 ± 1.3) × 10^−2^	30 ± 5
		Cl-**3**/diCl-**3**	0.40 ± 0.01	0.11 ± 0.02	3.5 ± 0.6
	Br	**3**/Br-**3**	1.9 ± 0.1	0.71 ± 0.2	2.7 ± 0.6
		Br-**3**/diBr-**3**	0.38 ± 0.01	0.78 ± 0.17	0.49 ± 0.11
**11**	Cl	**11**/Cl-**11**	1.6 ± 0.1	8.4 ± 0.4	0.19 ± 0.01
		Cl-**11**/diCl-**11**	0.18 ± 0.01	0.44 ± 0.02	0.41 ± 0.02
	Br	**11**/Br-**11**	0.24 ± 0.02	(1.1 ± 0.2) × 10^3^	(2.2 ± 0.2) × 10^−4^
**16**	Cl	**16**/Cl-**16a**^b^	0.35 ± 0.01	1.1 ± 0.3	0.31 ± 0.09
		**16**/Cl-**16b**	0.22 ± 0.01	1.1 ± 0.3	0.19 ± 0.06
		Cl-**16a**/diCl-**16**	0.95 ± 0.01	18 ± 5	(5.2 ± 1.4) × 10^−2^
		Cl-**16b**/diCl-**16**	1.0 ± 0.1	15 ± 9	(7.0 ± 4.4) × 10^−2^
	Br	**16**/Br-**16a**	0.47 ± 0.04	151 ± 20	(3.1 ± 0.5) × 10^−3^
		**16**/Br-**16b**	2.2 ± 0.1	151 ± 20	(1.5 ± 0.2) × 10^−2^
		Br-**16a**/diBr-**16**	0.21 ± 0.02	6.0 ± 1.2	(3.5 ± 0.8) × 10^−2^
		Br-**16b**/diBr-**16**	2.0 ± 0.1	(1.3 ± 0.1) × 10^3^	(1.5 ± 0.2) × 10^−3^

^a^The values of all mono-halogenation and dihalogenation rate constants *k*_cat,1_ and *k*_cat,2_, respectively, and *K*_m_ for mono-halogenation and dihalogenation (defined as (*k*_d,1_ + *k*_cat,1_)/*k*_a,1_ and (*k*_d,2_ + *k*_cat,2_)/*k*_a,2_, respectively) were determined by nonlinear regression using DynaFit, as described in Methods

^b^Two distinct mono-halogenation products of the same reaction are denoted by labels **a** and **b**

**Fig. 6:**
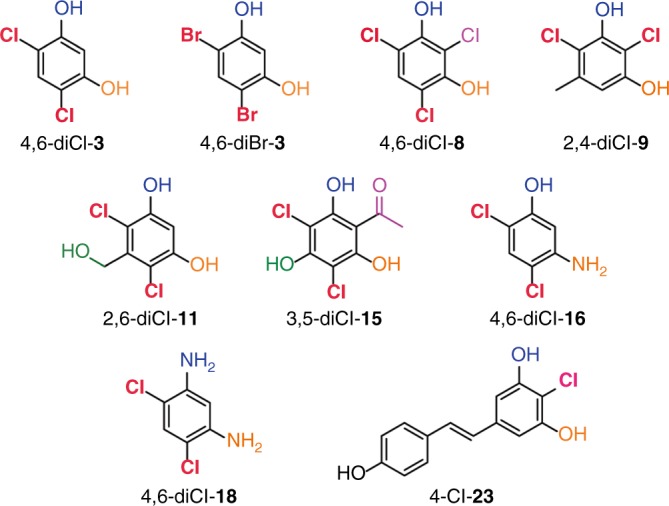
Structures of the products of halogenation by PltM. Structures, as determined by NMR spectroscopy, of products resulting from the halogenation of compounds **3**, **8**, **9**, **11**, **15, 16**, **18**, and **23** by PltM

The high yield of chlorination and bromination of these and several other compounds allowed us to establish the regiospecificity of the halogenation by PltM. However, some substrates or products were insufficiently stable during halogenation reactions precluding their quantitative structural analysis. We determined the structures of the final dichlorinated products of **3**, **8**, **9**, **11**, **15**, **16**, **18**, as well as the monochlorinated product of **23** and the dibrominated product of **3** by NMR spectroscopy. The resulting products were 4,6-diCl-**3**, 4,6-diCl-**8**, 2,4-diCl-**9**, 2,6-diCl-**11**, 3,5-diCl-**15**, 4,6-diCl-**16**, 4,6-diCl-**18**, 4-Cl-**23**, and 4,6-diBr-**3**, respectively (Fig. 6 and Supplementary Figs. 42–59). These structures were consistent with the time course experiments showing one mono-halogenated intermediate for symmetrical substrates **3** and **11** and two mono-halogenated intermediates for asymmetrical substrate **16**. Likewise, for most other substrates (**8**, **15**, and **18**) the structures of the respective monochlorinated intermediates are unambiguously inferred owing to the product symmetry. These results show that for mono- or di-hydroxylated or aminated substrates, PltM halogenates almost exclusively in *ortho* to these polar groups, but not between them. However, when a methyl or a styrene moiety was found in *meta* to two hydroxyls, as in compound **9** (which was dichlorinated) and resveratrol (**23**; which was monochlorinated), respectively, we observed that a chlorination event occurred between the two hydroxyls.

### Development of an immobilized halogenating system

**Fig. 7:**
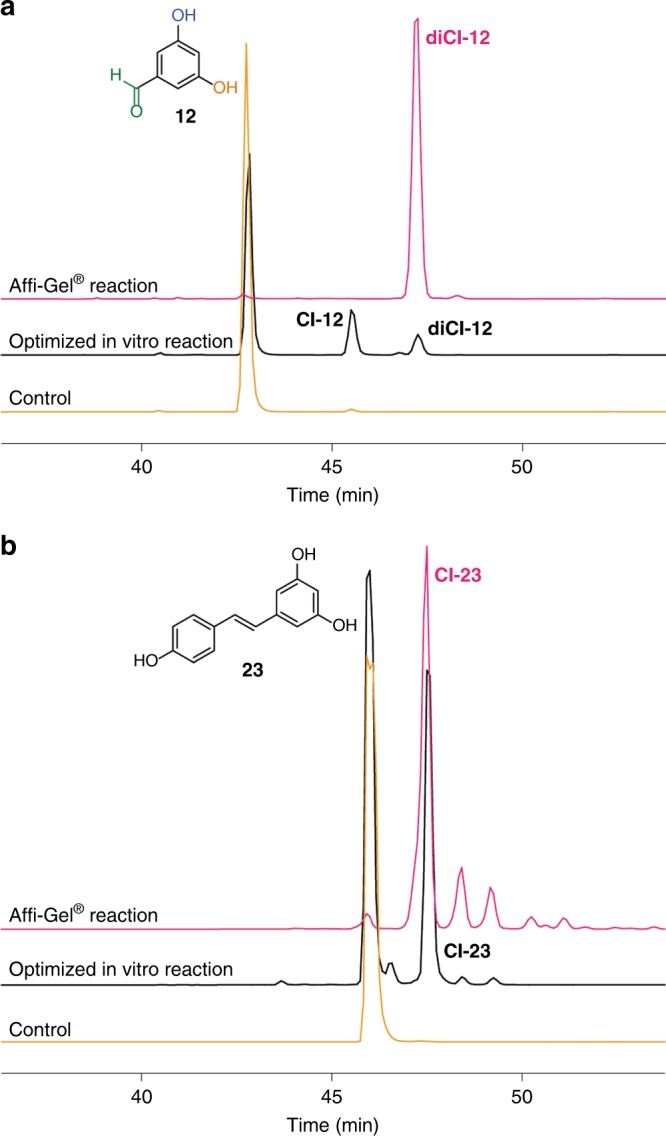
HPLC chromatograms of chlorination reactions by PltM with substrates. Reaction with **a** **12** and **b 23** prior (black traces) and after optimization employing Affi-Gel® resin (pink traces)

The halogenation yield is limited by the stability of proteins, with PltM being the limiting factor. To achieve a more efficient and scalable halogenation reaction, we developed a method, where all three proteins were immobilized on agarose resin (Affi-Gel® 15), packed into a spin column and then used as a resin conjugate for halogenation. The halogenation reactions were performed by adding substrate and reagents into the column. This protein-bound resin showed a high halogenation yield for some compounds, which could not be efficiently halogenated by free enzymes in solution (Fig. 7). Notably, the enzyme–resin conjugate could be reused 5–6 times without significant loss of efficiency (Supplementary Fig. 41).

The remarkable halide versatility for any FAD-dependent halogenase and very broad substrate profile for a phenolic halogenase call for future exploration of PltM as a halogenation tool. Our structures revealed a unique architecture of this enzyme, and an FAD orientation that may be relevant to the FAD recycling mechanism shared by FAD binding enzymes.

## Methods

### Materials and instrumentation

The PltM, SsuE, and PltA (used as a control in this study) proteins were overexpressed and purified based on our previously described protocols^23,24^. DNA primers for PCR were purchased from Integrated DNA Technologies (IDT; Coralville, IA, USA). Restriction enzymes, Phusion DNA polymerase, and T4 DNA ligase were purchased from New England BioLabs (NEB; Ipswich, MA, USA). All chemicals and buffer components were purchased from Sigma-Aldrich or VWR (Radnor, PA, USA) and used without any further purification. Size-exclusion chromatography was performed on a fast protein liquid chromatography (FPLC) system BioLogic DuoFlow (Bio-Rad; Hercules, CA, USA) by using a HiPrep 26/60 S-200 HR column (GE Healthcare, Piscataway, NJ, USA). LC-MS was performed on a Shimadzu high-performance liquid chromatography (HPLC) system equipped with a DGU-20A/3R degasser, LC-20AD binary pumps, a CBM-20A controller, a SIL-20A/HT autosampler (Shimadzu, Kyoto, Japan), and Vydac HPLC DENALI™ Column (C_18_, 250 × 4.6 mm, 5 μm particle size) from Grace (Columbia, MD, USA) and an AB SCIEX TripleTOF 5600 (AB SCIEX, Redwood City, CA) mass spectrometer recording in negative or positive mode between 80 and 600 *m/z*. HPLC was performed on an Agilent Technologies 1260 Infinity system equipped with a Vydac HPLC DENALI™ column (C_18_, 250 × 4.6 mm, 5 μm particle size) and an Alltech Econosil HPLC column (C_18_, 250 × 10 mm, 10 μm particle size; Grace) for analytical and semi-preparative experiments, respectively. ^1^H and ^13^C NMR spectra were recorded at 400 and 500 (for ^1^H) as well as 100 MHz (for ^13^C) on Varian 400 and 500 MHz spectrometers, using deuterated solvents as specified. Chemical shifts (*δ*) are given in parts per million (ppm). Coupling constants (*J*) are given in Hertz (Hz), and conventional abbreviations used for signal shape are as follows: s, singlet; d, doublet; t, triplet; m, multiplet; q, quartet.

### Synthesis of compound 15

Aluminum chloride (1.3 g, 9.99 mmol) was slowly added to a solution of phloroglucinol (**1**, 315 mg, 2.50 mmol) in 1:1/1,2-dichloroethane:nitrobenzene (10 mL) at 0 °C. After stirring this mixture at this temperature for 10 min under a nitrogen atmosphere, acetyl chloride (0.21 mL, 3.00 mmol) was added. Then the ice bath was removed, and the mixture was stirred at 80 °C for 2 h. The reaction progress was monitored by TLC (1:2/EtOAc:Hexanes, *R*_*f*_ 0.35). The reaction mixture was quenched with H_2_O (60 mL), extracted with EtOAc (2 × 100 mL), washed with brine (20 mL), and then dried over MgSO_4_. The organic layer was removed under reduced pressure and the residue was purified by flash column chromatography (SiO_2_, 1:2/EtOAc:Hexanes) to afford the known compound **15**^30^ (223 mg, 53%) as a yellow solid: ^1^H NMR (400 MHz, CD_3_OD) δ 5.78 (s, 2H), 2.58 (s, 3H); ^13^C NMR (100 MHz, (CD_3_)_2_SO) δ 203.1, 164.9, 164.5, 104.2, 94.1, 31.3.

### PltM mutagenesis

PltM mutants K87A, L111Y, and S404Y were constructed by splicing-by-overlap-extension method^31^. The sequences downstream and upstream of the mutation site were amplified first individually from p*pltM*-pET28a(NHis). For PltM K87A mutant; the primer pairs were #1: 5′-CGCCTGCGGGATCgcgCTGGGCTTCAGTTTTG-3′ with #2: 5′-CATACTCGAGCTAGACTTTGAGGATGAAACGATTG-3′ and #3: 5′-CAAAACTGAAGCCCAGcgcGATCCCGCAGGCG-3′ with #4: 5′-GCAGCTCTCATATGAATCAGTACGACGTCATTATC-3′. For PltM L111Y mutant; the primers were #5: 5′-CTTGTGGCCCCGCCGtatAAGGTGCCGGAAGCC-3′ with #2 and #6: 5′-GGCTTCCGGCACCTTataCGGCGGGGCCACAAG-3′ with #4. For PltM S404Y mutant, the primer pairs were #7: 5′-CTGGCTCAGCGGCtatAACCTGGGCAGTGC-3′ with #2 and #8: 5′-GCACTGCCCAGGTTataGCCGCTGAGCCAG-3′ with #4. The PCR products of the above primer pairs were used as templates for another round of PCR using primers #2 and #4. The products from the second round of PCR were digested with restriction enzymes *Nde*I and *Xho*I and ligated into *Nde*I/*Xho*I-linearized pET28a, yielding p*pltM*K87A-pET28a, p*pltM*L111Y-pET28a, and p*pltM*S404Y-pET28a. The mutations were verified by DNA sequencing (Eurofins Genomics).

### Preparation of p*gdh*-pET28a overexpression construct

The glucose dehydrogenase (*gdh*) gene was amplified from genomic DNA of *Bacillus subtilis* subsp. *subtilis* 168 by PCR with the forward and reverse primers: 5′-AGGATGCATATGTATCCGGATTTAAAAGGAAAAG-3′ and 5′-CGCTTTCTCGAGTTAACCGCGGCCTGCCTGGAAT-3′, respectively. The PCR product was purified by agarose gel extraction and digested by restriction enzymes *Nde*I and *Xho*I, which was subsequently ligated into *Nde*I/*Xho*I-linearized pET28a. The resulting plasmid p*gdh*-pET28a was transformed into a chemically competent *E. coli* TOP10 strain, and the cloning was verified by sequencing of the purified plasmids.

### Preparation of PltM and coupled enzymes for in vitro assays

Open reading frames encoding PltM and FAD reductase SsuE were cloned into *E. coli* expression vectors as previously reported^23^. For the production of PltM, SsuE, and GDH, the expression vectors were transformed into *E. coli* BL21(DE3) (ATCC; Manassas, VA). In each case, a colony was grown overnight at 37 °C with shaking at 200 rpm in LB medium (5 mL) supplemented with 50 μg/mL kanamycin. These overnight cultures were inoculated into LB medium (1 L) supplemented with 50 μg/mL kanamycin. Cultures were grown (37 °C, 200 rpm) until an attenuance at 600 nm of 0.6 was reached. At this time, protein expression was induced by adding isopropyl-β-d-1-thiogalactopyranoside (IPTG, 0.2 mM), and the cultures were incubated at 16 °C with shaking at 200 rpm for an additional 20 h. The cells were harvested by centrifugation at 3000×*g* for 10 min at 4 °C. The cell pellets were washed with buffer A (50 mM sodium phosphate pH 7.4, 400 mM NaCl, 5 mM imidazole, and 10% glycerol). The cells were resuspended in 40 mL of buffer A supplemented with 1 mM dithiothreitol (DTT) and 1 mM phenylmethanesulfonyl fluoride (PMSF). The cells were then lysed by intermittent sonication, followed by clarification by centrifugation at 40,000×*g* for 45 min at 4 °C. The supernatants were incubated with 0.5 mL of pre-washed Ni^II^-NTA agarose resin (Qiagen, Valencia, CA) at 4 °C for 2 h with slow tumbling. The slurry was loaded onto a column and washed with 2 × 5 mL of buffer A followed by elution with a gradient of imidazole concentration in buffer A (2 × 5 mL of 20 mM, 5 mL of 40 mM, 5 mL of 60 mM, 2 × 5 mL of 250 mM). Fractions containing pure proteins were combined and dialyzed against 3 × 2 L of buffer B (50 mM sodium phosphate pH 7.4, 2 mM β-mercaptoethanol (βME), and 10% glycerol). Each of the three dialysis steps was performed at least for 4 h. The dialyzed proteins were concentrated to ~20 mg/mL for PltM and GDH or ~2.5 mg/mL for SsuE by using Amicon Ultra-15 Centrifugal Filter Units (EMD Millipore, Billerica, MA, USA) with 10-kDa molecular weight cutoff (MWCO) for PltM and GDH or 3-kDa MWCO for SsuE, and protein concentrations were determined by absorbance at 280 nm with calculated extinction coefficients *ε* = 59,840, 20,340, and 29,910 M^−1^ cm^−1^ for PltM, SsuE, and GDH, respectively (http://protcalc.sourceforge.net/cgi-bin/protcalc). The total yields of pure PltM, SsuE, and GDH were 17.6, 6.0, and 10.3 mg from 1 L of culture, respectively. The proteins were flash frozen in liquid nitrogen and stored at −80 °C for biochemical assays. The point mutants of PltM were purified by using the above protocol for the full-length PltM.

### Preparation of PltM for crystallography

Wild-type PltM and PltM L111Y mutant were purified as described above with an additional size-exclusion chromatography step. Wild-type PltM and PltM L111Y eluted from Ni^II^ resin were loaded onto an S-200 column equilibrated in 40 mM Tris–HCl, pH 8.0, 100 mM NaCl, 2 mM βME. Fractions containing NHis_6_-PltM were pooled and concentrated to 40 mg/mL by using an Amicon Ultra-15 Centrifugal Filter Unit with 10 kDa MWCO. Purified PltM proteins were kept on ice for crystallization studies.

### In vitro assays of PltM with various substrates and halides

The halogenation assays were carried out similarly to a recently described procedure^22^. The substrates that have been tested are given in Fig. 3a and Supplementary Fig. 7.

For substrate profile determination (Assay 1), 100 μL reactions were carried out in 30 mM sodium phosphate pH 7.4. As a halide source, we used 200 mM of either NaF (Assay 1a), NaCl (Assay 1b), NaBr (Assay 1c), or NaI (Assay 1d). To ensure that PltM is incapable of fluorinating, an additional 200 μL reaction with 300 mM NaF was run. We also ran 200 μL reaction with 400 mM NaBr to ensure no additional bromination reaction occurred. Each reaction also contained a specified substrate (0.5 mM), FAD (0.2 mM), NADPH (5 mM), PltM (5.5 μM), and SsuE (5.0 μM). The reactions were initiated by adding NADPH under N_2_. The reaction tubes were tightly closed to avoid contact with air. The reaction mixtures were incubated at 25 °C for 3 h prior to extraction with EtOAc (4 × 100 μL). The organic layer was dried by a gentle flow of air, and the residue was dissolved in MeOH to prepare 1–10 μg/mL samples for LC-MS analysis.

To establish if hetero-dihalogenation by PltM could be observed, halogenating competition assays in 1:1 or 10:1 mixture of two different halide salts were performed (Assay 2). The reactions contained the same components as above except single halide salts were replaced with either a 1:1/NaCl:NaBr (Assay 2a), 1:1/NaCl:NaI (Assay 2b), or 1:1/NaBr:NaI (Assay 2c) mixtures (100 mM of each halide). The reactions were initiated by adding NADPH under N_2_. A 1:1/NaCl:NaBr reaction was also performed with 200 mM of each halide to test the occurrence of homo-di- or hetero-chlorination/bromination, and 10:1/NaCl:NaI (Assay 2d) and 10:1/NaBr:NaI (Assay 2e) mixtures with 200 mM of NaCl or NaBr and 20 mM of NaI were tested to check whether chlorination or bromination could occur in the presence of iodide and whether iodination can occur with chlorination or bromination to yield a Cl,I-substrate or Br,I-substrate. The reactions were incubated and processed as described above in Assay 1.

### Optimized in vitro PltM halogenation assay

To increase the production of halogenated molecules and decrease the amount of NADPH required, the above in vitro assay was optimized by using an additional enzyme, GDH^28^. The optimized reaction mixture contained substrate (0.5 mM for chlorination and bromination; 0.25 mM for iodination; prepared from 50 mM stock in DMSO), FAD (5 μM), NADPH (5 μM), PltM (6 μM), SsuE (5 μM), GDH (0.5 μM), glucose (20 mM), NaX (10 mM for chlorination and bromination; 0.5 mM for iodination), and sodium phosphate (30 mM, pH 7.4), and was incubated at room temperature. The overall yield of halogenation products was determined for reactions run overnight for several substrates (Supplementary Table 7). Conversion of the substrate to halogenated products was monitored by HPLC at *λ* = 275–320 nm, where the absorbance of molecules is not affected by halogenation, and quantified as fraction of reaction species (%). The time course experiments for kinetic analysis were performed in 100 μL reaction mixtures by quenching the reactions at 0, 5, 15, 30, 60, 120, 240, and 360 min (for **3** and **16**), and an additional 720 min (for **11**) for chlorination and bromination, and at 0, 30, 60, 120, 240, and 480 min (for **3** and **16**), or an additional 720 min (for **11**) for iodination. The time course experiments were performed in duplicate. Compound **1** was unstable under these optimized conditions, and it was not tested. The in vitro analysis of K87A mutant was performed overnight in 100 μL reaction mixture by using compound **11** as a substrate. Wild-type PltM was used as a positive control, and no enzyme reaction was used as a negative control. In all the above reactions, the compounds were extracted with EtOAc (4 × 100 μL) and dried under gentle air flow. The products were dissolved in MeOH (30 μL for chlorination and bromination; 15 μL for iodination) for HPLC analysis. The scale-up experiments were performed overnight in 25 mL for compound **23**, in 50 mL for compounds **3**, **8**, **9, 11**, **15**, and **18**, or 100 mL for **16**. PltM concentration was 25 μM with compounds **15** and 250 μM with compound **23**. To process the chlorination reaction of compound **23**, ice-cold MeOH (50 mL) was added to precipitate the proteins. This mixture was incubated for 2 h at −20 °C, and the protein precipitate was removed by centrifugation (40,000×*g*, 30 min, 4 °C). The pellet was washed by ice-cold MeOH (50 mL) and centrifuged down again (40,000×*g*, 15 min, 4 °C). The supernatant was combined in a round-bottomed flask, and MeOH was removed in vacuo. The products were extracted with EtOAc (4 × reaction volume) and dried in vacuo. These were dissolved in MeOH (0.5–1 mL) for purification by semi-preparative HPLC.

### Halogenation assay using immobilized enzymes

To increase the yield of halogenation reaction and make the enzymes reusable, PltM, SsuE, and GDH, we immobilized these proteins on Affi-Gel® 15 resin (Bio-Rad, Hercules, CA). To increase the stability of the coupled enzymes, GDH from *Bacillus amyloliquefaciens* SB5 (GDH-BA)^32^ was used in this assay. This enzyme was expressed and purified, as described above, from a pET23a vector (*amp*^R^) containing a synthetic gene encoding this enzyme (NCBI accession #JQ305165) with an NHis_6_ tag, purchased from GenScript (Piscataway, NJ). The enzymes were dialyzed into buffer C, which contains HEPES (50 mM, pH 7.5), βME (2 mM), and glycerol (10%). Suspended Affi-Gel® resin (250 μL) was transferred into a QIAquick spin column (Qiagen), and the resin was washed three times with 500 μL of H_2_O and buffer D (30 mM HEPES, pH 7.5). For each time, the wash solution was removed by centrifugation (400×*g*, for 15–30 s, 4 °C). The washed resin was incubated with SsuE (~50 μM, 300 μL) for 4 h at 4 °C. The beads were washed with buffer D twice and subsequently incubated with a mixture of GDH-BA (~200 μM, 50 μL) and PltM (~500 μM, 250 μL) overnight at 4 °C. This resin–enzyme conjugate was washed twice with buffer D and preserved in 4 °C in buffer D until needed. For each 250 μL resin, 300 μL of reaction solution, which contained substrate (0.5 mM), FAD (5 μM), NADPH (5 μM), glucose (20 mM), NaCl (10 mM), and HEPES (30 mM, pH 7.5), was used. The reaction with resveratrol (**23**) was performed overnight at room temperature. The reaction solution was collected by centrifugation (400 × *g*, every 15–30 s until the solution was removed, 4 °C), and the resin–enzyme conjugate in the column was washed with buffer D (300 μL) three times. These solutions were extracted with EtOAc (4 × 300 μL) and dried in vacuo. The solid material was dissolved in MeOH (200 μL) and analyzed by HPLC (Fig. 7). The reusability of the resin–enzyme conjugate was tested with substrates **3** and **11** (Supplementary Fig. 41). The reactions (same as above) were run for 1 h at room temperature and processed as described above. After processing the reaction, the same reaction was repeated four more times. After the 5th reaction, the beads were stored at 4 °C overnight in buffer D. The 6th–10th reactions were performed in the following day.

### Kinetic analysis of PltM halogenation

To determine the halogenation preference, the kinetic parameters were obtained by the global nonlinear regression analysis of all reaction species using DynaFit software^29^ for the following halogenation mechanism:

1 $$E + S\begin{array}{*{20}{c}} {k_{a,1}} \\ \rightleftarrows \\ {k_{d,1}} \end{array}E \cdot S$$

2 $$E \cdot S\mathop{\longrightarrow}\limits^{{k_{cat,1}}}E \cdot P_1$$

3 $$EP_1 + S\begin{array}{*{20}{c}} {k_{a,2}} \\ \rightleftarrows \\ {k_{d,2}} \end{array}EP_1 \cdot S$$

4 $$EP_1 \cdot S\mathop{\longrightarrow}\limits^{{k_{cat,2}}}E + P_2$$

where *E*, *S*, *P*_1_, and *P*_2_ are enzyme, substrate, mono-, and dihalogenated product, respectively.

### Cell-based activity assay of PltM

*E. coli* BL21(DE3) cells were transformed with p*pltM*-pET28a, p*pltM*K87A-pET28a, p*pltM*L111Y-pET28a, p*pltM*S404Y-pET28a, and p*pltA*-pET28a. The p*pltA*-pET28a plasmid overexpressing the halogenase PltA whose substrate is pyrrolyl-*S*-PltL (a peptidyl carrier protein-linked pyrrole) was used as a negative control^23^. Five colonies from each transformant were cultured in 2 × 500 mL of LB medium (for p*pltM*-pET28a, p*pltM*L111Y-pET28a, and p*pltM*S404Y-pET28a) and 1 × 500 mL of LB medium (for p*pltM*K87A-pET28a and p*pltA*-pET28a) with 50 μg/mL kanamycin at 37 °C and 200 rpm until attenuance of 0.2 at 600 nm. The cultures were then moved to 25 °C until attenuance of 0.5. Protein expression was induced by adding 0.2 mM IPTG to all seven flasks, and the cultures were incubated with shaking for 1 h. 12.5 μg/mL of compound **1** was added to 1 × 500 mL of LB medium containing p*pltM*-pET28a, p*pltM*K87A-pET28a, p*pltM*L111Y-pET28a, p*pltM*S404Y-pET28a, and p*pltA*-pET28a. Compound **1** was not added to the three remaining flasks (negative controls). After additional incubation for 20 h, the cells were pelleted at 5000×*g* for 10 min, and the supernatant was collected. The supernatant was extracted with EtOAc (3 × 330 mL), which was dried in vacuo. This was then dissolved in MeOH (100 μL) prior to addition of H_2_O (800 μL) followed by centrifugation at 20,000×*g* for 10 min to remove the precipitate. The supernatant was collected and 1 μL was diluted into 199 μL of MeOH for LC-MS analysis (Supplementary Table 6).

### HPLC and LC-MS analysis of halogenated products

The halogenation reaction products were analyzed by HPLC or LC-MS by injecting 10 μL of each sample. The compounds were separated by reversed-phase HPLC at the flow rate of 0.2 mL/min by using the following program: eluent A = H_2_O; eluent B = MeCN; gradient = 2% B for 5 min, increase to 100% B over a 30 min period, stay at 100% B for 9 min, decrease to 2% B over a 1 min period, and re-equilibrate the column at 2% B for 30 min. For HPLC analysis, the molecules were observed by absorbance at *λ* = 275 nm as described above. As necessary, the following mass spectrometer was operated in negative and positive modes with the following parameters: for negative mode, mass range, 80–600 *m*/*z* in profile mode; temperature, 550 °C and ion spray voltage floating, −4500 V, and for positive mode, mass range, 80–600 *m*/*z* in profile mode; temperature, 550 °C and ion spray voltage floating, 4500 V. The presence of each compound was analyzed by extracted ion chromatograph (XIC) with the expected mass ±0.05 Da for Assay 1 and Assay 2 and ±0.005 Da for Assay 3 (Fig. 2a–c, Supplementary Figs. 1–6 and 10–30, and Supplementary Tables 2, 3, and 6). The LC-MS was operated by Analyst TF Software (SCIEX, Framingham, MA), and the data was analyzed by PeakView (SCIEX). To purify 4 selected scaled-up halogenated products, semi-preparative HPLC was performed by injecting 100 μL per injection at 1 mL/min by using the following gradient program with eluent A as H_2_O (with 0.1% TFA) (for compounds **3** and **11**) or 10 mM ammonium bicarbonate (for **16**) and eluent B as MeCN: 2% B for 10 min, increase to 100% B over a 40 min period, stay at 100% B for 5 min, decrease to 2% B over a 1 min period, followed by re-equilibration in 2% B for 9 min. The collected peak fractions were dried under reduced pressure and lyophilized for NMR analysis.

### NMR analysis of products of large-scale halogenation

The exact position for the various halogenations was determined either by comparison with commercially available standards (4,6-dichlororesorcinol) or by a combination of HMBC and HSQC experiments.

The analysis of halogenation products is presented as follows.

Analysis of 4,6-dichlororesorcinol (4,6-diCl-**3**): ^1^H NMR (500 MHz, CD_3_OD, Supplementary Fig. 42) δ 7.17 (s, 1H), 6.52 (s, 1H).

Analysis of 4,6-dibromoresorcinol (4,6-diBr-**3**): ^1^H NMR (500 MHz, CD_3_OD, Supplementary Fig. 43) δ 7.45 (s, 1H), 6.53 (s, 1H); ^13^C NMR (100 MHz, CD_3_OD, Supplementary Fig. 44) δ 154.1, 134.8, 103.5, 99.3.

Analysis of 2,4,6-trichlororesorcinol (4,6-diCl-**8**): ^1^H NMR (500 MHz, CD_3_OD, Supplementary Fig. 45) δ 7.23 (s, 1H).

Analysis of 2,4-dichloro-5-methylresorcinol (2,4-diCl-**9**): ^1^H NMR (500 MHz, CD_3_OD, Supplementary Fig. 46) δ 6.43 (q, *J* = 0.5 Hz, 1H), 2.39 (d, *J* = 0.5 Hz, 3H); ^13^C NMR (100 MHz, CD_3_OD, Supplementary Fig. 47) δ 151.9, 134.6, 112.0, 110.0, 101.3, 16.5.

Analysis of 2,6-dichloro-3,5-dihydroxybenzyl alcohol (2,6-diCl-**11**): ^1^H NMR (500 MHz, CD_3_OD, Supplementary Fig. 48) δ 6.55 (s, 1H), 4.85 (s, 2H); ^13^C NMR (100 MHz, CD_3_OD, Supplementary Fig. 49) δ 152.3, 136.2, 112.8, 103.4, 58.9. The HMBC for 2,6-dichloro-3,5-dihydroxybenzyl alcohol is presented in Supplementary Fig. 50.

Analysis of 3,5-dichloro-2,4,6-trihydroxyacetophenone (3,5-diCl-**15**): ^1^H NMR (500 MHz, CD_3_OD, Supplementary Fig. 51) δ 2.69 (s, 3H).

Analysis of 5-amino-2,4-dichlorophenol (2,4-diCl-**16**): ^1^H NMR (400 MHz, CD_3_OD, Supplementary Fig. 52) δ 7.06 (s, 1H), 6.38 (s, 1H); ^13^C NMR (100 MHz, CD_3_OD, Supplementary Fig. 53) δ 152.4, 143.8, 128.7, 109.4, 108.7, 102.8. The HSQC and HMBC for 5-amino-2,4-dichlorophenol are presented in Supplementary Figs. 54 and 55, respectively.

Analysis of 2,4-dichloro-1,5-diaminobenzene (2,4-diCl-**18**): ^1^H NMR (500 MHz, CD_3_OD, Supplementary Fig. 56) δ 7.04 (s, 2H); ^13^C NMR (100 MHz, CD_3_OD, Supplementary Fig. 57) δ 142.8, 128.3, 128.2, 108.5.

Analysis of 4-chloro-resveratrol (4-Cl-**23**): ^1^H NMR (400 MHz, CD_3_OD, Supplementary Fig. 58) δ 7.33 (d, *J* = 8.6 Hz, 2H), 6.93 (d, *J* = 16.2 Hz, 1H), 6.75 (d, *J* = 16.2 Hz, 1H), 6.74 (d, *J* = 8.6 Hz, 2H), 6.57 (br s, 2H).

Analysis of resveratrol (**23**): ^1^H NMR (400 MHz, CD_3_OD, Supplementary Fig. 59) δ 7.33 (d, *J* = 8.6 Hz, 2H), 6.93 (d, *J* = 16.2 Hz, 1H), 6.77 (d, *J* = 16.6 Hz, 1H), 6.74 (d, *J* = 8.6 Hz, 2H), 6.42 (d, *J* = 2.2 Hz, 2H), 6.13 (t, *J* = 2.2 Hz, 1H).

### Crystallization of PltM

PltM crystals were obtained by the hanging drop method with drops containing 0.5 μL of PltM (40 mg/mL) and 0.5 μL of the reservoir solution (0.1 M Tris, pH 8, 0.2 M NaCl, 0.1 M CaCl_2_ and 12–17% PEG 8000). The drops were equilibrated against 0.5 mL of reservoir solution at 21 °C. Long rod-shaped crystals appeared after 1–3 days. The crystals were cryoprotected by a gradual transfer to the solution with the same composition as the reservoir solution, additionally containing 20% glycerol. The crystals were then frozen by a rapid immersion into liquid nitrogen.

### Determination of the crystal structure of PltM

PltM does not contain a sufficient number of Met residues for structure determination by using anomalous signal from selenium atoms in Se-Met PltM. However, PltM contains eight Cys residues, which, if accessible, would react with Hg salts. Hg derivative crystals of PltM were prepared by transferring native crystals from its mother liquor to the reservoir solution containing 1 mM ethyl mercury phosphate (EMP) and incubated overnight. These crystals were cryoprotected similarly to the native crystals. X-ray diffraction data for this and other crystals of PltM were collected at 100 K at the wavelength of 1 Å at synchrotron beamline 22-ID at the Advanced Photon Source at the Argonne National Laboratory (Argonne, IL). All datasets were indexed, integrated and scaled using HKL2000^33^. The structure was determined by the SAD method from the EMP derivative data set (using the wavelength of 1.0 Å), as follows. A heavy atom search by using direct method-based SHELXD program^34^ initially yielded a substructure of 22 Hg atoms in the asymmetric unit. This Hg substructure was used as an input in Autosolve in PHENIX suite to obtain initial phases^35^, which were bootstrapped by difference Fourier analysis to yield the total of 33 Hg atoms and a readily interpretable electron density map, with the figure of merit of 0.71 after density modification. The structure of the Hg-derivatized PltM was then iteratively built by using COOT^36^ and refined by using REFMAC5^37^ (Supplementary Table 4). The refined structure contained four monomers of PltM and 33 Hg atoms coordinated to Cys residues per asymmetric unit. A monomer of PltM from this structure was then used as a search model to determine the structure of native PltM by molecular replacement with Phaser^38^ in CCP4i suite^39^. The native crystal structure of PltM was then iteratively adjusted and refined by using COOT^36^ and REFMAC5^37^, respectively. Supplementary Table 4 contains data collection and structure refinement statistics for this and other crystal structures in this study. The crystal structure coordinates and structure factor amplitudes for all crystal structures were deposited in the Protein Data Bank under accession codes specified in Supplementary Tables 4 and 5.

### Structure determination for the PltM–FAD intermediate

PltM crystals were soaked in the reservoir solution used to obtained native PltM crystals, with additional 0.5 mM of FAD. The crystals were then gradually transferred to the reservoir solution with 20% v/v PEG 400 and 0.5 mM FAD, prior to quick immersion in liquid nitrogen. The diffraction data were collected and processed as described above. Rigid body refinement followed by restrained refinement were performed starting from the structure of apo PltM. FAD was readily discernable in the omit *F*_o_–*F*_c_ map. Refinement and model building was carried out as described above.

### Structure determination for the holo PltM–FAD complex

Wild-type PltM and the L111Y mutant (each at 40 mg/mL) were crystallized by using the reservoir solution composed of 0.1 M Tris, pH 8, 0.2 M NaBr, 0.1 M CaCl_2_, and 14% PEG 8000 (10% PEG 8000 in case of the PltM L111Y mutant). The crystals were gradually transferred to the cryoprotectant solution (0.1 M Tris, pH 8, 0.2 M NaBr, 1 mM FAD, 16% PEG 8000 (14% PEG 8000 for the PltM L111Y mutant), 20% PEG 400 and 1 mM FAD) and incubated overnight. Prior to rapid freezing via liquid nitrogen, crystals were briefly transferred to the cryoprotectant solution containing additionally 0.2 M sodium dithionite. The crystal structures were determined by a procedure analogous to that described above.

### Structure determination for PltM–FAD–phloroglucinol complex

Native crystals of PltM were transferred to reservoir solution with 0.5 mM FAD either without or with 1 mM of phloroglucinol for 10 min, then to the cryoprotectant with the same composition, additionally containing 20% v/v PEG 400. After an overnight incubation, the crystals were rapidly frozen in liquid nitrogen. We tested compounds **1**, **2**, **3**, **8**, **21**, **23**, and **24**. Data collection, processing, and structure determination were carried out as described above. FAD was clearly discernable in the omit *F*_o_–*F*_c_ electron density map. Out of all substrates tested, only compound **1** (phloroglucinol) yielded omit *F*_o_–*F*_c_ electron density. Phloroglucinol was built into a very strong and featureful polder omit *mF*_o_–*DF*_c_ electron density^25^ in three out of four substrate binding sites in the asymmetric unit (Fig. 4b in the main text).

## Supplementary information

Supplementary Information

Reporting Summary

## Data Availability

The crystal structure coordinates and structure factor amplitudes for all crystal structures were deposited in the Protein Data Bank under accession codes 6BZN, 6BZI, 6BZA, 6BZQ, 6BZT and 6BZZ, as described in Supplementary Tables 4 and 5. NMR spectra, LC-MS, and other chromatographic data are included in the raw format in Supplementary Information. Other data are available from the corresponding authors upon reasonable request.
