# Publisher Correction: Unusual substrate and halide versatility of phenolic halogenase PltM

**DOI:** 10.1038/s41467-019-09731-8

**Published:** 2019-04-30

**Authors:** Shogo Mori, Allan H. Pang, Nishad Thamban Chandrika, Sylvie Garneau-Tsodikova, Oleg V. Tsodikov

**Affiliations:** Department of Pharmaceutical Sciences, College of Pharmacy, University of Kentucky, Lexington, KY 40536-0596 USA

**Keywords:** Enzymes, X-ray crystallography

Correction to: *Nature Communications* 10.1038/s41467-019-09215-9, published online 19 March 2019.

The original version of this Article contained an error in Fig. 1, in which the labels ‘NADP^+^’ and ‘NADPH + H^+^’ were incorrectly given as ‘NADPH’ and ‘NADPH^+^ + H^+^’, respectively. This has been corrected in both the PDF and HTML versions of the Article.

