# Supplementary material for: Unusual substrate and halide versatility of phenolic halogenase PltM
Source: Nat Commun. 2019 Mar 19;10:1255. doi: 10.1038/s41467-019-09215-9 (PMC6424973; doi:10.1038/s41467-019-09215-9)
Supplement: Supplementary file 1 — Supplementary Information [file 41467_2019_9215_MOESM1_ESM.pdf]

Supplementary Information for manuscript “Unusual substrate and halide versatility of phenolic halogenase PltM” by Mori et al.

Supplementary Table 1. List of FAD-dependent halogenases with known crystal structure and their respective substrates.

Main substrate	Halogenase	Halogenation position	PDB codes*
Phloroglucinol (this study)	PltM	C2 or C2 and C4	6BZN (apo)<br>6BZA (phloroglucinol and partially bound FAD)<br>6BZQ (FAD)<br>6BZZ (FAD-partially bound)<br>6BZT (L111Y, FAD)
L-tryptophan	PyrH	C5	2WES (mutant E46Q, FAD)[1]<br>2WET (FAD and L-Trp)[1]<br>2WEU (L-Trp)[1]
	MibH	C5	5UAO (FAD)[2]
	SttH	C6	5HY5 (FAD)[3]
	PrnA	C7	2APG (FAD)[4]<br>2AQJ (FAD and L-Trp)[4]<br>2AR8 (FAD and 7-Cl-L-Trp)[4]<br>2JKC (mutant: E346D, FAD and L-Trp)[5]<br>4Z43 (mutant: E450K, FAD)[6]<br>4Z44 (mutant: E454K, FAD)[6]
	RebH	C7	2O9Z (apo)[7]<br>2OA1 (FAD and L-Trp)[7]<br>2OAL (FAD)[8]<br>2OAM (apo)[8]<br>2E4G (L-Trp)[8]
	Th-Hal	C5 and C6	5LV9 (apo)[9]
Premalbrancheamide	MalA'	C9 or C10	5WGR (FAD and premalbrancheamide)[10]<br>5WGS (mutant: H253F, FAD and premalbrancheamide)[10]<br>5WGT (mutant: H253A, FAD and premalbrancheamide)[10]<br>5WGU (mutant: E494D, FAD and premalbrancheamide)[10]<br>5WGV (mutant: C112S/C128S, FAD and premalbrancheamide)[10]<br>5WGW (FAD and malbrancheamide)[10]<br>5WGX (mutant: H253A, FAD and malbrancheamide)[10]<br>5WGY (mutant: C112S/C128S, FAD and malbrancheamide)[10]<br>5WGZ (FAD and malbrancheamide)[10]
Pyrrolyl-*S*-carrier protein	PltA	C4 and/or C5	5DBJ (FAD)[11]
	Mpy16	C4 and/or C5	5BUK (FAD)[12]
	Bmp2	C3 or C3 and C4 or C3, C4 and C5	5BUL (mutant: Y302S/F306V/A345W, FAD)[12]<br>5BVA (FAD)[12]
Tyrosyl-*S*-carrier protein	CndH	C3	3E1T (FAD)[13]
Unknown	CmlS	-	3I3L (FAD)[14]
	-	-	3NIX (FAD)

* Bound ligands are FAD, a substrate or a product. Apo refers to the protein not bound to FAD, substrate, or products.

Supplementary Table 2. LC/MS data for *Assay 1* and *Assay 2* against phloroglucinol (**1**)

Substrate	Product	Calcd. mass (Da)	Obs. mass [M-H]$^-$ (Da)	Obs. mass [M+2-H]$^-$ (Da)	Obs. mass [M+4-H]$^-$ (Da)	Retention time (min)	Assay #	Fig. #
**1**	**1**	126.0317	125.0244	-	-	32.306	Std	1b, S1, S8
	F-**1**	144.0223	-	-	-	-	1a	-
	diF-**1**	162.0129	-	-	-	-	1a	-
	Cl-**1**	159.9927	158.9851	160.9821	-	35.098	1b	1b, S1
	diCl-**1**	193.9537	192.9459	194.9428	196.9399	36.841	1b	1b, S1
	Br-**1**	203.9422	202.9345	204.9324	-	35.174	1c	1b, S1
	diBr-**1**	283.8527	-	-	-	-	1c	-
	I-**1**	251.9283	250.9207	-	-	35.866	1d	1b, S1
	diI-**1**	377.8250	376.8161	-	-	39.309	1d	1b, S1
**1**	Cl-**1**	159.9927	158.9887	160.9893	-	33.454	2a	1c, S2
	diCl-**1**	193.9537	-	-	-	-	2a	-
	Br-**1**	203.9422	202.9402	204.9382	-	33.932	2a	1c, S2
	diBr-**1**	283.8527	-	-	-	-	2a	-
	Cl,Br-**1**	237.9032	-	-	-	-	2a	-
	Cl-**1**	159.9927	158.9890	160.9853	-	33.029	2b	1c, S3
	diCl-**1**	193.9537	-	-	-	-	2b	-
	I-**1**	251.9283	250.9252	-	-	34.406	2b	1c, S3
	diI-**1**	377.8250	376.8221	-	-	38.055	2b	1c, S3
	Cl,I-**1**	285.8894	-	-	-	-	2b	-
	Br-**1**	203.9422	202.9412	204.9395	-	33.202	2c	1c, S4
	diBr-**1**	283.8527	-	-	-	-	2c	-
	I-**1**	251.9283	250.9296	-	-	34.121	2c	1c, S4
	diI-**1**	377.8250	376.8314	-	-	37.824	2c	1c, S4
	Br,I-**1**	329.8388	-	-	-	-	2c	-
	Cl-**1**	159.9927	158.9867	160.9834	-	32.423	2d	1d, S5
	diCl-**1**	193.9537	192.9484	194.9438	196.9387	34.490	2d	1d, S5
	I-**1**	251.9283	250.9217	-	-	33.960	2d	1d, S5
	diI-**1**	377.8250	376.8158	-	-	37.678	2d	1d, S5
	Cl,I-**1**	285.8894	284.8811	286.8795	-	36.027	2d	1d, S5
	Br-**1**	203.9422	202.9403	204.9384	-	32.647	2e	1d, S6
	diBr-**1**	283.8527	-	-	-	-	2e	-
	I-**1**	251.9283	250.9285	-	-	33.572	2e	1d, S6
	diI-**1**	377.8250	376.8273	-	-	37.406	2e	-
	Br,I-**1**	329.8388	-	-	-	-	2e	-

Note: Although we looked for trihalogenated **1**, we did not observe any. All masses were measured in negative mode.

Supplementary Table 3. LC/MS data for all tested substrates.

**Substrate**	**Product**	**Calcd. mass (Da)**	**Obs. mass $[M-H]^-$ /$[M+H]^+$ (Da)**	**Obs. mass $[M+2-H]^-$ /$[M+2+H]^+$ (Da)**	**Obs. mass $[M+4-H]^-$ /$[M+4+H]^+$ (Da)**	**Retention time (min)**	**Assay #**	**Fig. #**
**2**	**2**	94.0419	93.0361	-	-	33.710	Std	S8, S10
	Cl-**2**	128.0029	126.9964	-	-	41.664	1b	S10
	diCl-**2**	161.9639	-	-	-	-	1b	-
	I-**2**	219.9385	-	-	-	-	1d	-
	diI-**2**	345.8352	-	-	-	-	1d	-
**3**	**3**	110.0368	109.0305	-	-	33.240	Std	S8, S11
	Cl-**3**	143.9978	142.9902	144.9869	-	36.519	1b	S11
	diCl-**3**	177.9588	176.9506	178.9481	180.9499	39.638	1b	S11
	I-**3**	235.9334	234.9252	-	-	36.242/38.268	1d	S11
	diI-**3**	361.8301	360.8203	-	-	43.131	1d	S11
**4**	**4**	126.0317	125.0236	-	-	30.894	Std	S8, S12
	Cl-**4**	159.9927	158.9839	160.9824	-	34.591	1b	S12
	diCl-**4**	193.9537	-	-	-	-	1b	-
	I-**4**	251.9283	250.9216	-	-	36.852	1d	S12
	diI-**4**	377.8250	-	-	-	-	1d	-
**5**	**5**	126.0317	125.0238	-	-	28.929/34.051	Std	S8, S13
	Cl-**5**	159.9927	-	-	-	-	1b	-
	diCl-**5**	193.9537	-	-	-	-	1b	-
	I-**5**	251.9283	250.9216	-	-	33.935	1d	S13
	diI-**5**	377.8250	-	-	-	-	1d	-
**6**	**6**	124.0524	123.0513	-	-	33.483	Std	S8, S14
	Cl-**6**	158.0135	157.0130	159.0110	-	40.546	1b	S14
	diCl-**6**	191.9745	-	-	-	-	1b	-
	I-**6**	249.9491	-	-	-	-	1d	-
	diI-**6**	375.8457	-	-	-	-	1d	-
**7**	**7**	154.0630	153.0581	-	-	38.317	Std	S8, S15
	Cl-**7**	188.0240	187.0184	189.0151	-	39.581	1b	S15
	diCl-**7**	221.9850	-	-	-	-	1b	-
	I-**7**	279.9596	278.9604	-	-	41.404	1d	S15
	diI-**7**	405.8563	-	-	-	-	1d	-
**8**	**8**	143.9978	142.9949	144.9918	-	34.503	Std	S8, S16
	Cl-**8**	177.9588	176.9571	178.9542	180.9509	37.481	1b	S16
	I-**8**	269.8945	268.8947	270.8920	-	39.385	1b	S16
**9**	**9**	124.0524	123.0522	-	-	34.559	Std	S8, S17
	Cl-**9**	158.0135	157.0140	159.0111	-	37.561	1b	S17
	diCl-**9**	191.9754	190.9761	192.9726	194.9695	40.863	1b	S17
	I-**9**	249.9491	248.9543	-	-	37.418/39.341	1d	S17
	diI-**9**	375.8457	374.8504	-	-	42.605	1d	S17
**10**	**10**	182.0579	181.0577	-	-	33.725/33.985	Std	S8, S18
	Cl-**10**	216.0189	215.0194	217.0166	-	36.019	1b	S18
	diCl-**10**	249.9800	-	-	-	-	1b	-
	I-**10**	307.9546	306.9588	-	-	36.538/37.232	1d	S18
	diI-**10**	433.8512	-	-	-	-	1d	-
**11**	**11**	140.0473	139.0396	-	-	29.450/29.633	Std	S8, S19
	F-**11**	158.0379	-	-	-	-	1a	-
	diF-**11**	176.0285	-	-	-	-	1a	-
	Cl-**11**	174.0084	173.0000	174.9968		32.078	1b	S19
	diCl-**11**	207.9694	206.9603	208.9571	210.9538	33.235	1b	S19
	Br-**11**	217.9579	216.9563	218.9548	-	31.923/32.405	1c	S19
	diBr-**11**	295.8684	-	-	-	-	1c	-
	I-**11**	265.9440	264.9321	-	-	33.187/33.529	1d	S19

	diI-**11**	391.8406	-	-	-	-	1d	-
**12**	**12**	138.0317	137.0243	-	-	33.265/33.518	Std	S8, S20
	Cl-**12**	171.9927	170.9844	172.9814	-	36.113	1b	S20
	diCl-**12**	205.9537	204.9444	206.9408	208.9364	37.796	1b	S20
	I-**12**	263.9283	262.9201	-	-	36.208/37.551	1d	S20
	diI-**12**	389.8250	-	-	-	-	1d	-
**13**	**13**	152.0473	151.0391	-	-	33.227/33.518	Std	S8, S21
	Cl-**13**	186.0084	184.9992	186.9964	-	35.651	1b	S21
	diCl-**13**	219.9694	-	-	-	-	1b	-
	I-**13**	277.9440	276.9325	-	-	36.198/36.740/36.980	1d	S21
	diI-**13**	403.8406	-	-	-	-	1d	-
**14**	**14**	154.0266	153.0176	-	-	33.616	Std	S8, S22
	Cl-**14**	187.9876	-	-	-	-	1b	-
	diCl-**14**	221.9487	-	-	-	-	1b	-
	I-**14**	279.9233	278.9143	-	-	37.380	1d	S22
	diI-**14**	405.8199	-	-	-	-	1d	-
**15**	**15**	168.0423	167.0416	-	-	35.985	Std	S8, S23
	Cl-**15**	202.0033	200.9954	202.9931	-	38.057	1b	S23
	diCl-**15**	235.9643	-	-	-	-	1b	-
	I-**15**	293.9389	292.9421	-	-	39.295	1d	S23
	diI-**15**	419.8355	-	-	-	-	1d	-
**16**	**16**	109.0528	108.0491	-	-	31.116	Std	S9, S24
	Cl-**16**	143.0138	142.0117	144.0086	-	35.795/37.079	1b	S24
	diCl-**16**	176.9748	175.9735	177.9707	179.9676	40.487	1b	S24
	I-**16**	234.9494	233.9476	-	-	37.974/38.929	1d	S24
	diI-**16**	360.8460	359.8479	-	-	44.288	1d	S24
**17** [a]	**17**	153.0790	154.0863	-	-	33.634	Std	S9, S25
	Cl-**17**	187.0400	188.0466	190.0432	-	38.621	1b	S25
	diCl-**17**	211.0010	-	-	-	-	1b	-
	I-**17**	278.9756	279.9806	-	-	44.046	1d	S25
	diI-**17**	404.8723	-	-	-	-	1d	-
**18**	**18**	108.0687	107.0669	-	-	30.101	Std	S9, S26
	Cl-**18**	142.0298	141.0268	143.0239	-	36.581	1b	S26
	diCl-**18**	175.9908	-	-	-	-	1b	-
	I-**18**	233.9654	-	-	-	-	1d	-
	diI-**18**	359.8620	-	-	-	-	1d	-
**19**	**19**	212.0069	211.0327			35.780	Std	S9
	Cl-**19**	245.9680	-			-	1b	-
	diCl-**19**	279.9290	-			-	1b	-
	I-**19**	337.9036	-			-	1d	-
	diI-**19**	463.8002	-			-	1d	-
**20**	**20**	204.0899	203.0795			29.860	Std	S9
	Cl-**20**	238.0509	-			-	1b	-
	diCl-**20**	272.0119	-			-	1b	-
	I- **20**	329.9865	-			-	1d	-
	diI- **20**	455.8832	-			-	1d	-
**21**	**21**	225.1365	224.1264	-	-	27.654	Std	S9, S27
	Cl-**21**	259.0975	-	-	-	-	1b	-
	diCl-**21**	293.0585	-	-	-	-	1b	-
	I-**21**	351.0331	350.0230	-	-	28.222/28.485	1d	S27
	diI-**21**	476.9298	475.9219	-	-	29.804	1d	S27
**22** [a]	**22**	303.1471	304.1543	-	-	30.691/34.162	Std	S9, S28
	Cl-**22**	337.1081	-	-	-		1b	-
	diCl-**22**	371.0691	-	-	-		1b	-
	I-**22**	429.0437	430.0494	-	-	32.154	1d	S28
	diI-**22**	554.9403	-	-	-	-	1d	-
**23**	**23**	228.0786	227.0794	-	-	35.871/36.280/37.368	Std	S9, S29

	Cl-**23**	262.0397	261.0418	263.0390	-	37.565/38.532	1b	S29
	diCl-**23**	296.0007	-	-	-	-	1b	-
	I-**23**	353.9753	352.9816	-	-	38.531	1d	S29
	diI-**23**	479.8719	-	-	-	-	1d	-
**24**	**24**	290.0790	289.0838	-	-	31.263/31.372/31.712	Std	S9, S30
	Cl-**24**	324.0401	323.0450	325.0415	-	33.137	1b	S30
	diCl-**24**	358.0011	-	-	-	-	1b	-
	I-**24**	415.9757	414.9837	-	-	33.533/34.204	1d	S30
	diI-**24**	541.8723	-	-	-	-	1d	-

Note: Although we looked for halogenation beyond two sites, we did not observe any for the substrates tested. [a] All compounds were measured in negative mode except for 3,5-dimethoxyaniline (**17**) and fenoterol (**22**)

Supplementary Table 4. X-ray diffraction data collection and structure refinement statistics for apo-PltM and apo-PltM-Hg

	PltM	PltM-Hg
PDB ID	6BZN	6BZI
Data collection		
Space group	$P2_12_12_1$	$P2_12_12_1$
Number of monomers per asymmetric unit	4	4
Unit cell dimensions		
a, b, c (Å)	64.2, 157.1, 214.0	63.7, 156.3, 216.1
α, β, γ (°)	90, 90, 90	90, 90, 90
Resolution (Å)	39.88-1.80 (1.83-1.80)[a]	50.0-2.4 (2.44-2.40)[a]
R_{merge}	0.125 (0.766)	0.164 (0.708)
$I/\sigma I$	14.5 (1.7)[a]	11.2 (2.4)[a]
Completeness (%)	99.5 (96.3)	96.3 (92.2)
Redundancy	6.7 (6.1)	6.7 (6.5)
Structure refinement statistics		
Resolution (Å)	39.88-1.80	45.0-2.4
Number of unique reflections	189997	78214
R_{work} / R_{free}	0.161 / 0.189	0.209 / 0.256
No. of atoms		
Protein	15852	15595
Ligand/Ion	67	71
Water	2015	451
B-factors		
Protein	19.6	28.0
Ligand/Ion	31.4	27.3
Water	31.3	24.6
R.m.s. deviations		
Bond lengths (Å)	0.02	0.008
Bond angles (°)	1.71	1.19
Ramachandran plot statistics[b]		
% of residues in favored region	98.1	98.3
% of residues in allowed region	1.9	1.7
% of residues in outlier region	0	0
Ligands/Ions	Glycerol (10)[c]<br>Calcium (7)	Glycerol (3)<br>Calcium (4)<br>Mercury (25)<br>Ethylmercury (8)

[a] Numbers in parentheses indicate the values in the highest-resolution shell.
[b] Indicates Rampage statistics.[15]
[c] Number of ligands in the asymmetric unit.

Supplementary Table 5. X-ray diffraction data collection and structure refinement statistics for PltM-FAD-phloroglucinol, PltM-FAD, PltM L111Y-FAD and PltM-FAD intermediate complexes.

	PltM-FAD-phloroglucinol	PltM-FAD	PltM L111Y-FAD	PltM- FAD partially bound
PDB ID	6BZA	6BZQ	6BZT	6BZZ
Data collection				
Space group	$P2_12_12_1$	$P2_12_12_1$	$P2_12_12_1$	$P2_12_12_1$
Number of monomers per asymmetric unit	4	4	4	4
Unit cell dimensions				
a, b, c (Å)	64.2, 157.0, 213.7	63.3, 157.7, 213.5	64.0, 157.5, 213.0	63.82, 157.2, 214.0
α, β, γ (°)	90, 90, 90	90, 90, 90	90, 90, 90	90, 90, 90
Resolution (Å)	49.71-2.60 (2.64-2.60)[a]	35.00-2.75 (2.81-2.75)[a]	50.00-2.10 (2.14-2.10)[a]	49.55-2.05 (2.09-2.05)[a]
R_{merge}	0.172 (0.663)	0.15 (0.82)	0.197 (0.989)	0.175 (0.805)
$I/\sigma I$	14.0 (2.2)[a]	12.0 (2.0)[a]	10.7 (1.9)[a]	18.1 (3.0)[a]
Completeness (%)	98.9 (99.5)	96.0 (97.5)	94.7 (92.6)	98.2 (93.9)
Redundancy	6.0 (6.0)	4.6 (4.6)	5.4 (4.3)	7.5 (7.1)
Structure refinement statistics				
Resolution (Å)	40.00-2.60	35.00-2.75	35.0-2.10	40.00-2.05
Number of unique reflections	62405	54483	113768	125848
R_{work} / R_{free}	0.203 / 0.253	0.230 / 0.261	0.207 / 0.244	0.219 / 0.245
No. of atoms				
Protein	15796	15796	15899	15801
Ligand/Ion	79	218	228	76
Water	104	164	943	455
B-factors				
Protein	47.1	35.4	24.9	21.7
Ligand/Ion	70.7	67.3	36.0	33.6
Water	35.5	25.6	27.7	20.5
R.m.s. deviations				
Bond lengths (Å)	0.007	0.007	0.007	0.007
Bond angles (°)	1.17	1.095	1.219	1.207
Ramachandran plot statistics[b]				
% of residues in favored region	97.8	98.0	98.0	98.4
% of residues in allowed region	2.2	2.0	2.0	1.6
% of residues in outlier region	0	0	0	0
Ligands/Ions	phloroglucinol (3)<br>FAD (2)<br>Chloride (2)	FAD (4)<br>Chloride (4)<br>Bromide (2)	FAD (4)<br>Chloride (5)<br>Bromide (9)<br>Calcium (2)	FAD (4)<br>Calcium (4)

[a] Numbers in parentheses indicate the values in the highest-resolution shell.
[b] Indicates Rampage statistics.[15]
[c] Number of ligands in the asymmetric unit.

Supplementary Table 6. LC/MS data for cell-based assays.

Enzyme	Product	Calcd. mass (Da)	Obs. mass $[M-H]^-$ (Da)	Obs. mass $[M+2-H]^-$ (Da)	Obs. mass $[M+4-H]^-$ (Da)	Retention time (min)	Assay #	Fig. #
PltM WT	**1**	126.0317	125.0243	-	-	29.369	Std	3, S39
	Cl-**1**	159.9927	158.9852	160.9821	-	32.416	3	3, S39
	diCl-**1**	193.9537	192.9457	194.9430	196.9391	34.565	3	3, S39
PltM K87A	**1**	126.0317	125.0249	-	-	29.171	Std	3, S39
	Cl-**1**	159.9927	-	-	-	-	3	-
	diCl-**1**	193.9537	-	-	-	-	3	-
PltM L111Y	**1**	126.0317	125.0246	-	-	29.372	Std	3, S39
	Cl-**1**	159.9927	158.9847	160.9824	-	32.432	3	3, S39
	diCl-**1**	193.9537	192.9453	194.9419	196.9396	34.558	3	3, S39
PltM S404Y	**1**	126.0317	125.0245	-	-	29.368	Std	3, S39
	Cl-**1**	159.9927	158.9847	160.9817	-	32.393	3	3, S39
	diCl-**1**	193.9537	-	-	-	-	3	-
PltA	**1**	126.0317	125.0244	-	-	29.456	Std	S39
	Cl-**1**	159.9927	-	-	-	-	3	-
	diCl-**1**	193.9537	-	-	-	-	3	-

Note: Although we looked for trihalogenated **1**, we did not observe any. All masses were measured in negative mode.

Supplementary Table 7. Overall yield of optimized chlorination reactions for different substrates of PltM.

Substrate	% overall conversion[a] (trial 1, 2)[b]	Substrate	% overall conversion[a] (trial 1, 2)[b]
**2**	57, 25	**12**	28, 26
**3**	100, 100	**13**	5, 3
**6**	1, 4	**15**	34, 7
**8**	100, 100	**16**	100, 100
**9**	97, 96	**18**	100, 100
**10**	3, 2	**23**	24, 20
**11**	100,100		

[a] % overall conversion is the sum of all chlorinated products.
[b] Yields of two independent reactions are reported.

Supplementary Fig. 1. LC/MS analysis for the mono- and dihalogenation of compound **1**. The top row shows chlorination, the middle row bromination, and the bottom row iodination. The left column shows XIC spectra for **1** (orange), mono-halogenated **1** (blue), and dihalogenated **1** (pink). The middle and right columns show the MS spectra for mono-halogenated **1** and dihalogenated **1**, respectively.

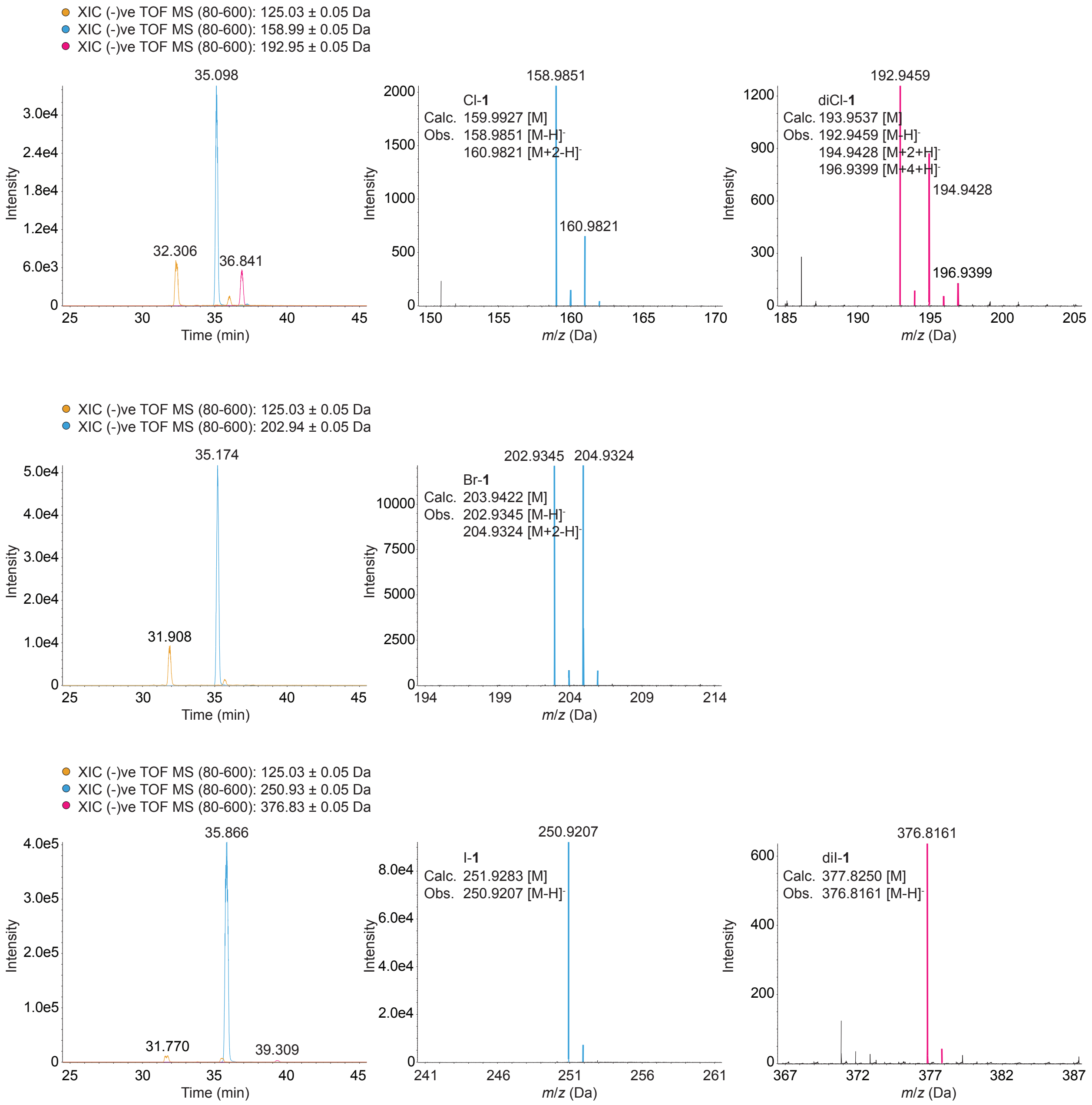

Supplementary Fig. 2. LC/MS analysis for the *assay 2a* (1:1 competition of Cl:Br). The left panel shows the XIC spectrum for **1** (orange), mono-chlorinated **1** (blue), and mono-brominated **1** (green). The middle and right panels show the MS spectra for mono-chlorinated **1** and mono-brominated **1**, respectively.

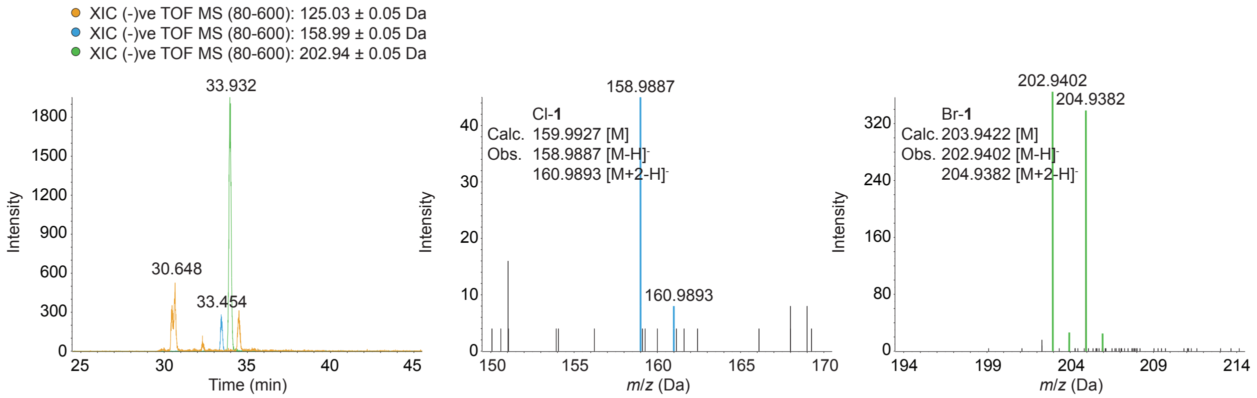

Supplementary Fig. 3. LC/MS analysis for the *assay 2b* (1:1 competition of Cl:I). The top left panel shows the XIC spectrum for **1** (orange), mono-chlorinated **1** (blue), mono-iodinated **1** (green), and diiodinated **1** (purple). The top middle, top right, and bottom left panels show the MS spectra for mono-chlorinated **1**, mono-iodinated **1**, and diiodinated **1**, respectively.

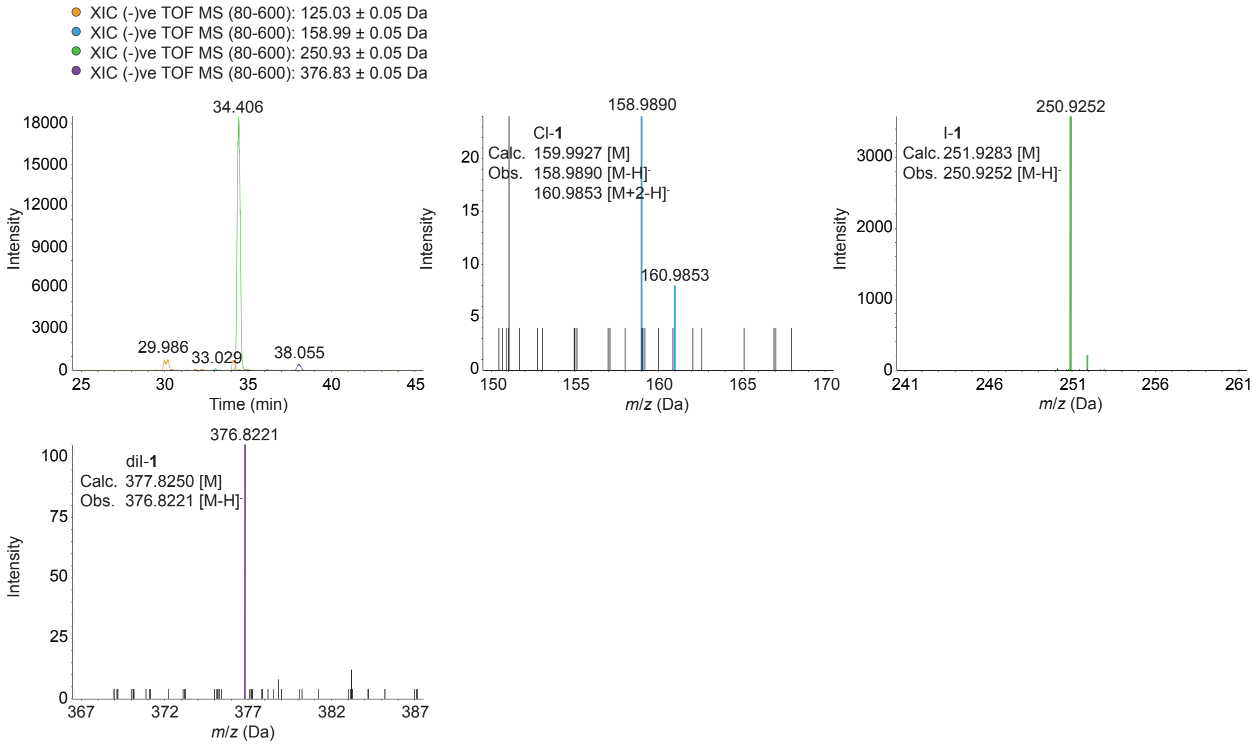

Supplementary Fig. 4. LC/MS analysis for the *assay 2c* (1:1 competition of Br:I). The top left panel shows the XIC spectrum for **1** (orange), mono-brominated **1** (blue), mono-iodinated **1** (green), and diiodinated **1** (purple). The top middle, top right, and bottom left panels show the MS spectra for mono-brominated **1**, mono-iodinated **1**, and diiodinated **1**, respectively.

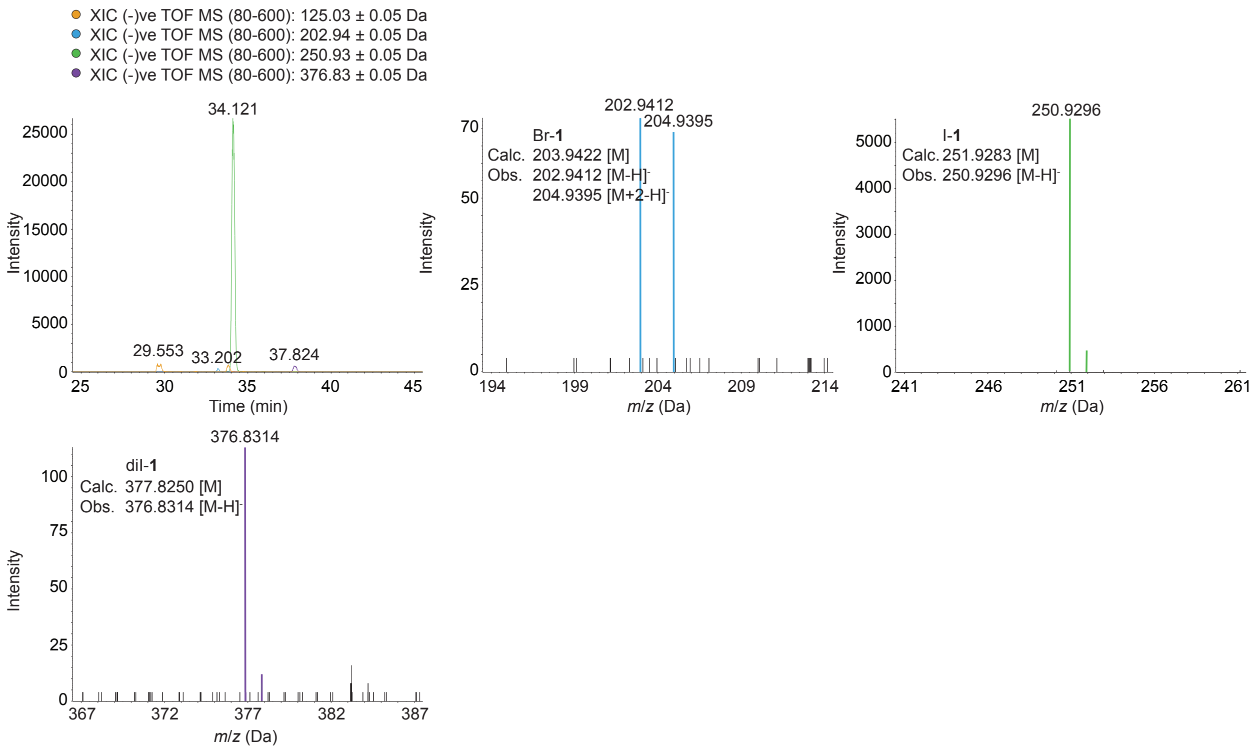

Supplementary Fig. 5. LC/MS analysis for the *assay 2d* (10:1 competition of Cl:I). The top left panel shows the XIC spectrum for **1** (orange), mono-chlorinated **1** (blue), dichlorinated **1** (pink), mono-iodinated **1** (green), diiodinated **1** (purple), and chloro-iodinated **1** (brown). Inset shows zoom-in of about 35 min. mark to show peak intensity for dichlorinated **1**. The top middle, top right, bottom left, bottom middle, and bottom right panels show the MS spectra for mono-chlorinated **1**, dichlorinated **1**, mono-iodinated **1**, diiodinated **1**, and chloro-iodinated **1**, respectively.

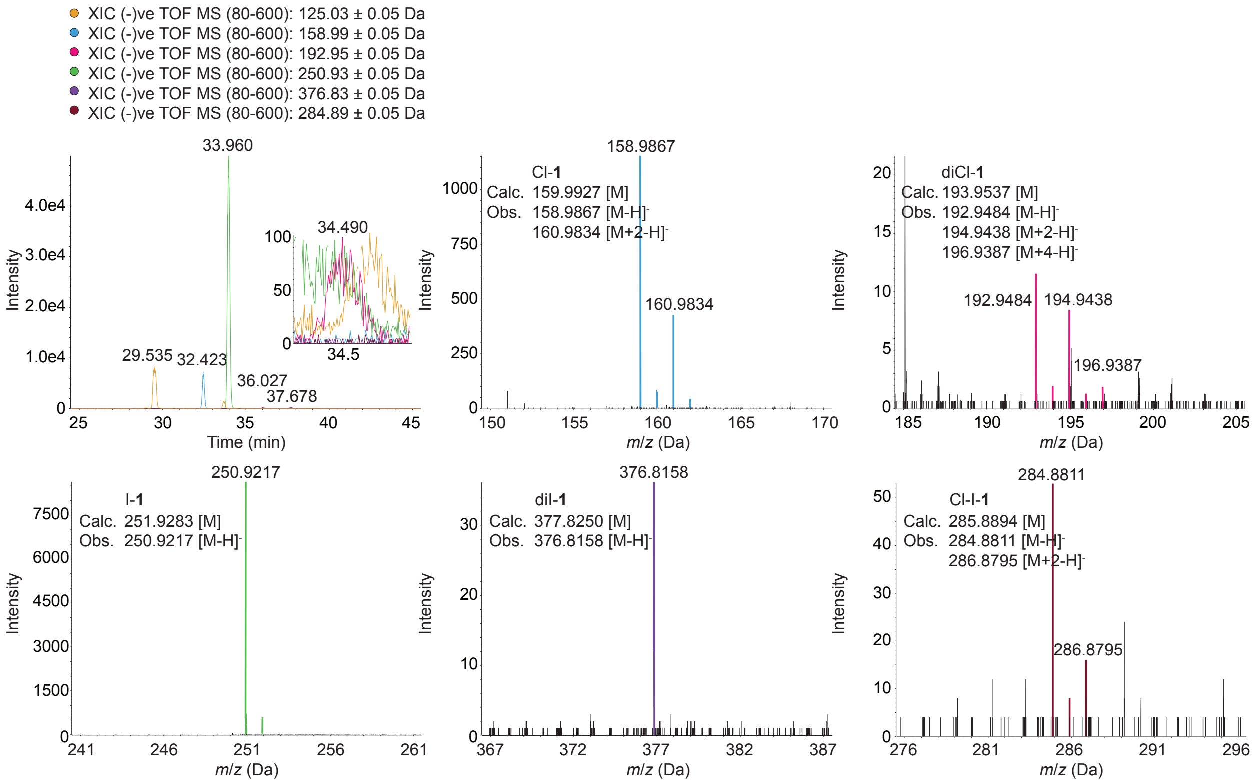

Supplementary Fig. 6. LC/MS analysis for the *assay 2e* (10:1 competition of Br:I). The top left panel shows the XIC spectrum for **1** (orange), mono-brominated **1** (blue), mono-iodinated **1** (green), and diiodinated **1** (purple). Inset shows zoom-in of about 37.5 min. mark to show peak intensity for diiodinated **1**. The top middle, top right, and bottom left panels show the MS spectra for mono-brominated **1**, mono-iodinated **1**, and diiodinated **1**, respectively.

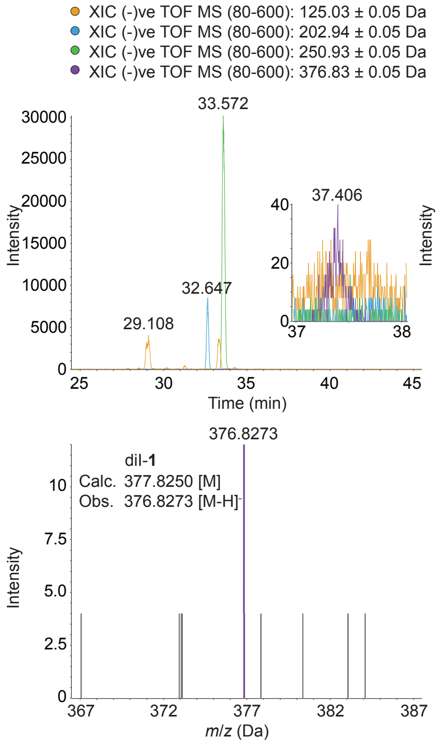

Supplementary Fig. 7. Compounds tested as potential substrates of PltM.

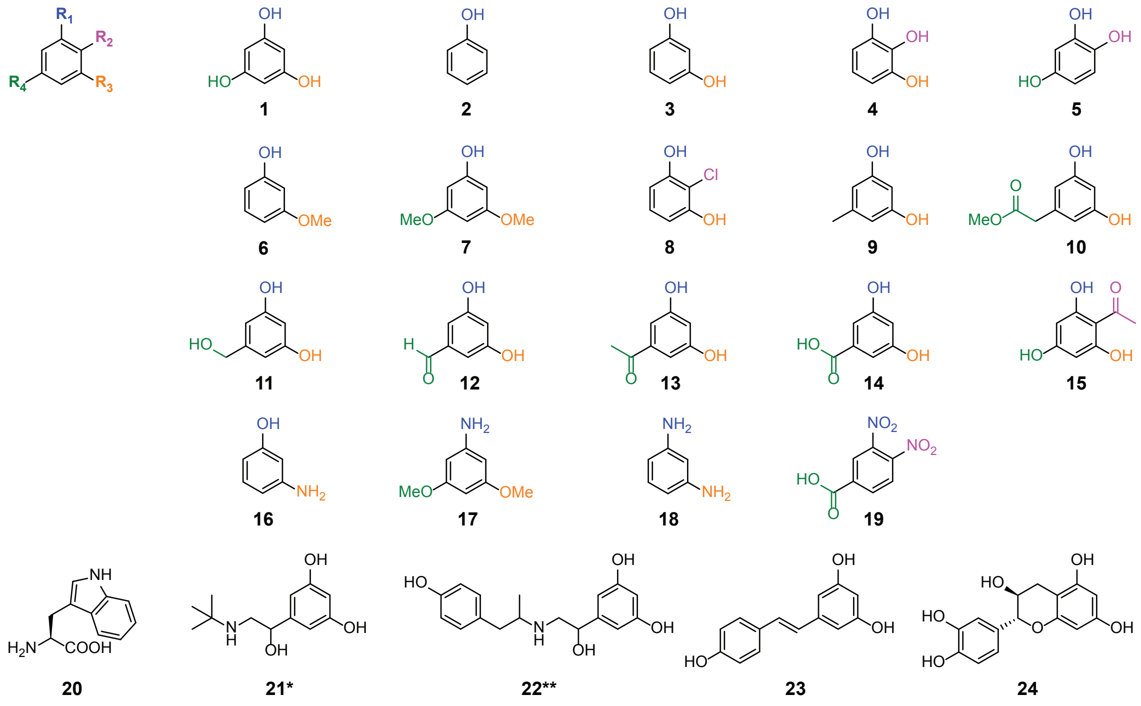

* Stereochemistry is not defined

** Racemic mixture of *R*,*R* and *S*,*S*

Supplementary Fig. 8. MS spectra for compounds **1-15** tested as potential substrates of PltM.

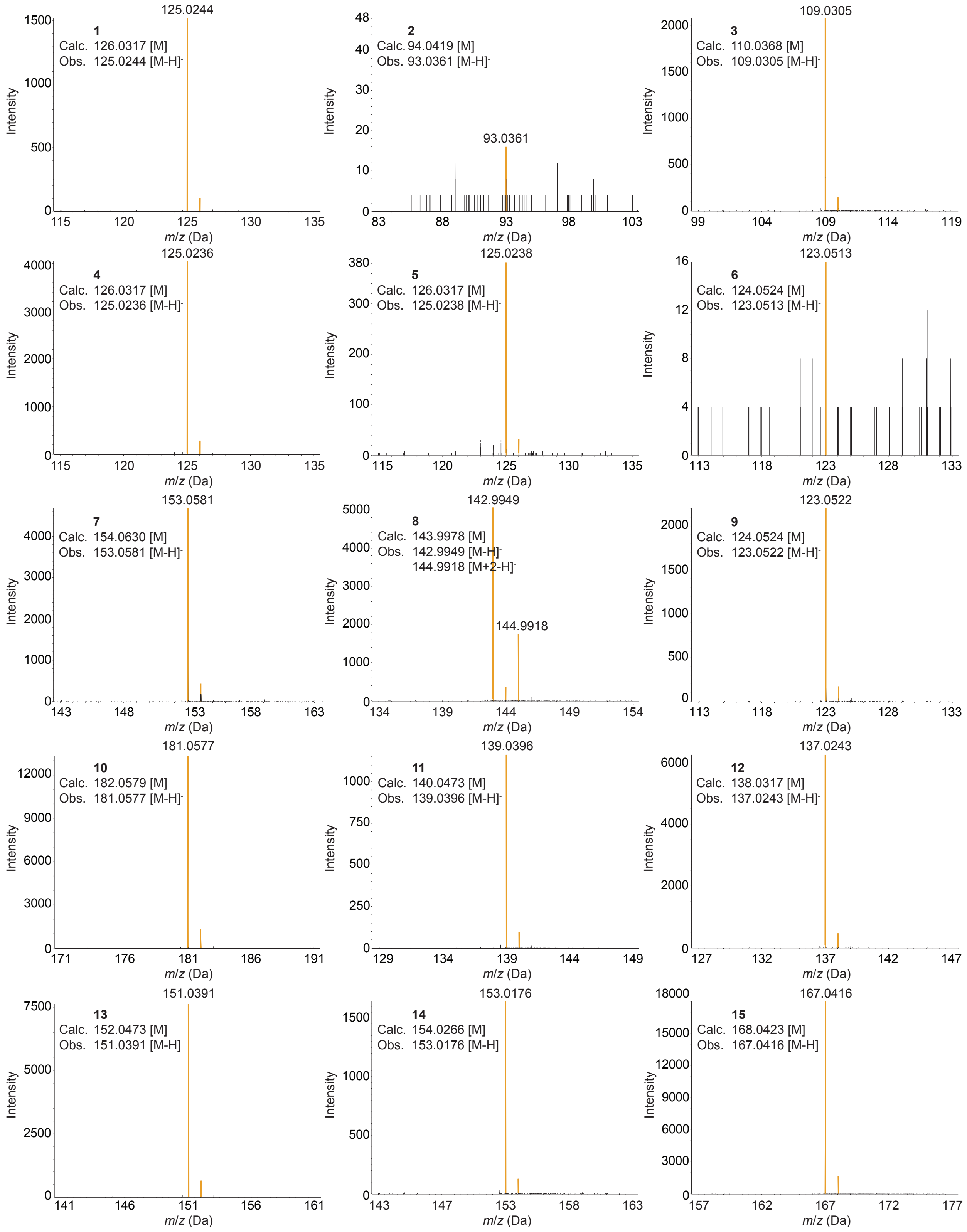

Supplementary Fig. 9. MS spectra for compounds **16-24** tested as potential substrates of PltM.

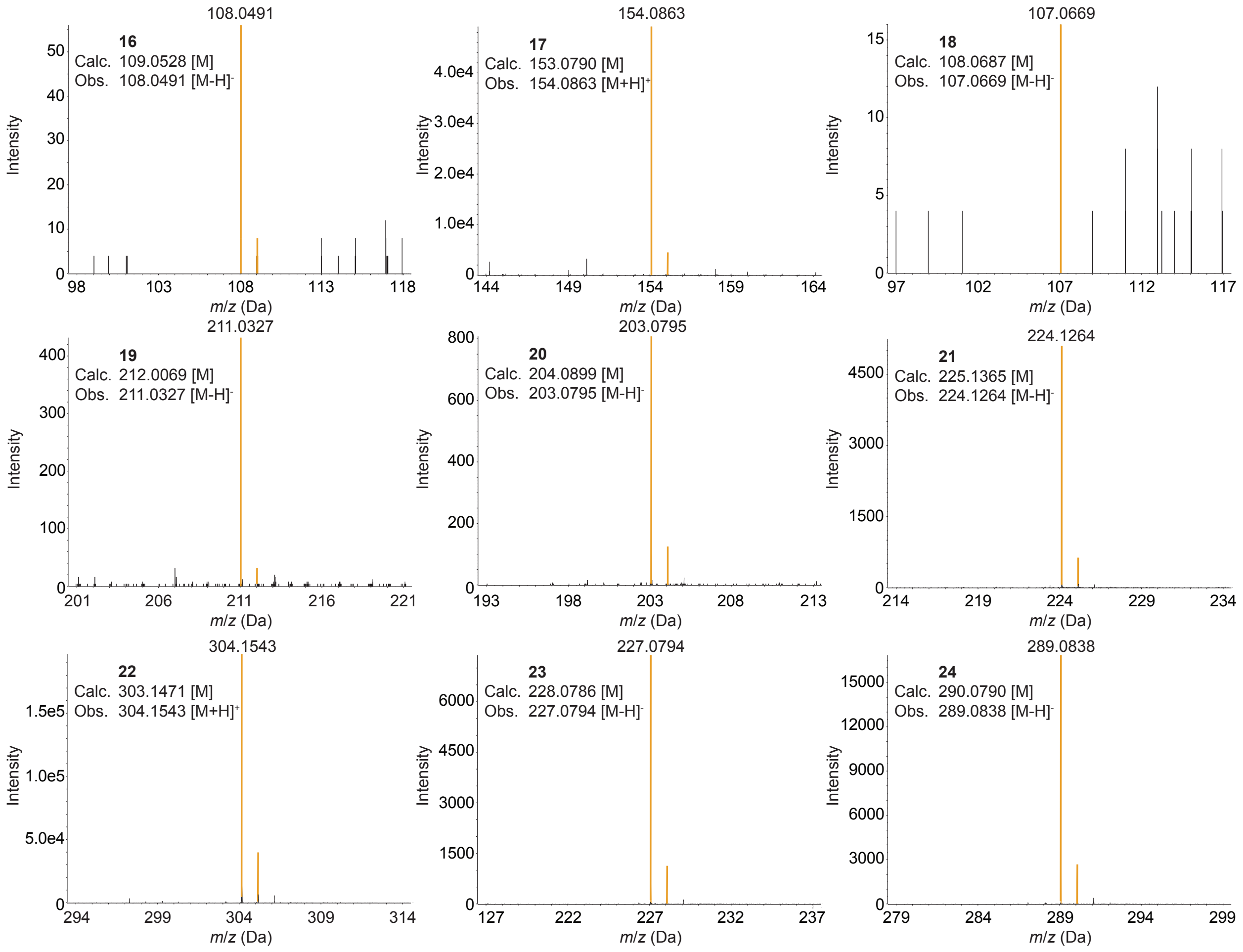

Supplementary Fig. 10. LC/MS analysis for the mono-chlorination of compound **2**. The left column shows XIC spectrum for **2** (orange) and mono-chlorinated **2** (blue). The right column shows the MS spectrum for mono-chlorinated **2**.

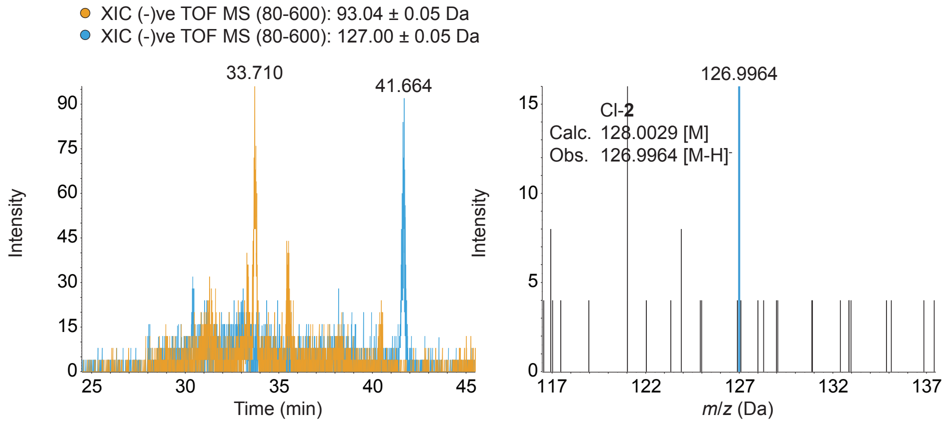

Supplementary Fig. 11. LC/MS analysis for the mono- and dihalogenation of compound **3**. The top row is for chlorination, and the bottom row is for iodination. The left column shows XIC spectra for **3** (orange), mono-halogenated **3** (blue), and dihalogenated **3** (pink). The middle and right columns show the MS spectra for mono-halogenated **3** and dihalogenated **3**, respectively. Inset shows zoom-in of the peak at 43.131 min for diiodinated **3**.

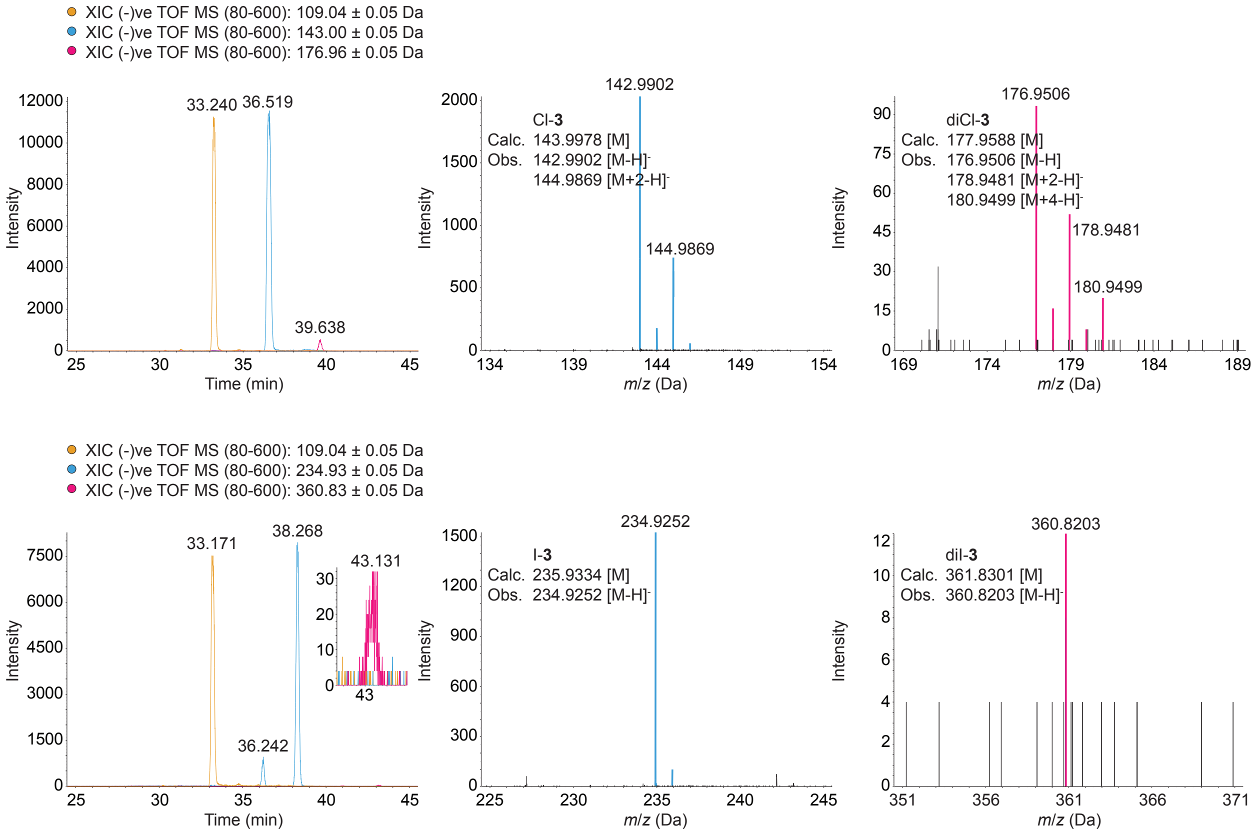

Supplementary Fig. 12. LC/MS analysis for the mono-halogenation of compound **4**. The top row is for chlorination, and the bottom row is for iodination. The left column shows XIC spectra for **4** (orange) and mono-halogenated **4** (blue). Inset shows zoom-in of the peak at 34.591 min mono-chlorinated **4**. The right column shows the MS spectra for mono-halogenated **4**.

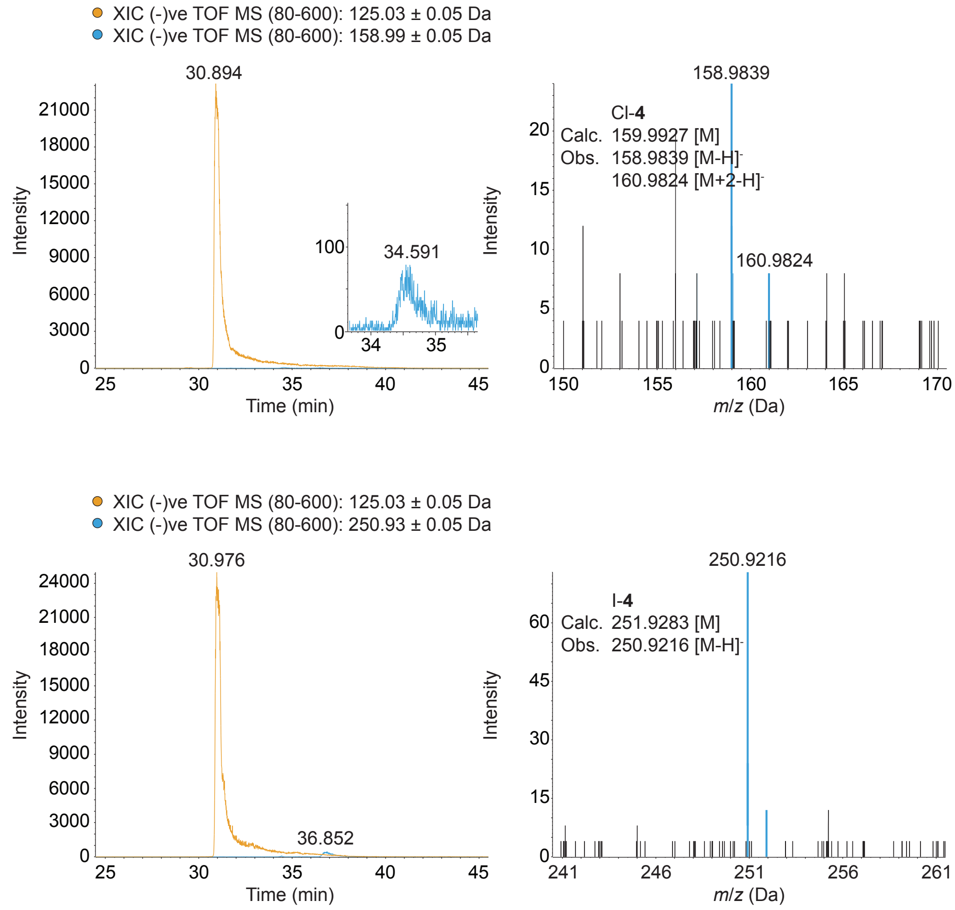

Supplementary Fig. 13. LC/MS analysis for the mono-iodination of compound **5**. The left column shows XIC spectrum for **5** (orange) and mono-iodinated **5** (blue). Inset shows zoom-in of the peak at 33.935 min for mono-iodinated **5**. The right column shows the MS spectrum for mono-iodinated **5**.

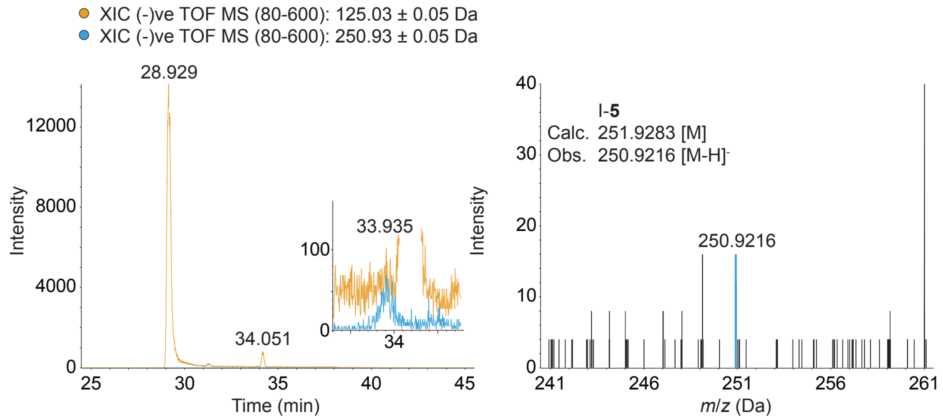

Supplementary Fig. 14. LC/MS analysis for the mono-chlorination of compound **6**. The left column shows XIC spectrum for **6** (orange) and mono-chlorinated **6** (blue). The right column shows the MS spectrum for mono-chlorinated **6**.

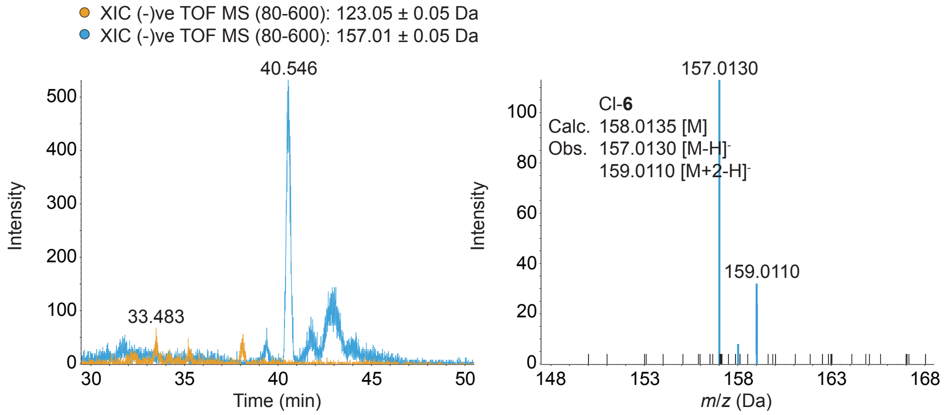

Supplementary Fig. 15. LC/MS analysis for the mono-halogenation of compound **7**. The top row is for chlorination, and the bottom row is for iodination. The left column shows XIC spectra for **7** (orange) and mono-halogenated **7** (blue). The right column shows the MS spectra for mono-halogenated **7**.

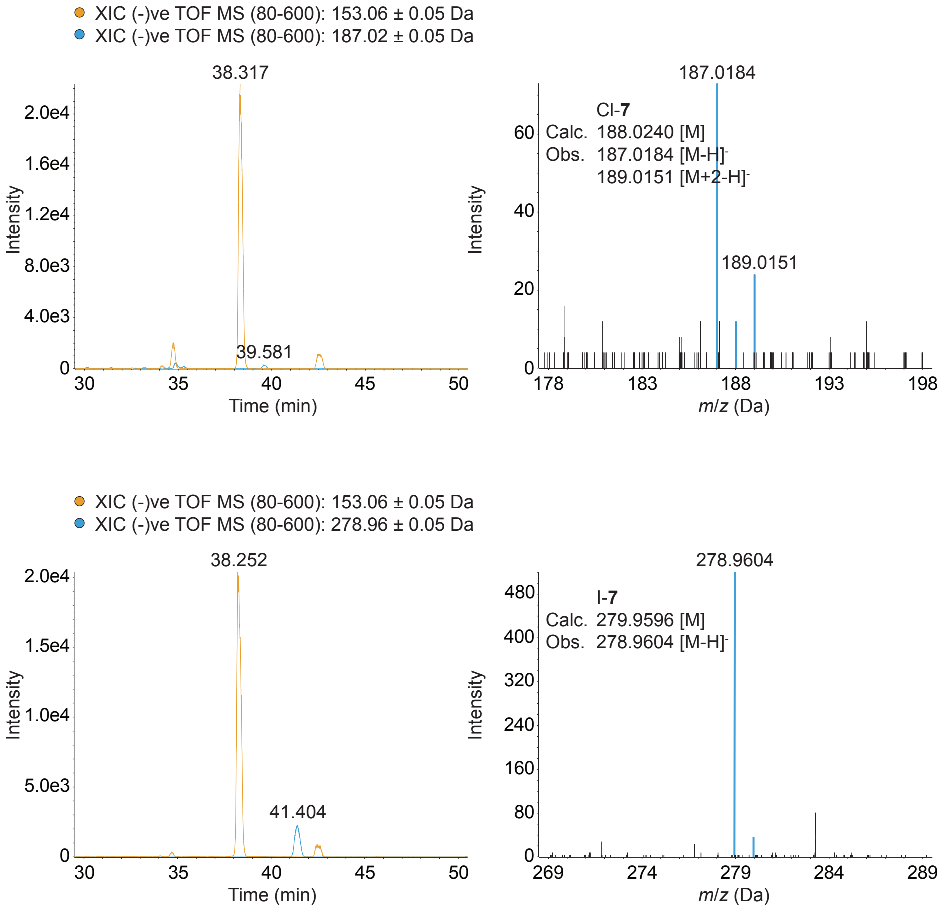

Supplementary Fig. 16. LC/MS analysis for the mono-halogenation of compound **8**. The top row is for chlorination, and the bottom row is for iodination. The left column shows XIC spectra for **8** (orange) and mono-halogenated **8** (blue). The right column shows the MS spectra for mono-halogenated **8**.

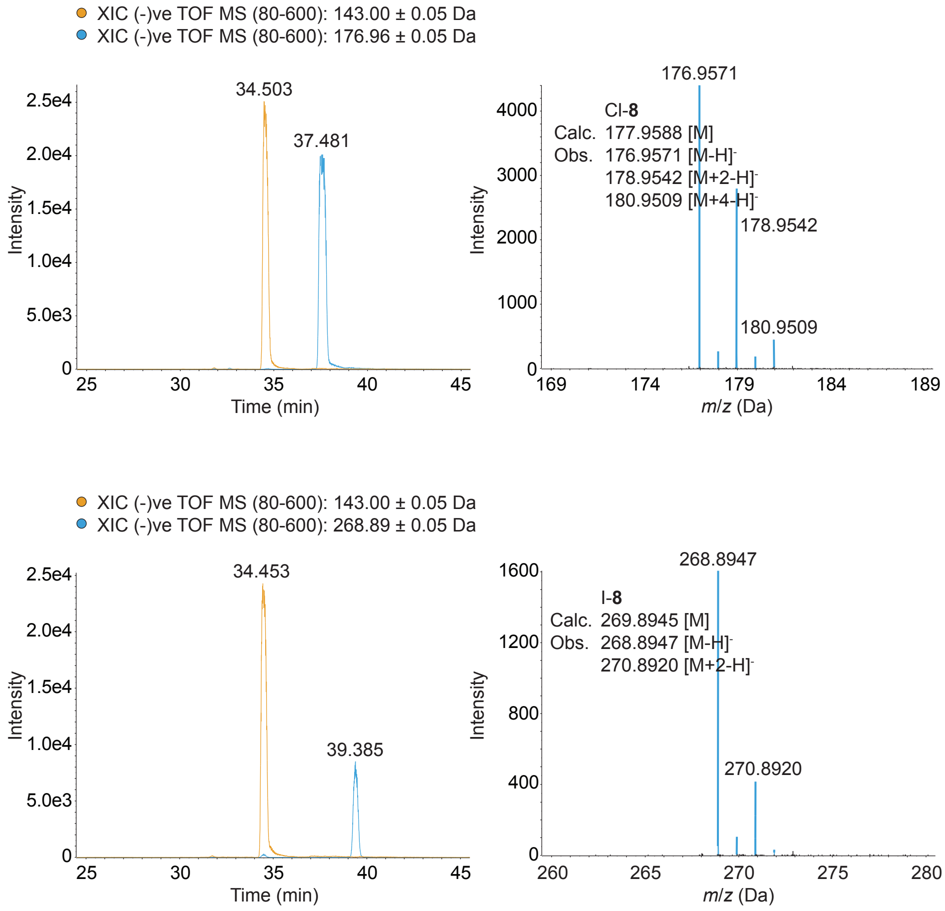

Supplementary Fig. 17. LC/MS analysis for the mono- and dihalogenation of compound **9**. The top row is for chlorination, and the bottom row is for iodination. The left column shows XIC spectra for **9** (orange), mono-halogenated **9** (blue), and dihalogenated **9** (pink). Inset shows zoom-in of the peak at 42.605 min for diiodinated **9**. The middle and right columns show the MS spectra for mono-halogenated **9** and dihalogenated **9**, respectively.

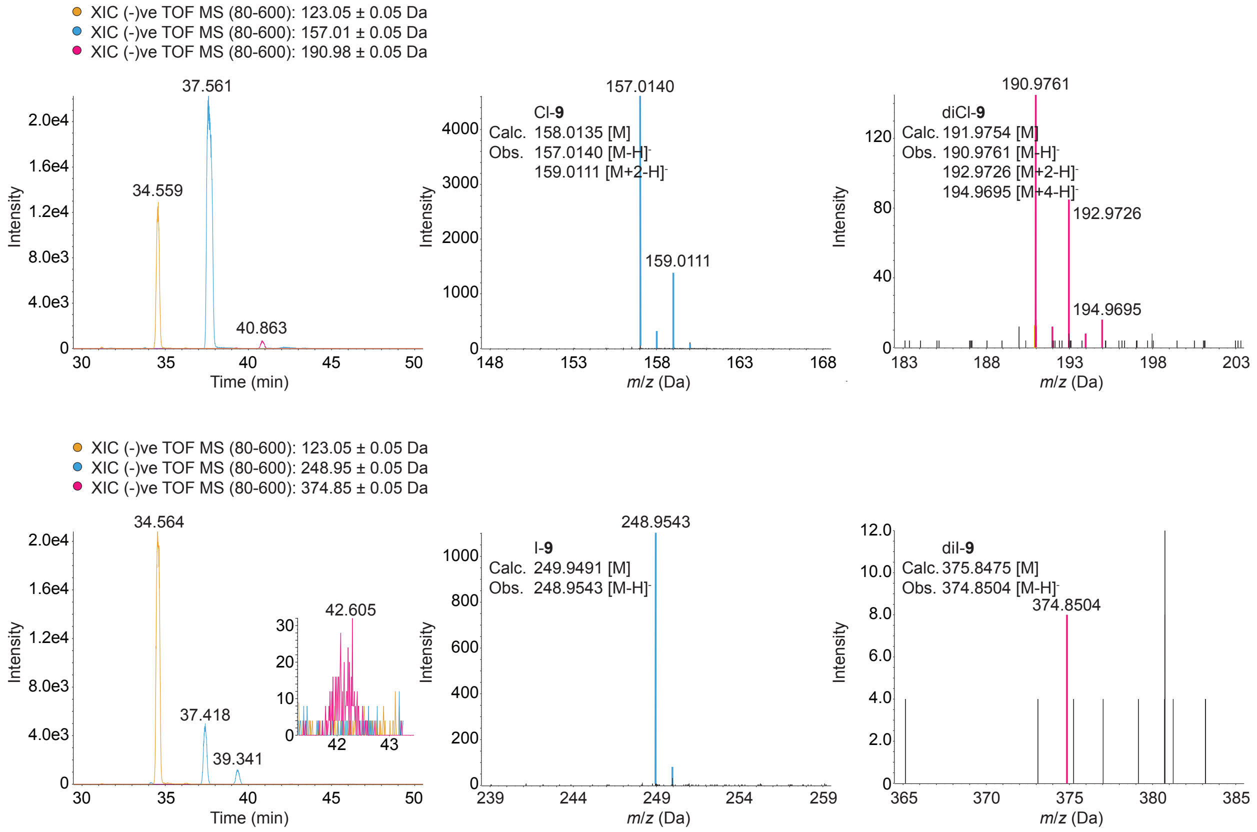

Supplementary Fig. 18. LC/MS analysis for the mono-halogenation of compound **10**. The top row is for chlorination, and the bottom row is for iodination. The left column shows XIC spectra for **10** (orange) and mono-halogenated **10** (blue). The right column shows the MS spectra for mono-halogenated **10**.

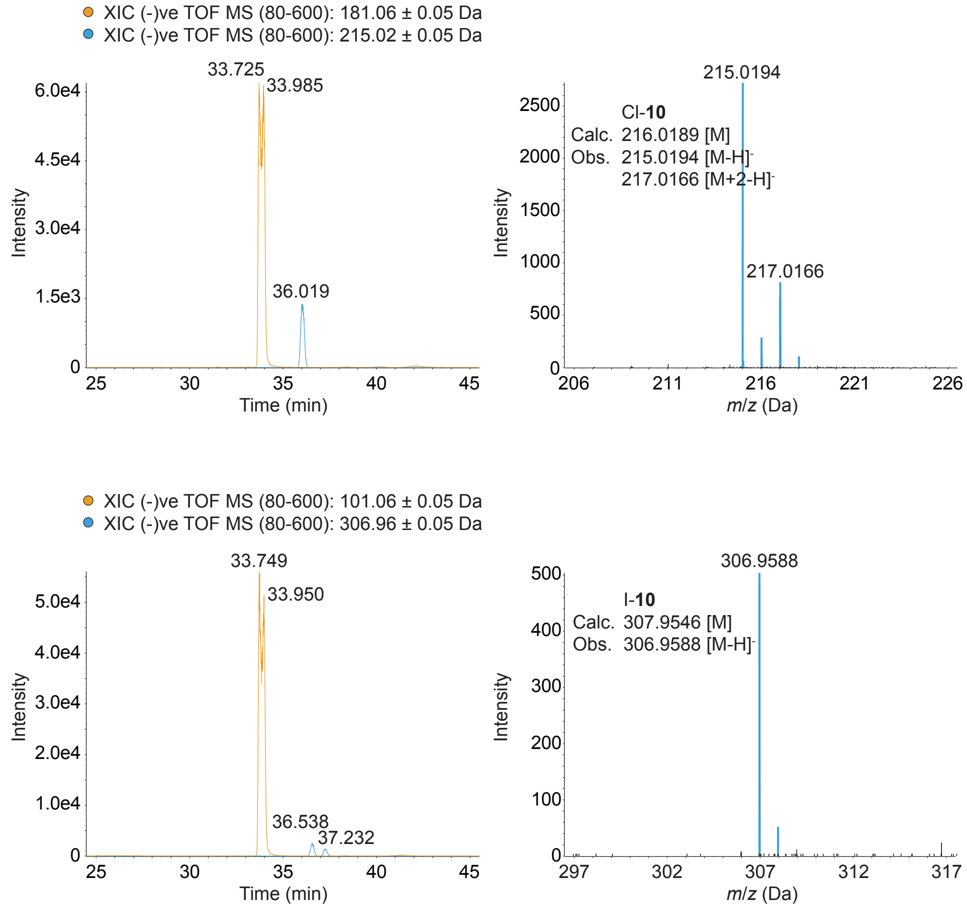

Supplementary Fig. 19. LC/MS analysis for the mono- and dihalogenation of compound **11**. The top row is for chlorination, the middle row is for bromination, and the bottom row is for iodination. The left column shows XIC spectra for **11** (orange), mono-halogenated **11** (blue), and dihalogenated **11** (pink). The middle and right columns show the MS spectra for mono-halogenated **11** and dihalogenated **11**, respectively.

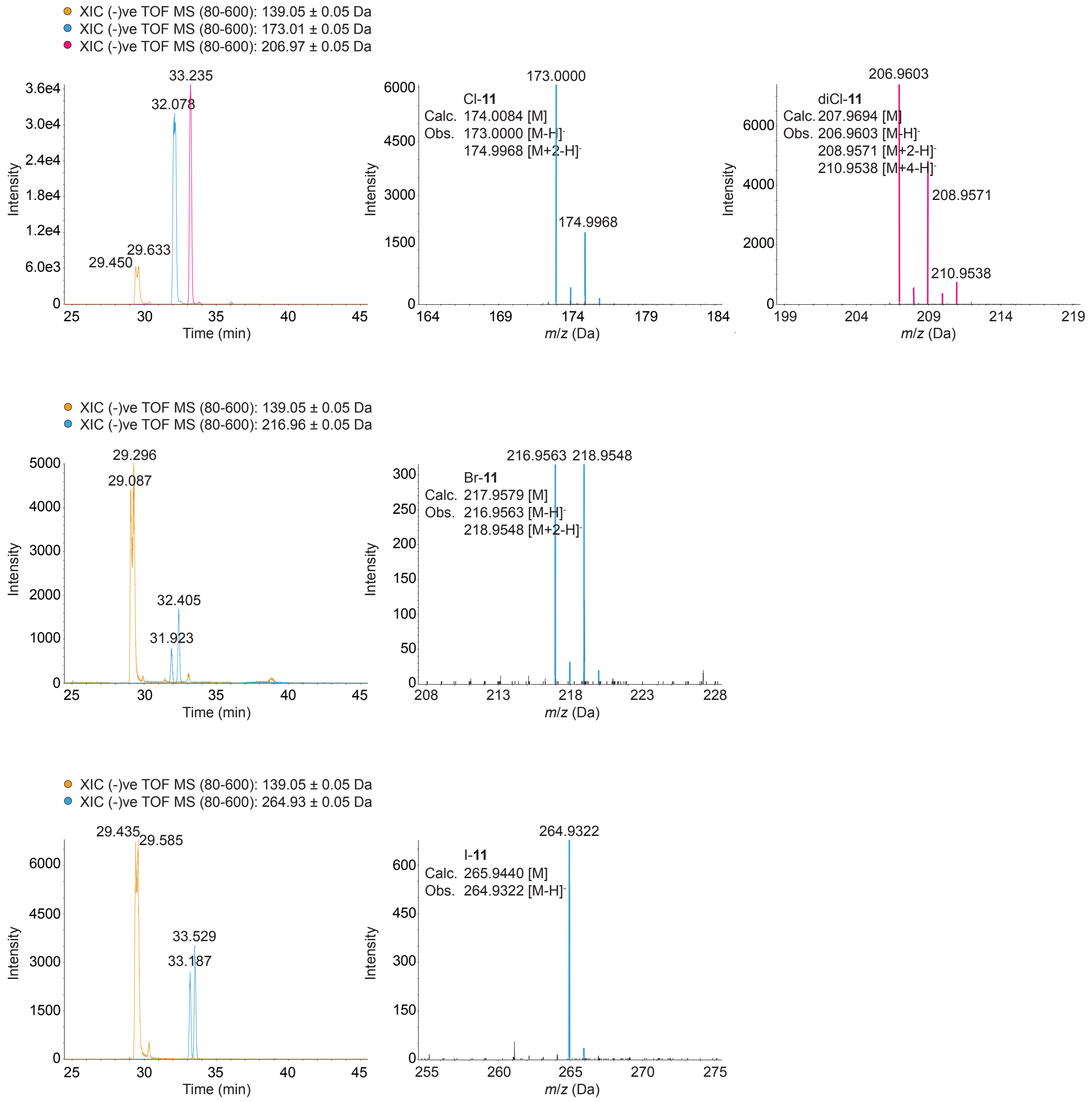

Supplementary Fig. 20. LC/MS analysis for the mono- and dihalogenation of compound **12**. The top row is for chlorination, and the bottom row is for iodination. The left column shows XIC spectra for **12** (orange), mono-halogenated **12** (blue), and dihalogenated **12** (pink). The middle and right columns show the MS spectra for mono-halogenated **12** and dihalogenated **12**, respectively. Inset shows zoom-in of the peak 37.796 min for dichlorinated **12**.

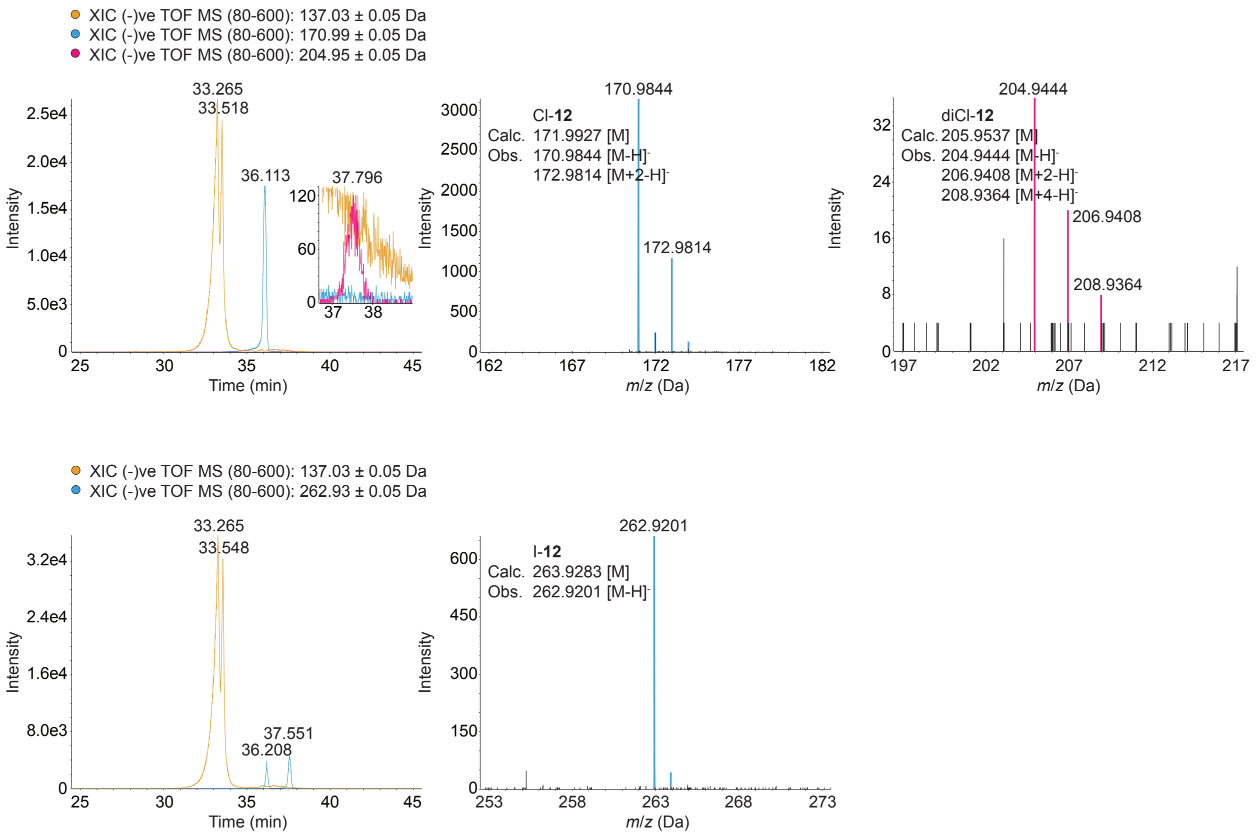

Supplementary Fig. 21. LC/MS analysis for the mono-halogenation of compound **13**. The top row is for chlorination, and the bottom row is for iodination. The left column shows XIC spectra for **13** (orange) and mono-halogenated **13** (blue). The right column shows the MS spectra for mono-halogenated **13**. Inset shows zoom-in of the peak at about 36.198, 36.740, and 36.980 min for mono-iodinated **13**.

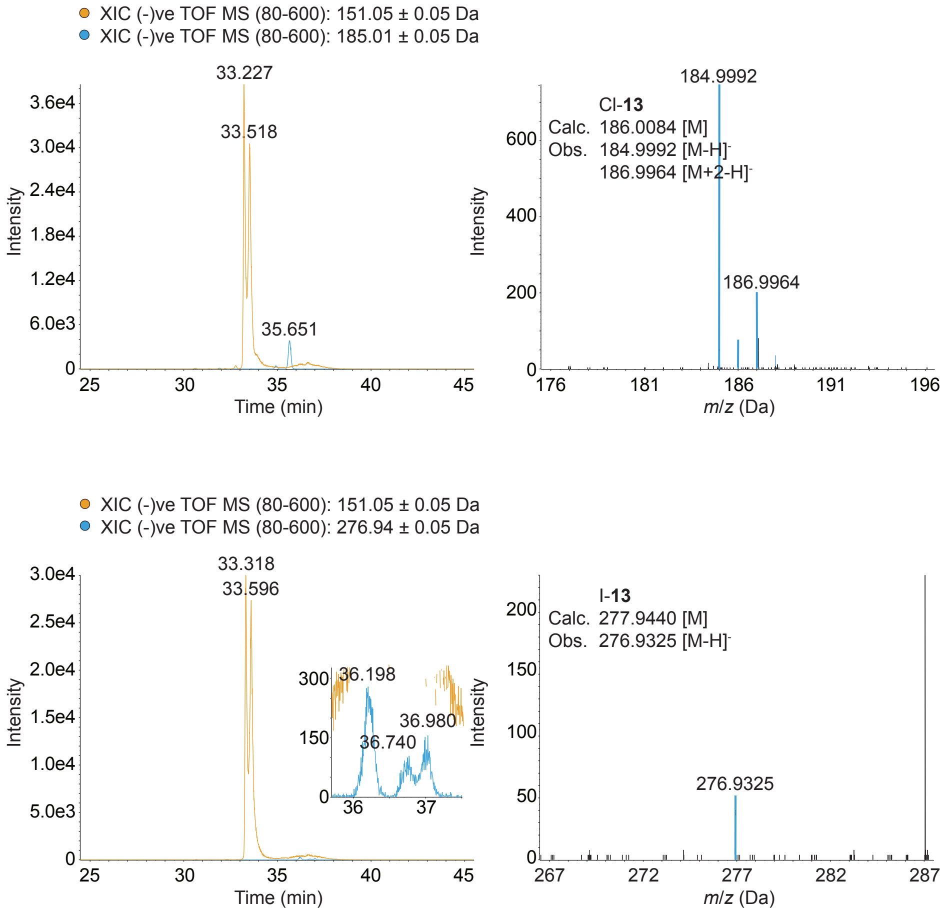

Supplementary Fig. 22. LC/MS analysis for the mono-iodination of compound **14**. The left column shows XIC spectrum for **14** (orange) and mono-iodinated **14** (blue). The right column shows the MS spectrum for mono-halogenated **14**. Inset shows zoom-in of the peak 37.380 min for mono-iodinated **14**.

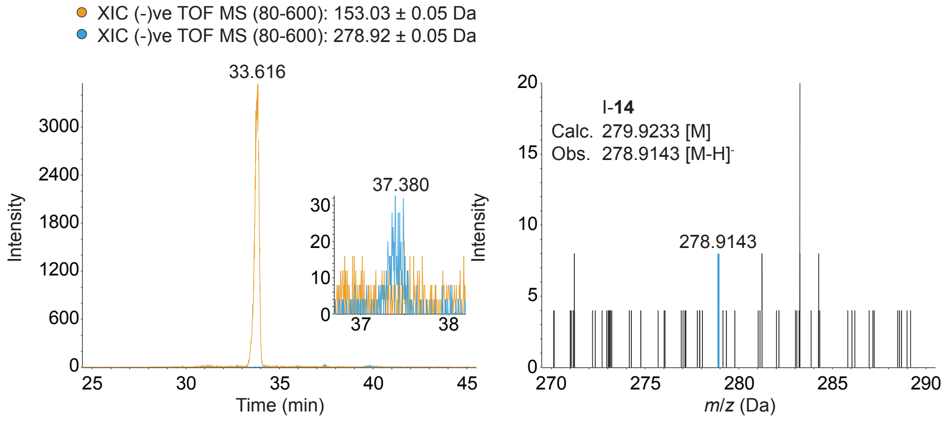

Supplementary Fig. 23. LC/MS analysis for the mono-halogenation of compound **15**. The top row is for chlorination, and the bottom row is for iodination. The left column shows XIC spectra for **15** (orange) and mono-halogenated **15** (blue). The right column shows the MS spectra for mono-halogenated **15**. Insets show zoom-in to show peak intensities for chlorinated and iodinated **15** on top and bottom panel, respectively

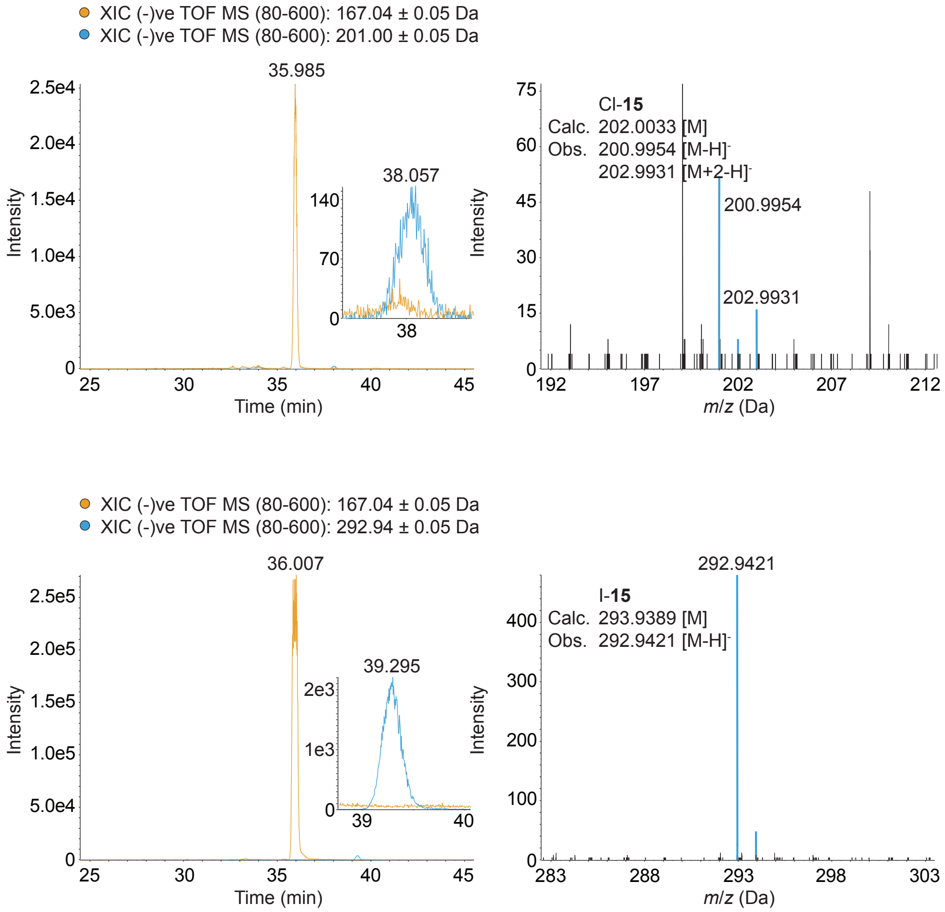

Supplementary Fig. 24. LC/MS analysis for the mono- and dihalogenation of compound **16**. The top row is for chlorination, and the bottom row is for iodination. The left column shows XIC spectra for **16** (orange), mono-halogenated **16** (blue), and dihalogenated **16** (pink). The middle and right columns show the MS spectra for mono-halogenated **16** and dihalogenated **16**, respectively.

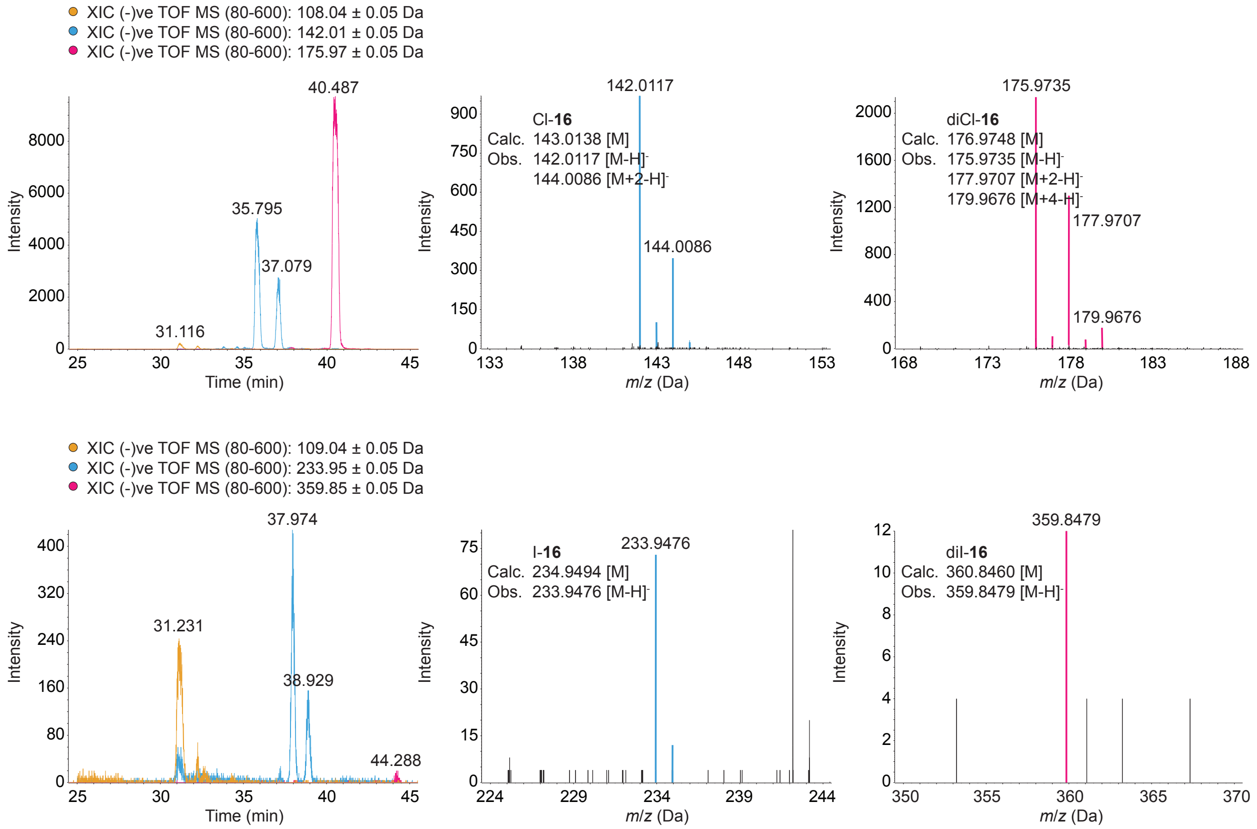

Supplementary Fig. 25. LC/MS analysis for the mono-halogenation of compound **17**. The top row is for chlorination, and the bottom row is for iodination. The left column shows XIC spectra for **17** (orange) and mono-halogenated **17** (blue). The right column shows the MS spectra for mono-halogenated **17**.

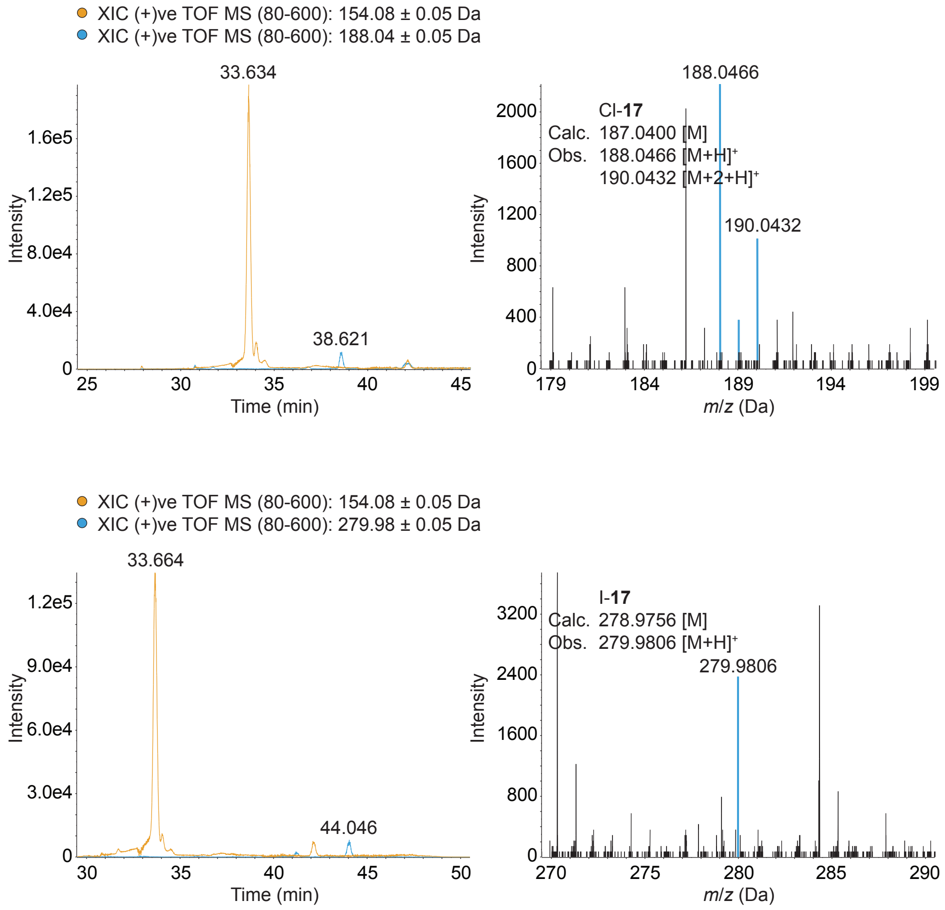

Supplementary Fig. 26. LC/MS analysis for the mono-chlorination of compound **18**. The left column shows XIC spectrum for **18** (orange) and mono-chlorinated **18** (blue). The right column shows the MS spectrum for mono-chlorinated **18**. Inset shows zoom-in of the peak at 30.101 min for unmodified **18**.

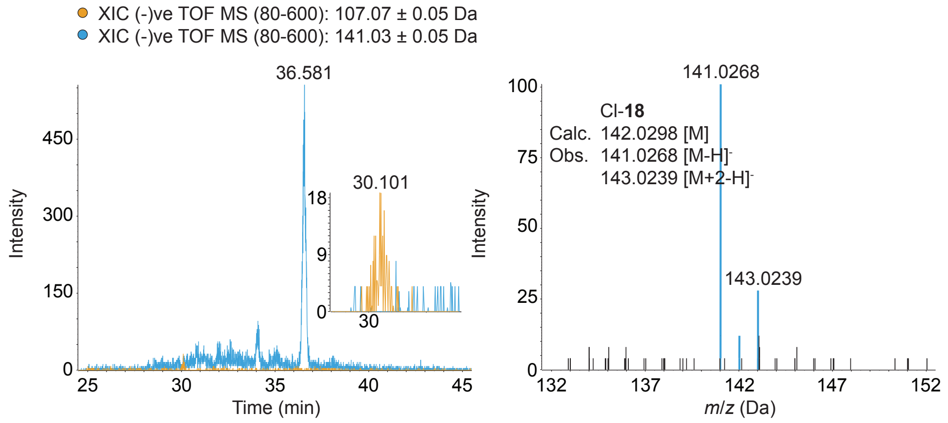

Supplementary Fig. 27. LC/MS analysis for the mono- and diiodination of compound **21**. The left column shows XIC spectrum for **21** (orange), mono-iodinated **21** (blue), and diiodinated **21** (pink). The middle and right columns show the MS spectra for mono-iodinated **21**. Inset shows zoom-in of the peak at 29.804 min for diiodinated **21**.

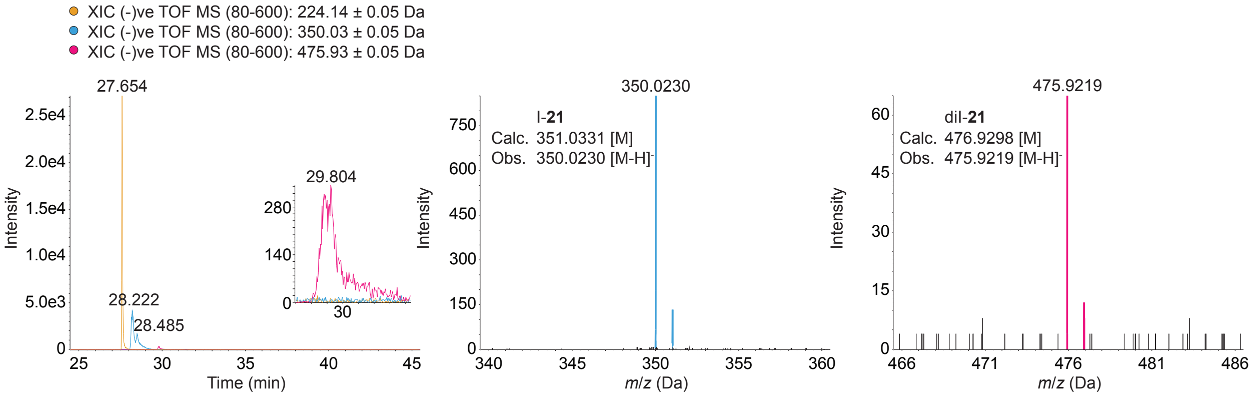

Supplementary Fig. 28. LC/MS analysis for the mono-iodination of compound **22**. The left column shows XIC spectrum for **22** (orange) and mono-iodinated **22** (blue). The right column shows the MS spectrum for mono-iodinated **22**. Inset shows zoom-in of the peak at 32.154 min for mono-iodinated **22**.

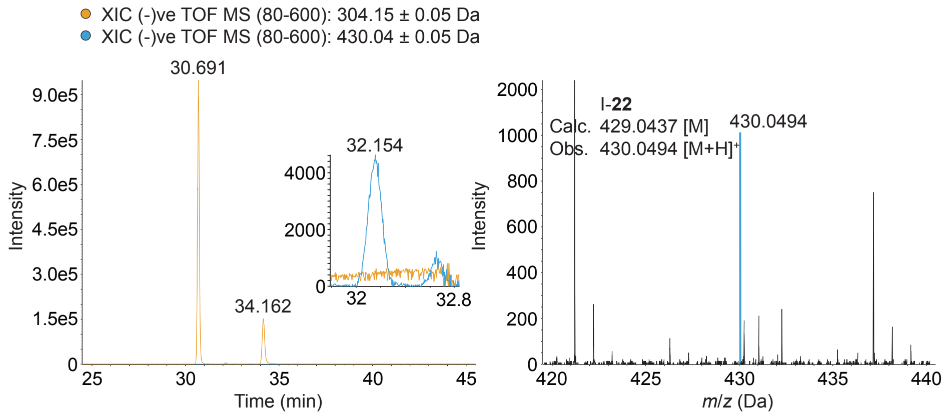

Supplementary Fig. 29. LC/MS analysis for the mono-halogenation of compound **23**. The top row is for chlorination, and the bottom row is for iodination. The left column shows XIC spectra for **23** (orange) and mono-halogenated **23** (blue). The right column shows the MS spectra for mono-halogenated **23**.

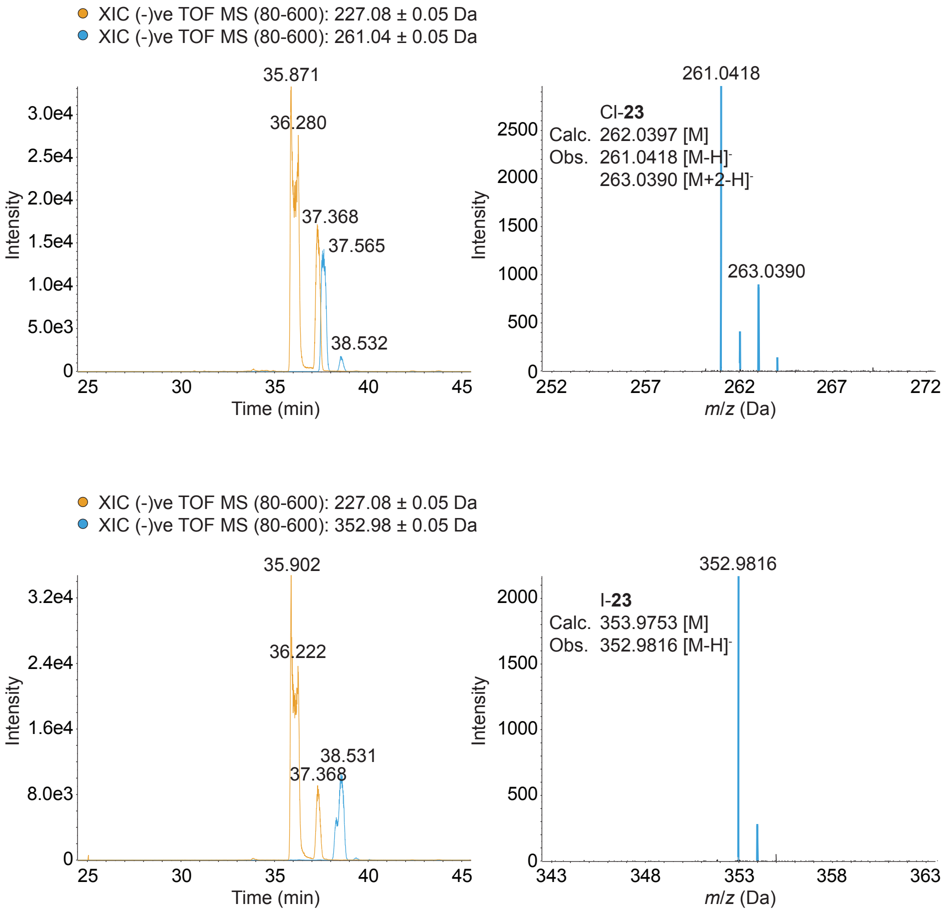

Supplementary Fig. 30. LC/MS analysis for the mono-halogenation of compound **24**. The top row is for chlorination, and the bottom row is for iodination. The left column shows XIC spectra for **24** (orange) and mono-halogenated **24** (blue). The right column shows the MS spectra for mono-halogenated **24**. Inset shows zoom-in of the peak at 33.137 min for mono-chlorinated **24**.

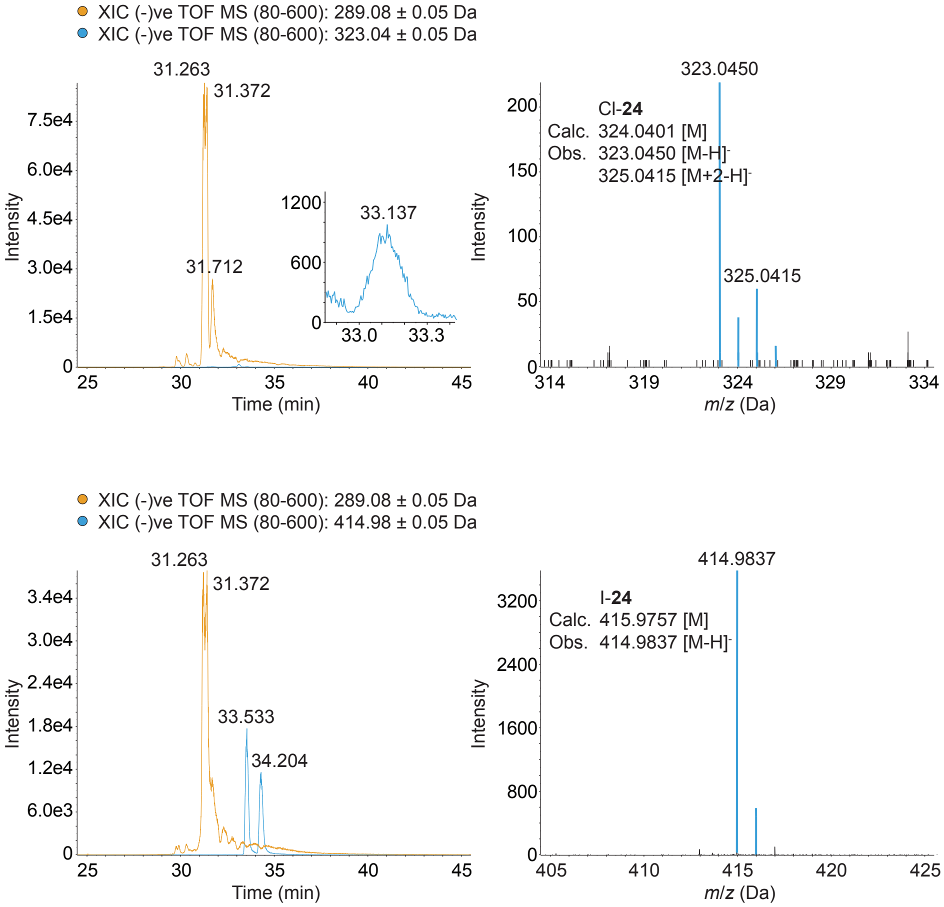

Supplementary Fig. 31. Structure-based sequence alignment of PltM, PltA and RebH. The alignment was obtained from the superimposition of crystal structures of PltM (PDB: 6BZN), PltA, PDB: 5DBJ[11] and RebH PDB: 2OA1.[7, 16-17] Conserved residues are shown by red boxes. The conserved FAD binding motifs are underlined and labeled in orange. Residues mutated in this study are indicated by navy rectangles. The catalytic lysine residue, K87 is indicated by a yellow oval. The flexible FAD interacting loop of PltM is highlighted and labeled in brown.

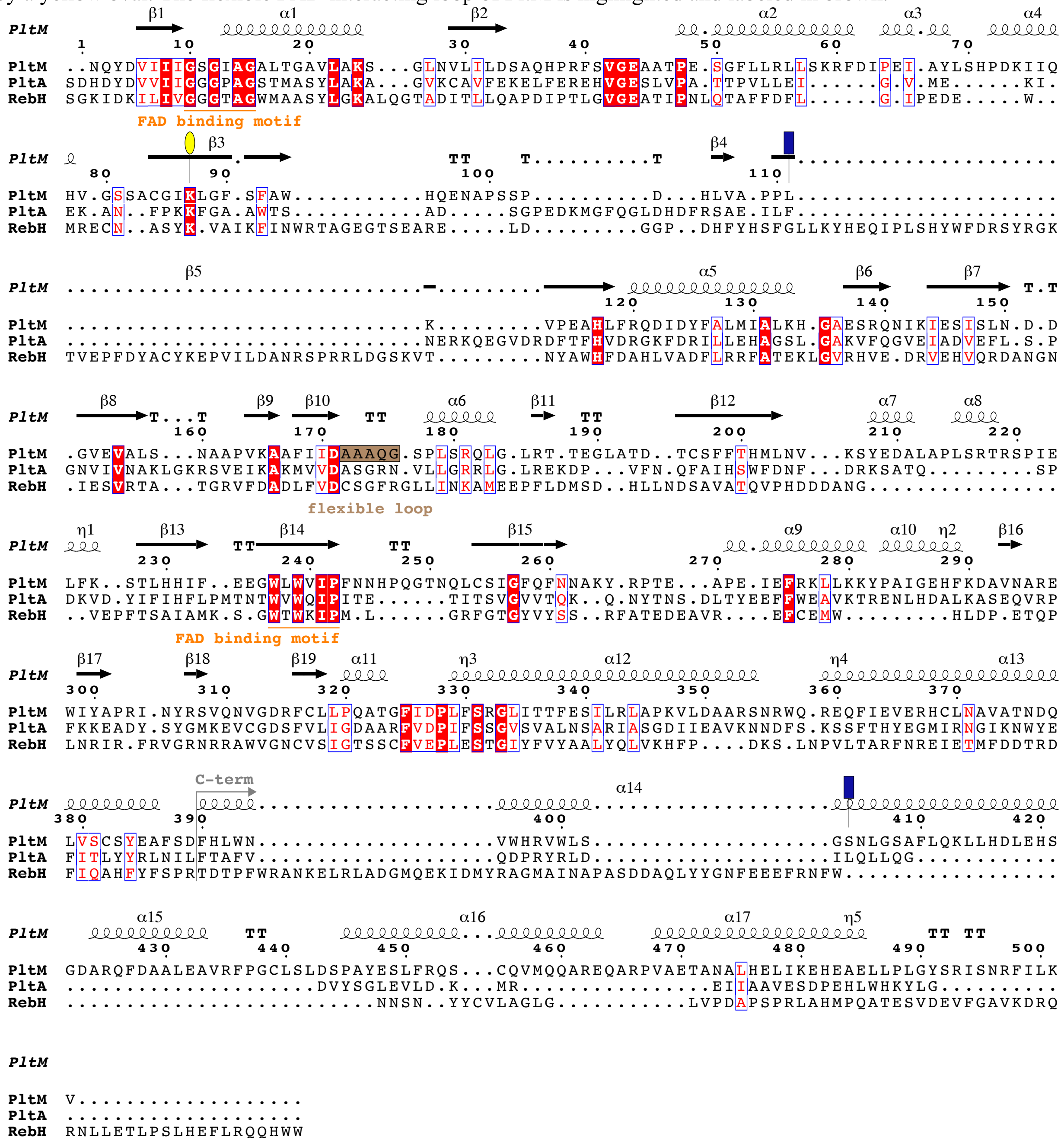

Supplementary Fig. 32. PltM preparation and crystals. **A.** The S-200 size-exclusion chromatograms of PltM and PltA (left panel). A picture of a 15% SDS-PAGE gel showing purified PltM (right panel). **B.** Concentrated PltM and crystals of PltM. Concentrated PltA and its crystals, obtained as described previously,[11] are shown for comparison.

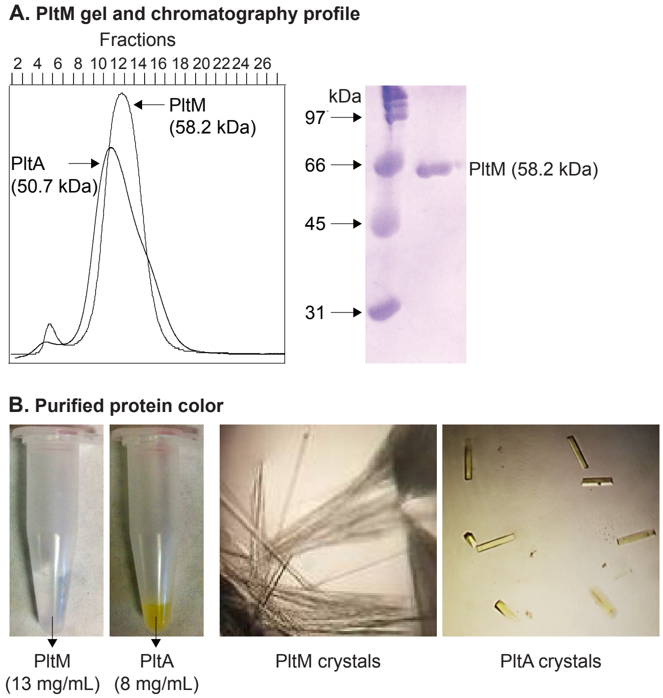

Supplementary Fig. 33. Structural comparison of the substrate binding site of various FAD-dependent halogenases. **A.** The structure of PltM. The conserved N-terminal region is colored pale yellow and the C-terminal region is colored orange. **B.** A zoomed in view of the substrate binding site of PltM in complex with compound **1** (yellow sticks). **C.** The structure of RebH in complex with bound L-Trp (grey sticks; PDB: 2OA1[7]). The N-terminal and C-terminal regions are in purple and yellow, respectively. **D.** A zoomed in view of the substrate binding site of RebH. **E.** The structure of PltA (PDB ID: 5DBJ[11]). The N-terminal region is shown in teal, and the C-terminal region occluding the substrate binding site is shown in blue. **F.** A zoomed in view of the substrate binding site of PltA.

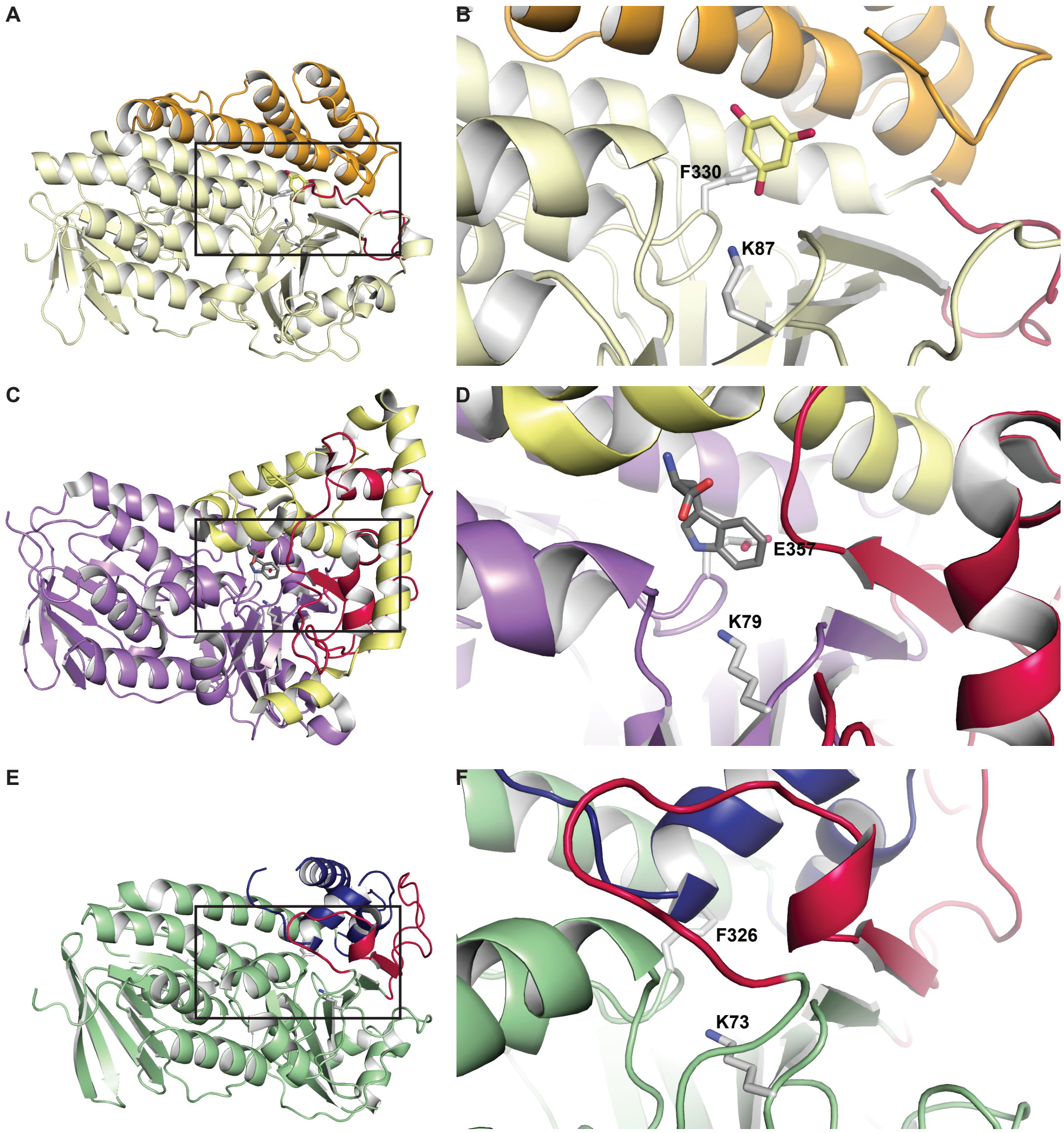

Supplementary Fig. 34. The *in vitro* analysis of PltM K87A by using **11** as the substrate. Wild-type PltM yields diCl-**11** at these conditions.

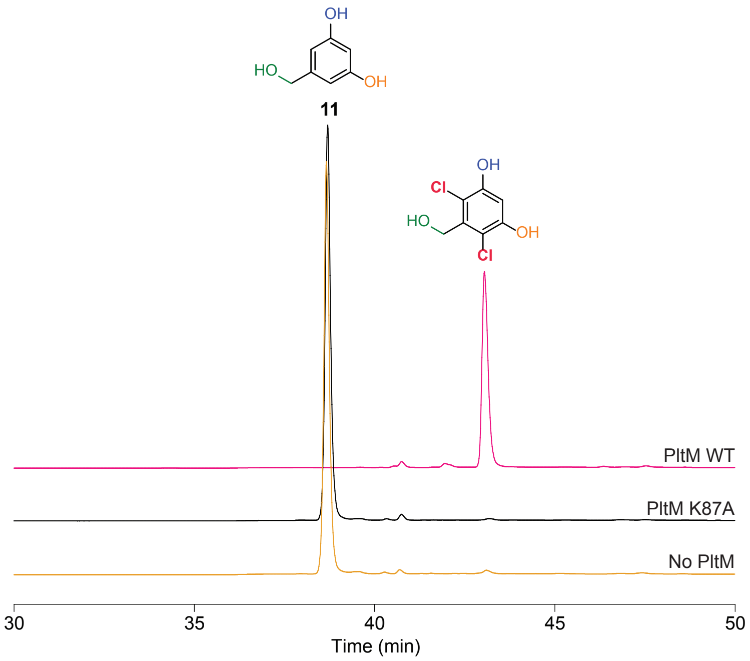

Supplementary Fig. 35. A. The PltM substrate binding site with a modeled diiodinated compound **1**. **B.** An alternative view of the substrate binding site with the model of diiodinated compound **1**.

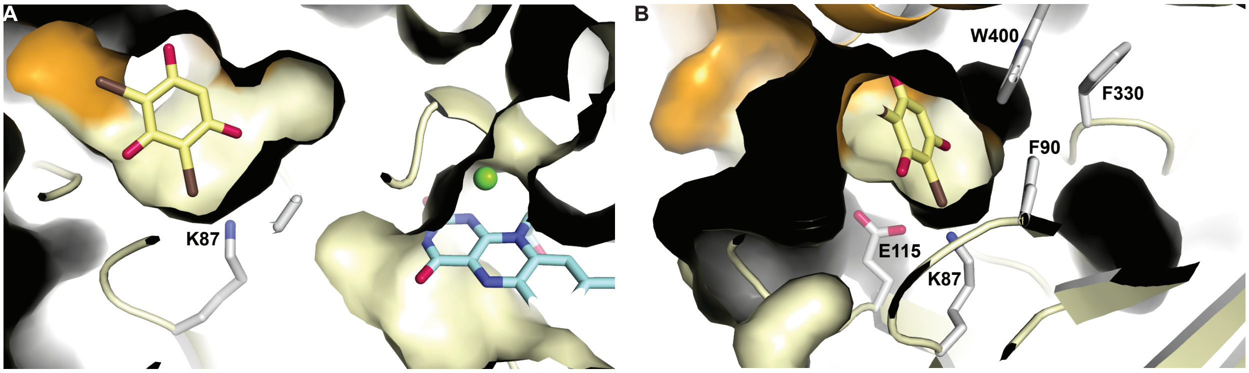

Supplementary Fig. 36. Structural comparison of the FAD binding site of various FAD-dependent halogenases. The FAD binding site is represented as surface and cartoon for **A.** and **B.** PltM (PDB ID: 6BZQ and 6BZT), **C.** and **D.** RebH (PDB: 2OA1[7]), **E.** and **F.** PltA (PDB: 5DBJ[11]), respectively. The FAD (light blue sticks) is encased by the residues (shown as grey sticks) for PltA. Corresponding residues for PltM and RebH are indicated by grey sticks. The chloride ion is shown as a green sphere.

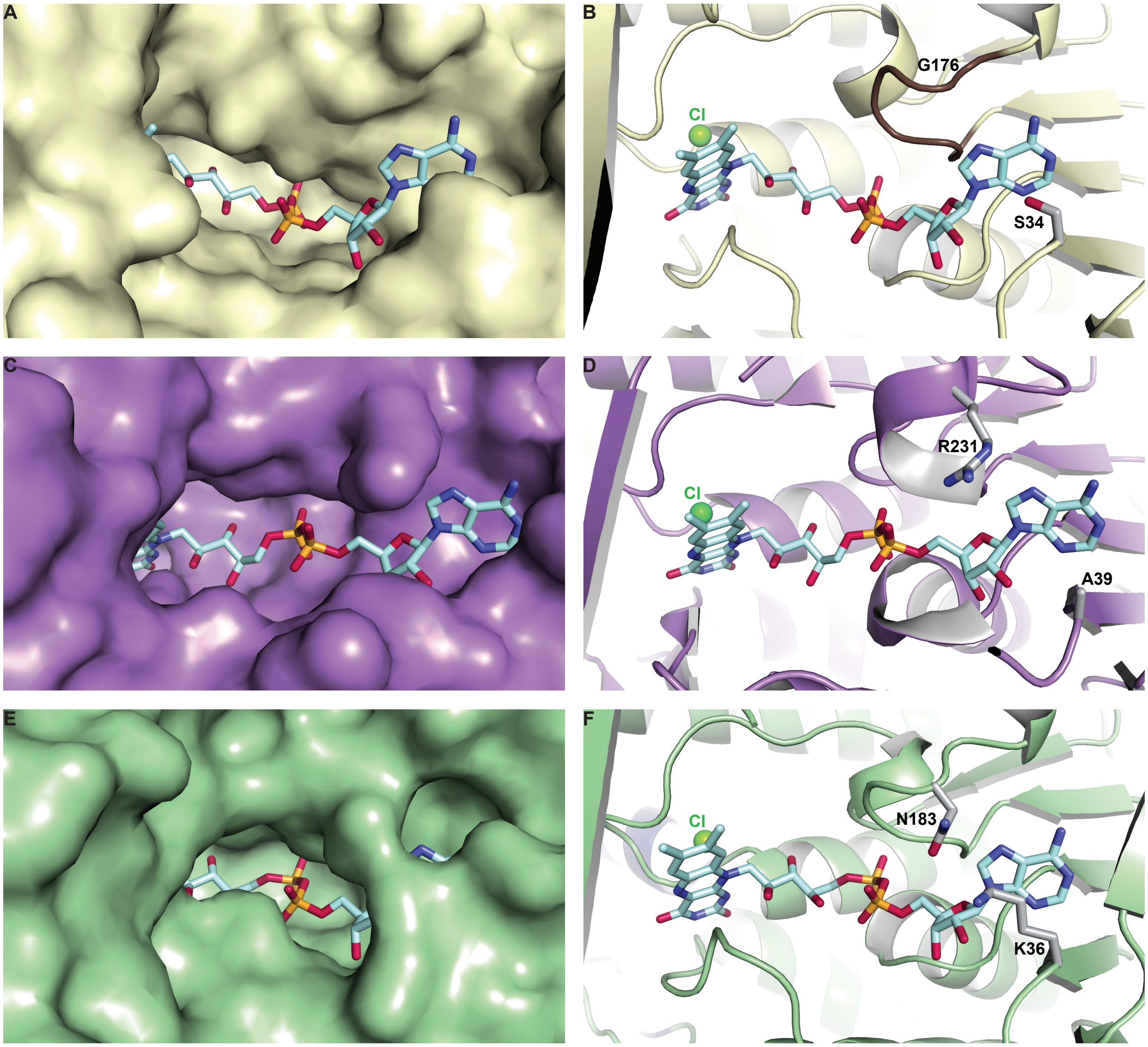

Supplementary Fig. 37. The omit electron density maps for FAD and chloride. **A.** The FAD in the structure of PltM and chloride are well defined by the F_o-F_c omit map contoured at 3σ (maroon mesh) for **A.** PltM L111Y and 2.5σ for **B.** PltM-WT in complex with fully bound FAD. **C.** The isoalloxazine ring is well defined by the F_o-F_c omit map contoured at 3σ in the complex of PltM with partially bound FAD. **D.** A view similar to **C**, but tilted to show the isoalloxazine ring. This view shows that the right-hand side ring, including the asymmetrically positioned oxygen atoms, is well resolved by the F_o-F_c omit map, unambiguously defining the orientation of the isoalloxazine ring. This position of the isoalloxazine ring is consistent with the interactions of the nonpolar left-hand side of the ring with surrounding nonpolar residues (Val42, Phe199, Trp239, and Pro328) and the polar right-hand side of the ring with the nearby hydroxyl of Tyr306 and surrounding solvent, as illustrated in panel **D**.

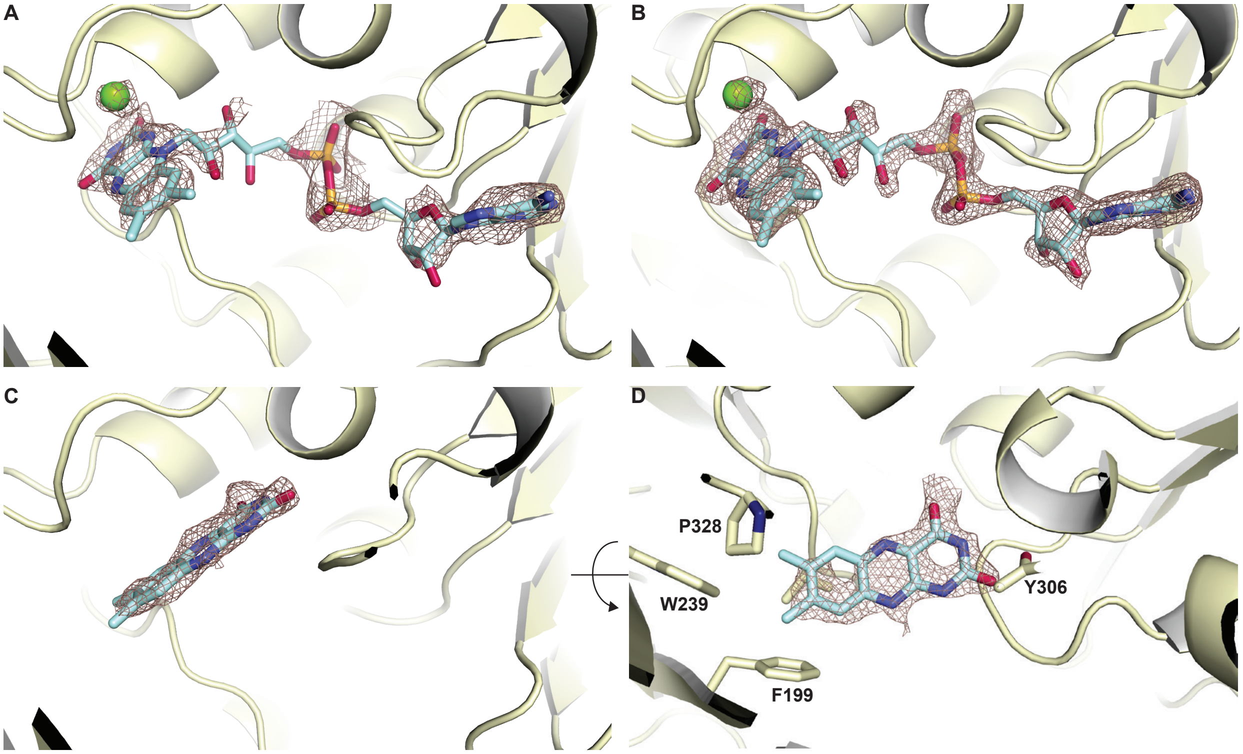

Supplementary Fig. 38. Steric overlap of Tyr at positions 111 and 404 with the substrate binding site. The substrate binding site of PltM L111Y, as observed in the crystal structure of PltM L111Y with the model of bound substrate **1** (as observed in the srtucture of PltM-substrate **1** complex) and a modeled S404Y mutation. Either tyrosine residue at positions 111 and 404 clashes sterically with substrate **1**.

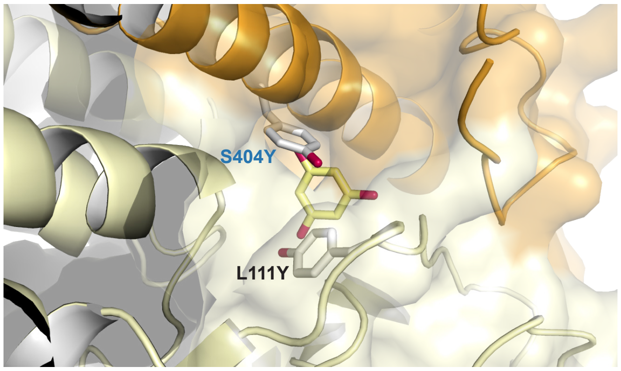

Supplementary Fig. 39. LC/MS analysis of halogenation reaction of **1** in the cell-based assays. The top row shows halogenation with PltM WT, the second row is with PltM L111Y mutant, the third row is with PltM S404Y mutant, and the bottom row is PltM K87A (left) and using PltA (right), which served as the negative control for the experiment. For the top three rows, the left column shows the XIC spectra for **1** (orange), mono-chlorinated **1** (blue), and dichlorinated **1** (pink). The middle and right columns show the MS spectra for mono-chlorinated **1** and dichlorinated **1**, respectively.

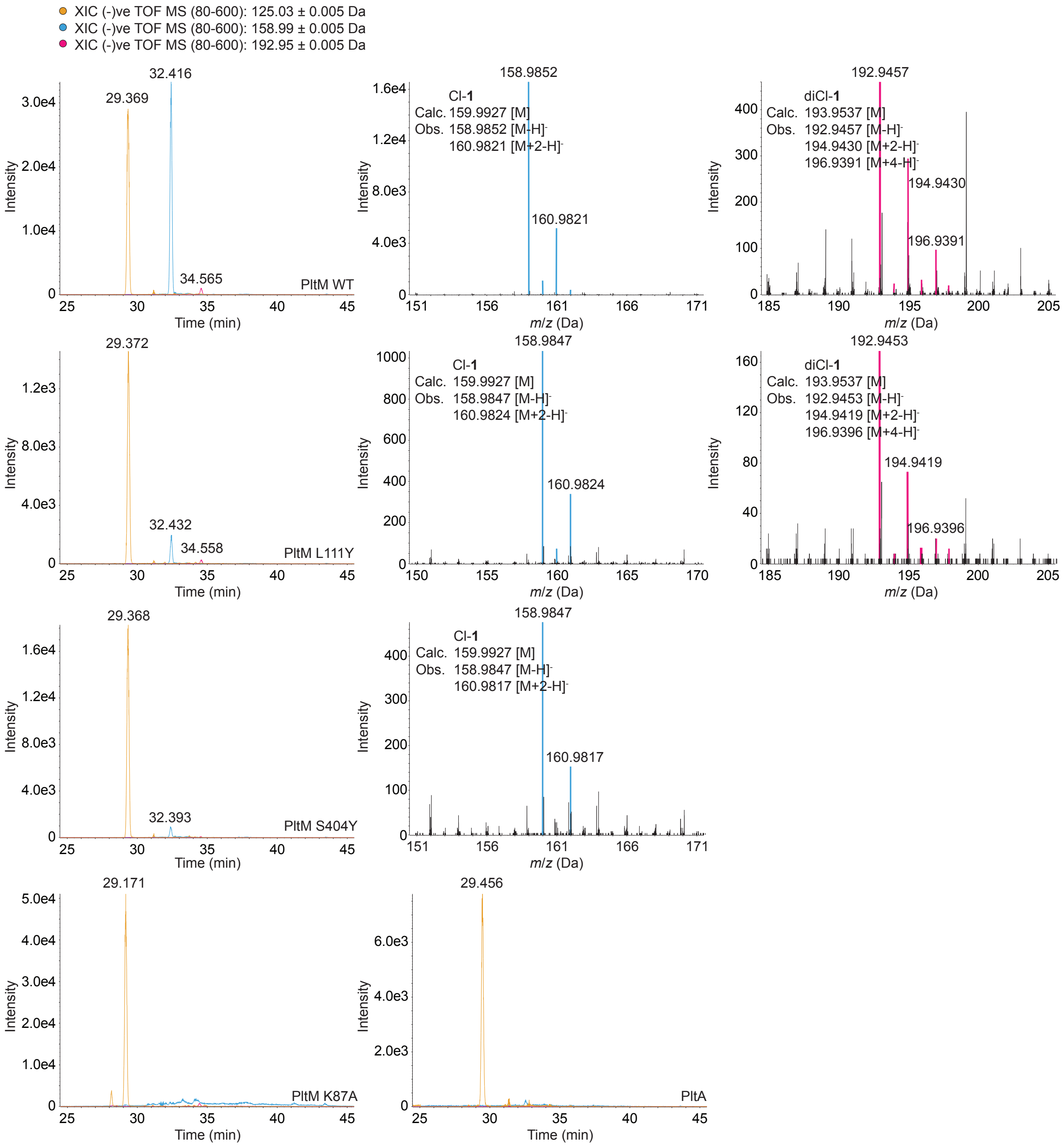

Supplementary Fig. 40. Time course experiments by using the new optimized *in vitro* reaction conditions. Substrates **3** (**A**, **B**, and **C**), **11** (**D**, **E**, and **F**), and **16** (**G**, **H**, and **I**) were used to measure chlorination (**A**, **D**, and **G**), bromination (**B**, **E**, and **H**), and iodination (**C**, **F**, and **I**). The orange circles, blue triangles, green squares, and pink diamonds indicate the % distribution of substrate, one mono-halogenated substrate, the second mono-halogenated substrate, and the dihalogenated substrate, respectively. The curves in panels **A**, **B**, **D**, **E**, **G**, and **H** represent the best fit of the dihalogenation mechanism parameters (see main text) to the data, while the curves in panels **C**, **F**, and **I** are best-fit single or double-exponential progress curves (here, enzyme was precipitating during reactions) by DynaFit.[18] The experiments were performed in duplicate.

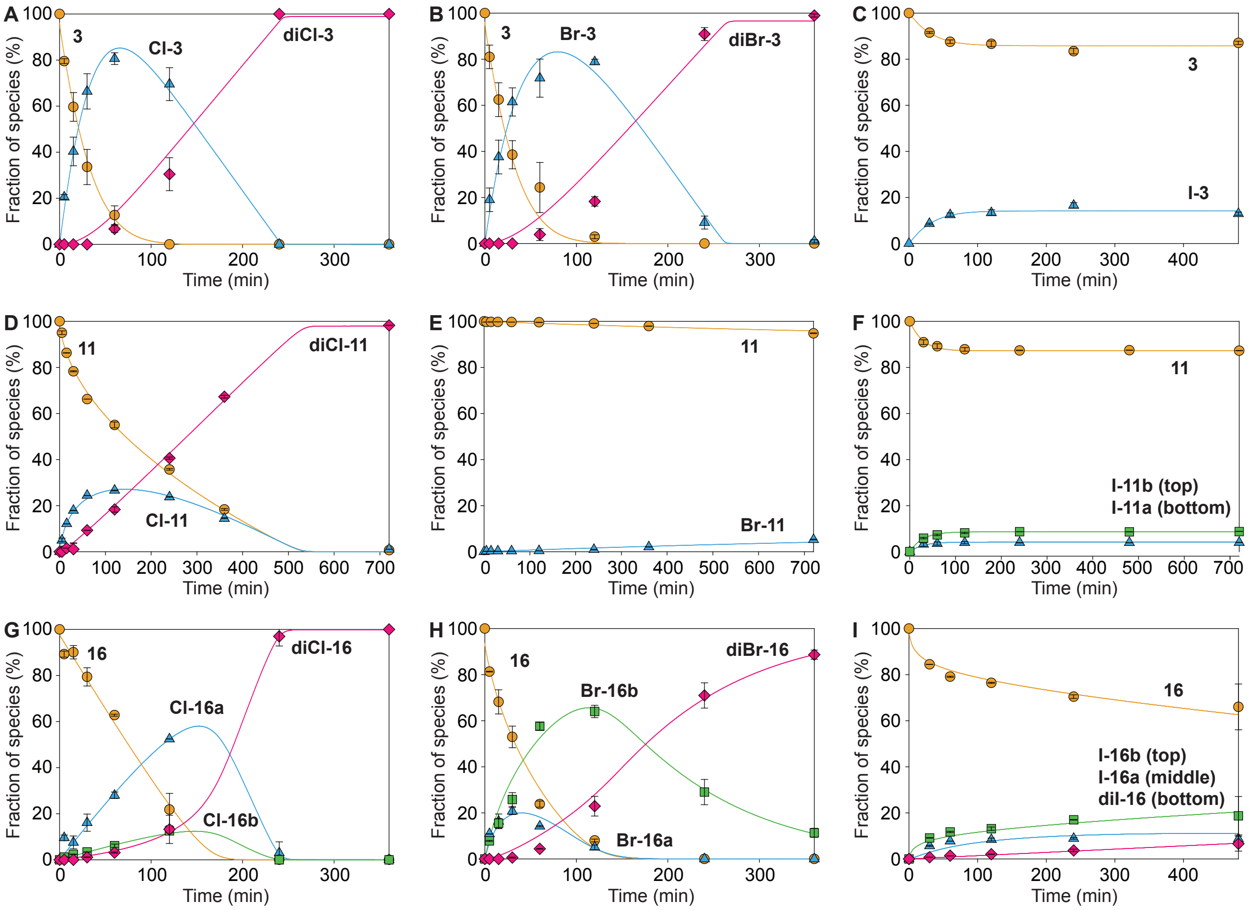

Supplementary Fig. 41. The reusability of Affi-Gel®-enzyme conjugate for the halogenation assay. Chlorination of **A.** compound **3**, and **B.** compound **11** was tested ten times (x-axis) by reusing the same Affi-Gel®-enzyme conjugate for each reaction. The reactions were repeated five times on the first day and five times again on the second day. The fractions of the substrate, mono-chlorinated product, and dichlorinated product are shown by orange, blue, and pink bars, respectively. The black dots show the overall halogenation % of each substrate. *Note*: The chlorination patterns shown for Cl-**3**, diCl-**3**, Cl-**11**, and diCl-**11** were established by NMR spectroscopy.

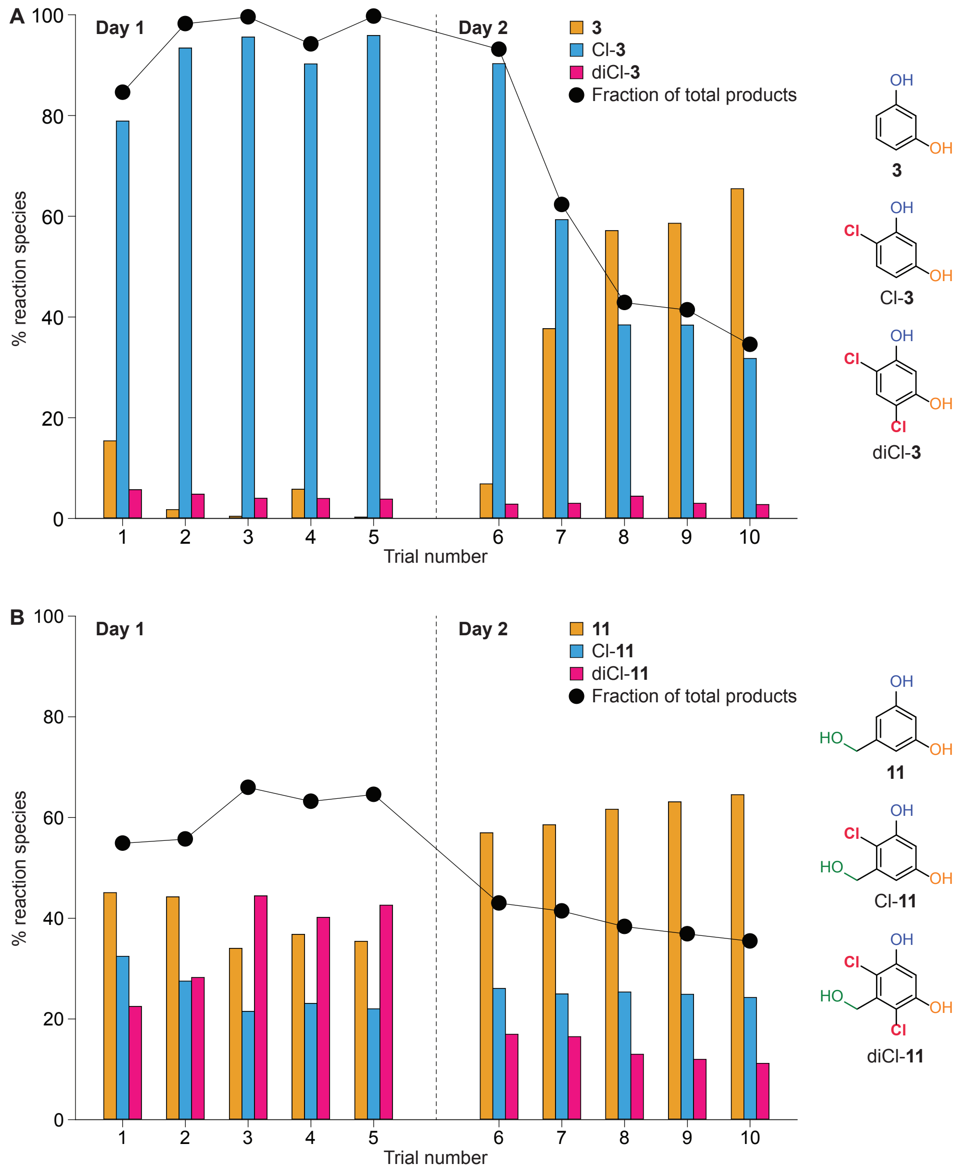

Supplementary Fig. 42. ^{1}H NMR spectrum for compound 4,6-dichlororesorcinol (4,6-diCl-**3**) in CD_3OD (500 MHz).

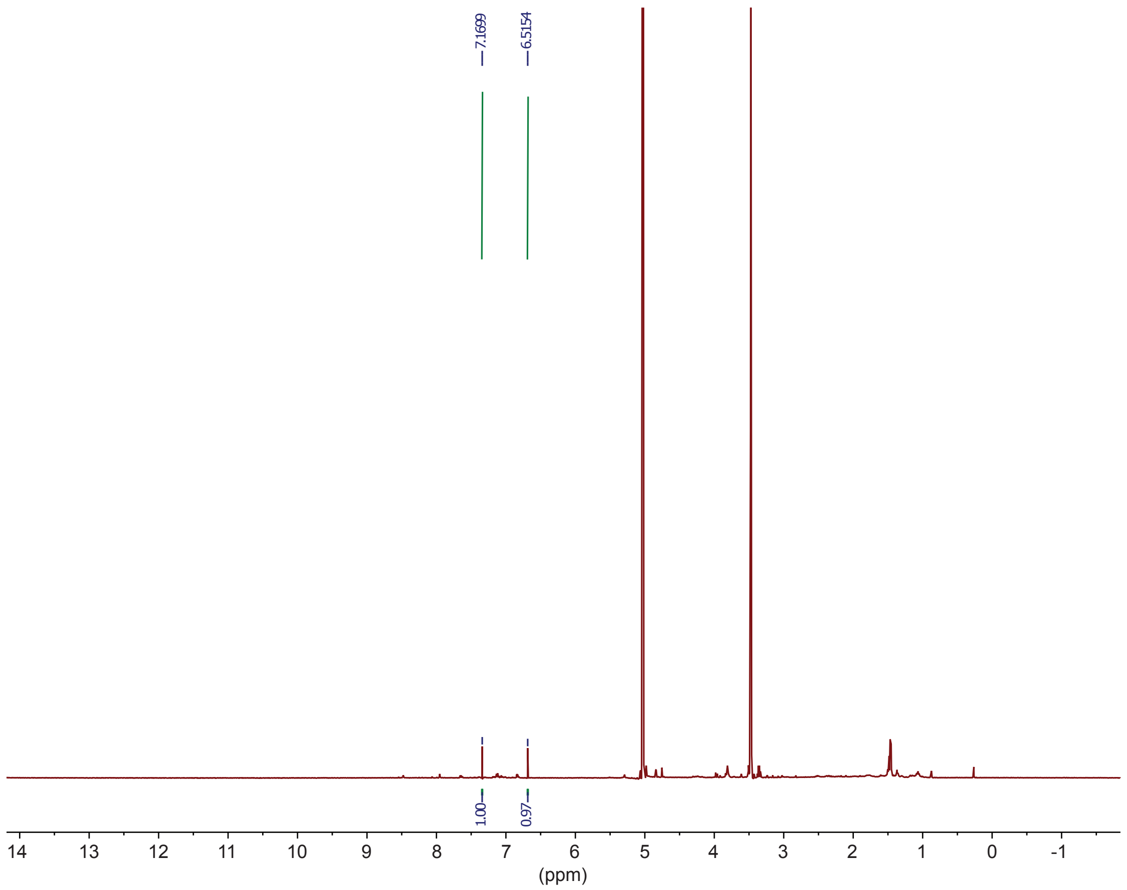

Supplementary Fig. 43. ^{1}H NMR spectrum for compound 4,6-dibromoresorcinol (4,6-diBr-**3**) in CD_3OD (500 MHz).

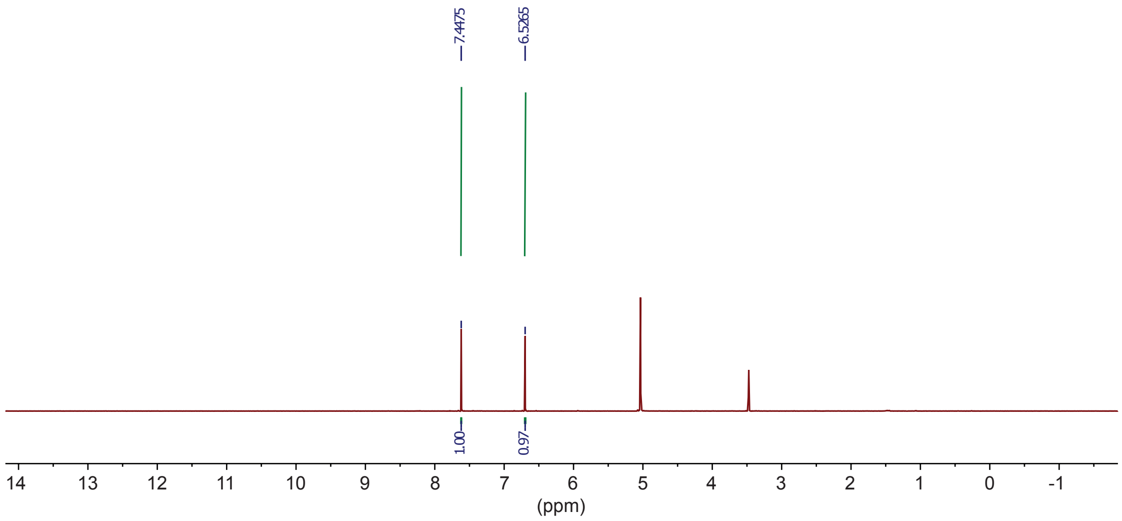

Supplementary Fig. 44. ^{13}C NMR spectrum for compound 4,6-dibromoresorcinol (4,6-diBr-**3**) in CD_3OD (100 MHz).

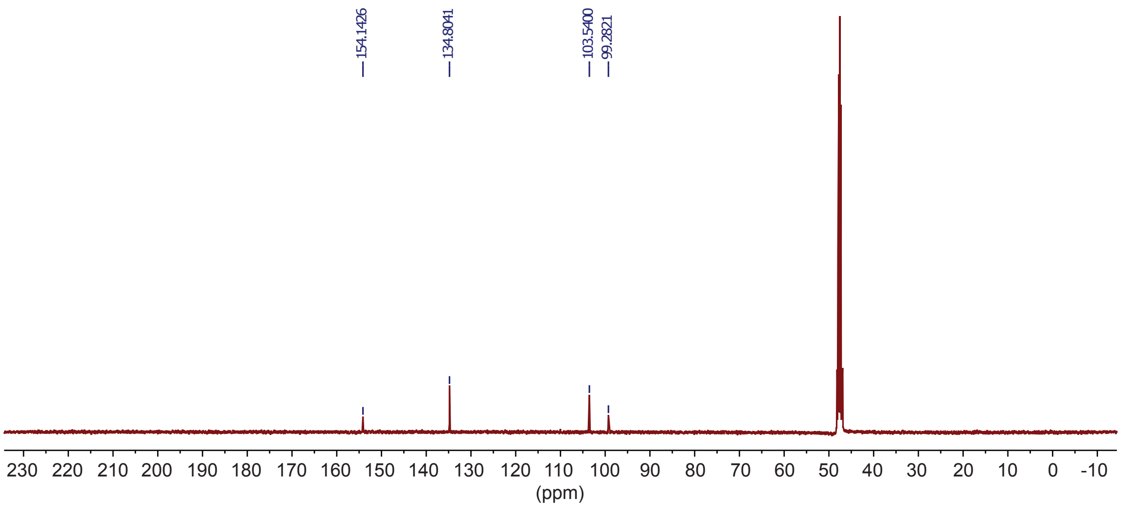

Supplementary Fig. 45. ^{1}H NMR spectrum for compound 2,4,6-trichlororesorcinol (4,6-diCl-**8**) in CD_3OD (500 MHz).

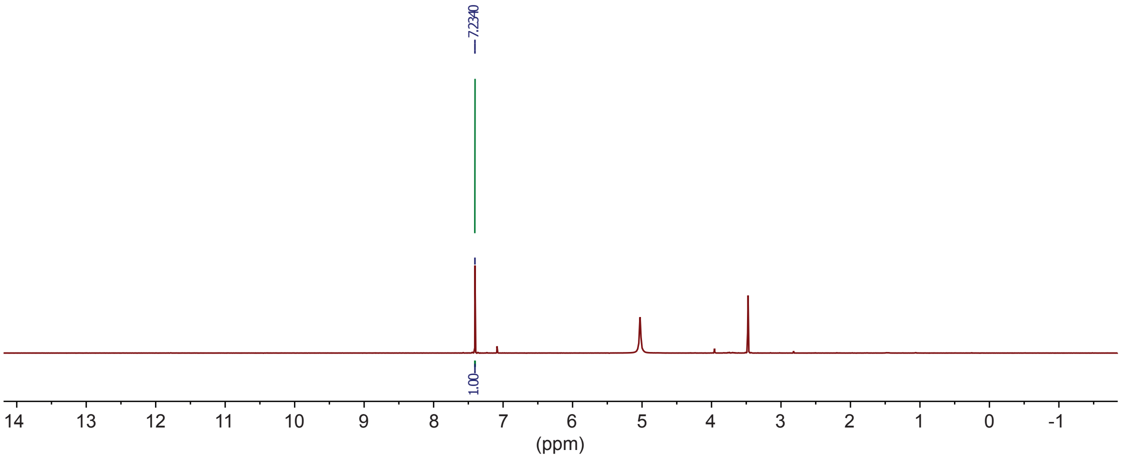

Supplementary Fig. 46. ^{1}H NMR spectrum for compound 2,4-dichloro-5-methylresorcinol (2,4-diCl-**9**) in CD_3OD (500 MHz).

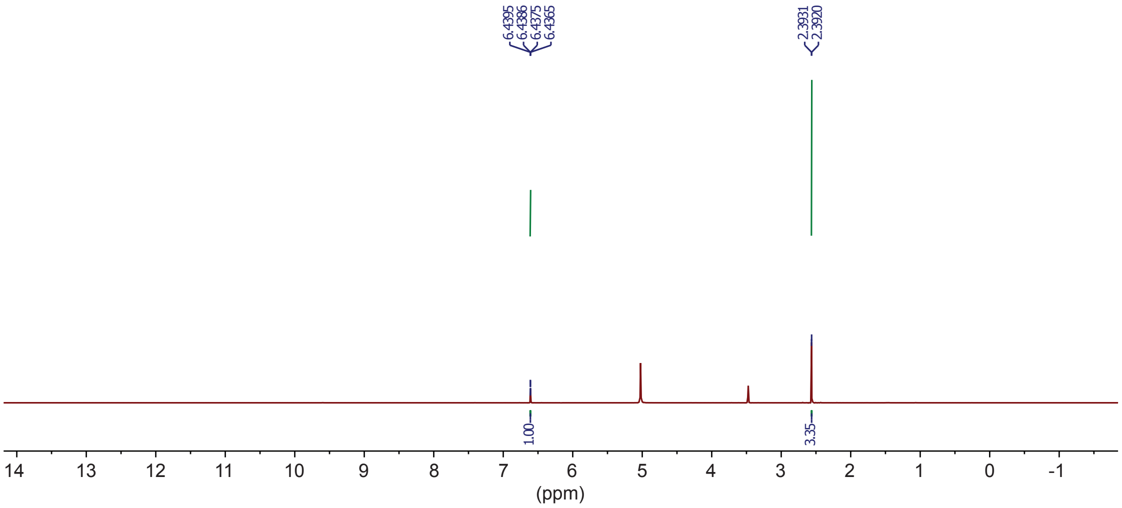

Supplementary Fig. 47. ^{13}C NMR spectrum for compound 2,4-dichloro-5-methylresorcinol (2,4-diCl-**9**) in CD_3OD (100 MHz).

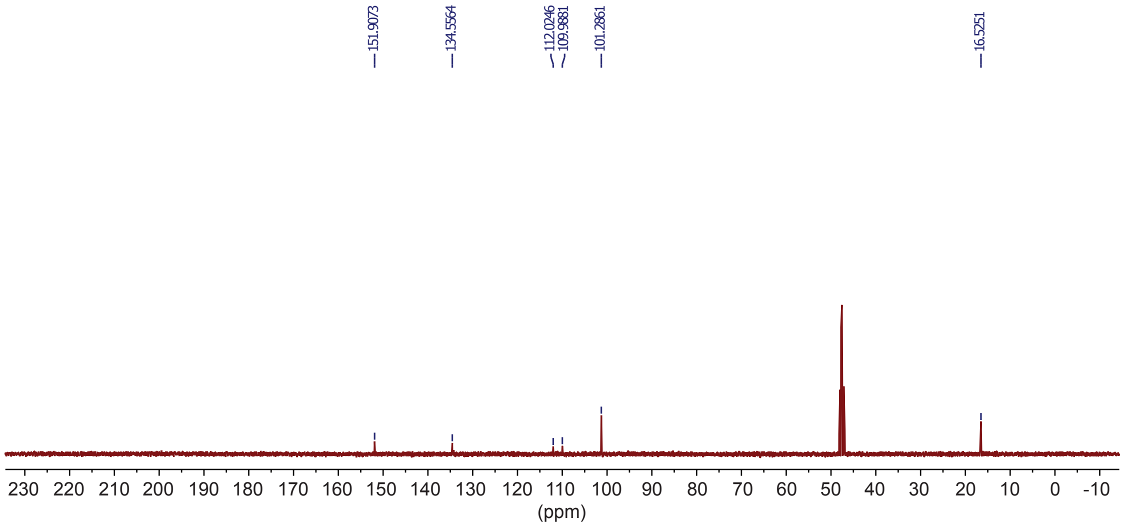

Supplementary Fig. 48. ^{1}H NMR spectrum for compound 2,6-dichloro-3,5-dihydroxybenzyl alcohol (2,6-diCl-**11**) in CD_3OD (500 MHz).

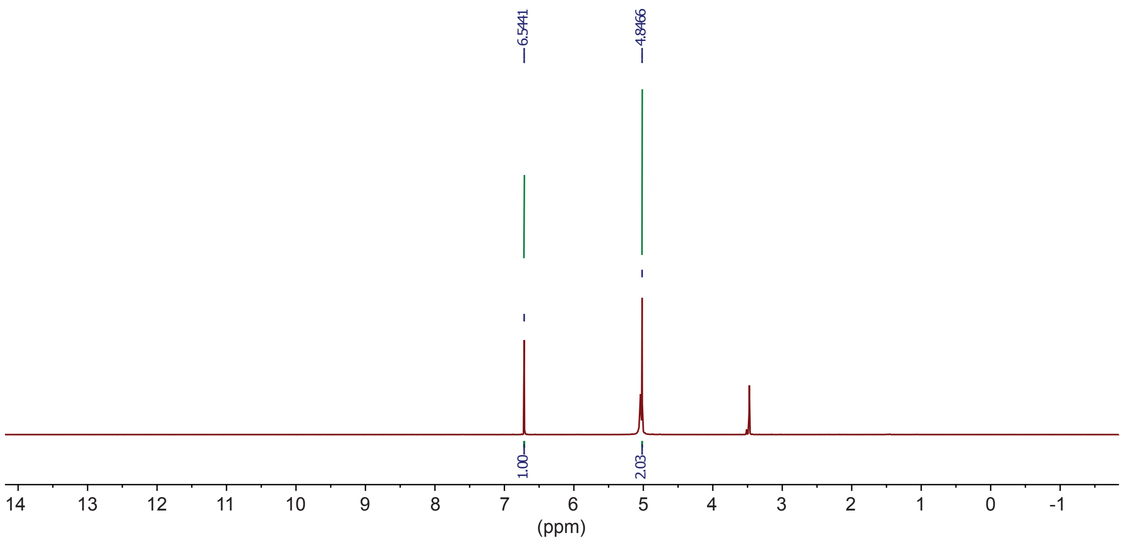

Supplementary Fig. 49. ^{13}C NMR spectrum for compound 2,6-dichloro-3,5-dihydroxybenzyl alcohol (2,6-diCl-**11**) in CD_3OD (100 MHz).

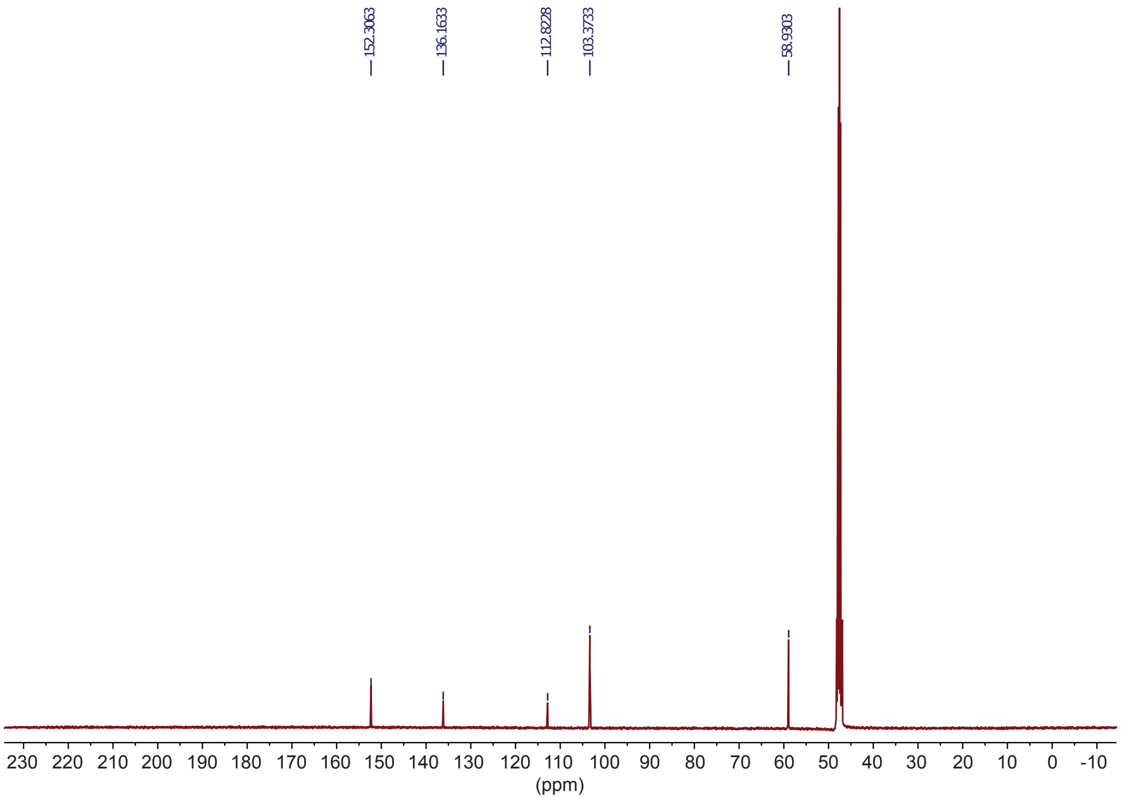

Supplementary Fig. 50. HMBC spectrum for compound 2,6-dichloro-3,5-dihydroxybenzyl alcohol (2,6-diCl-**11**) in CD_3OD (100 MHz).

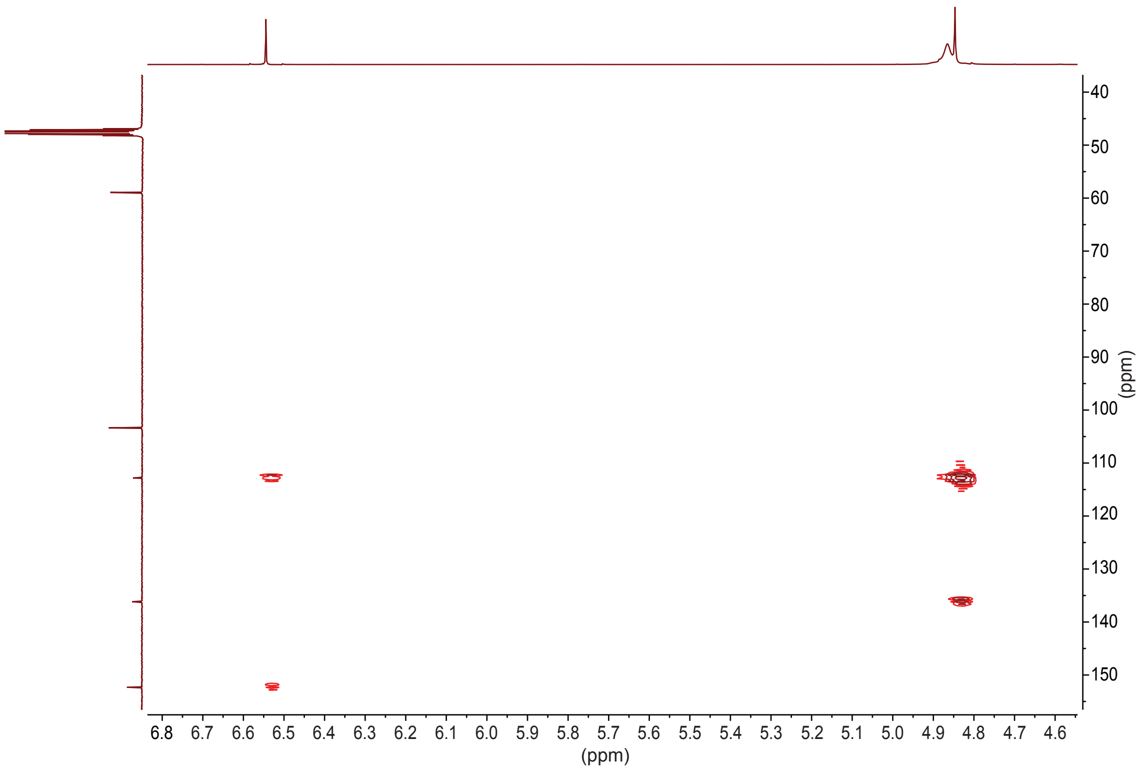

Supplementary Fig. 51. ^{1}H NMR spectrum for compound 3,5-dichloro-2,4,6-trihydroxyacetophenone (3,5-diCl-**15**) in CD_3OD (500 MHz).

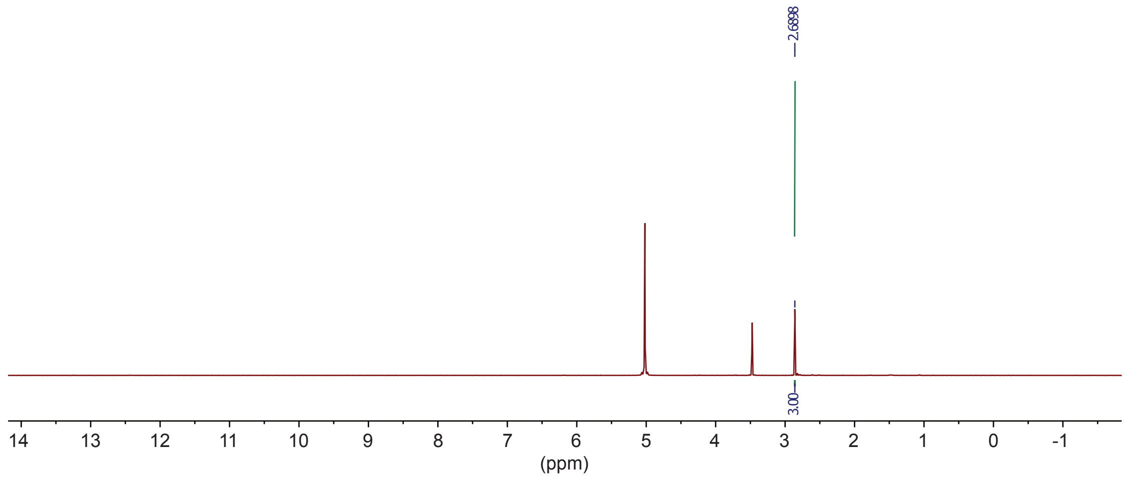

Supplementary Fig. 52. ^{1}H NMR spectrum for compound 5-amino-2,4-dichlorophenol (2,4-diCl-**16**) in CD_3OD (400 MHz).

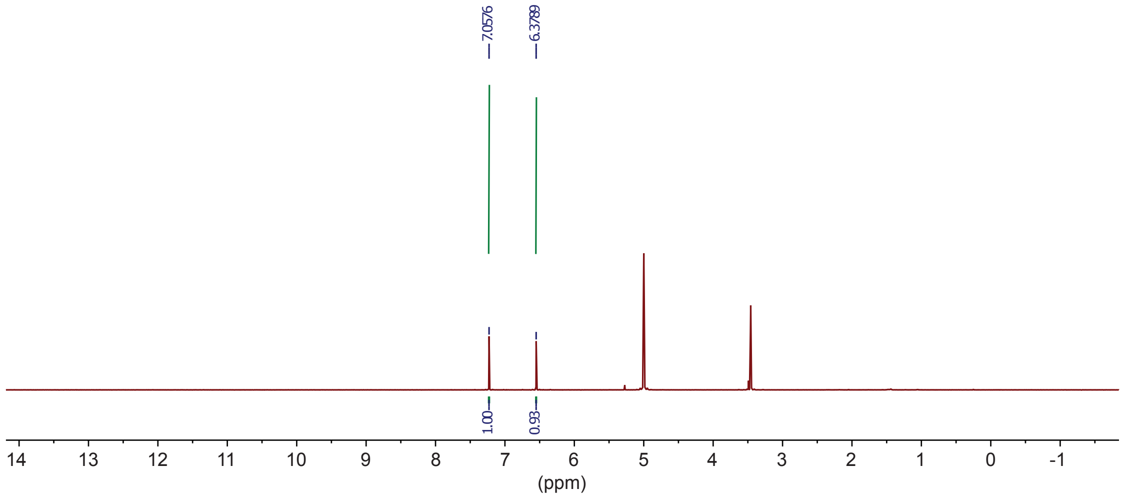

Supplementary Fig. 53. ^{13}C NMR spectrum for compound 5-amino-2,4-dichlorophenol (2,4-diCl-**16**) in CD_3OD (100 MHz).

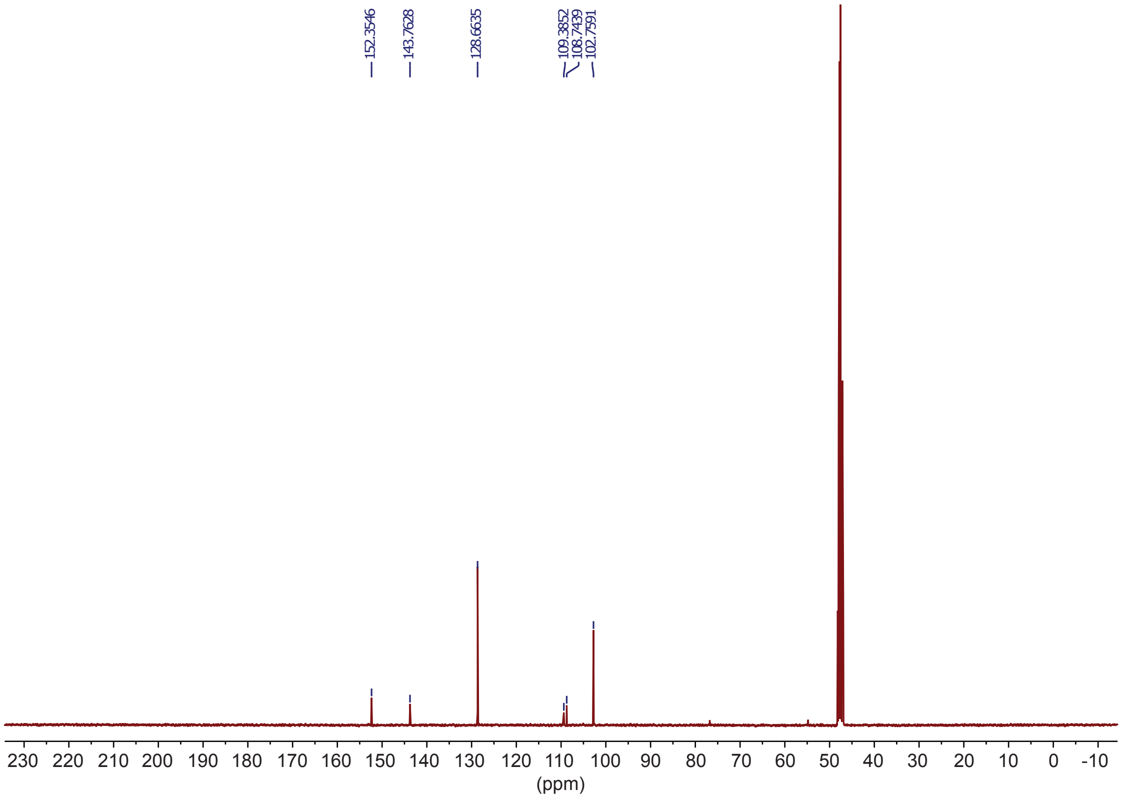

Supplementary Fig. 54. HSQC spectrum for compound 5-amino-2,4-dichlorophenol in CD_3OD (2,4-diCl-**16**) (100 MHz).

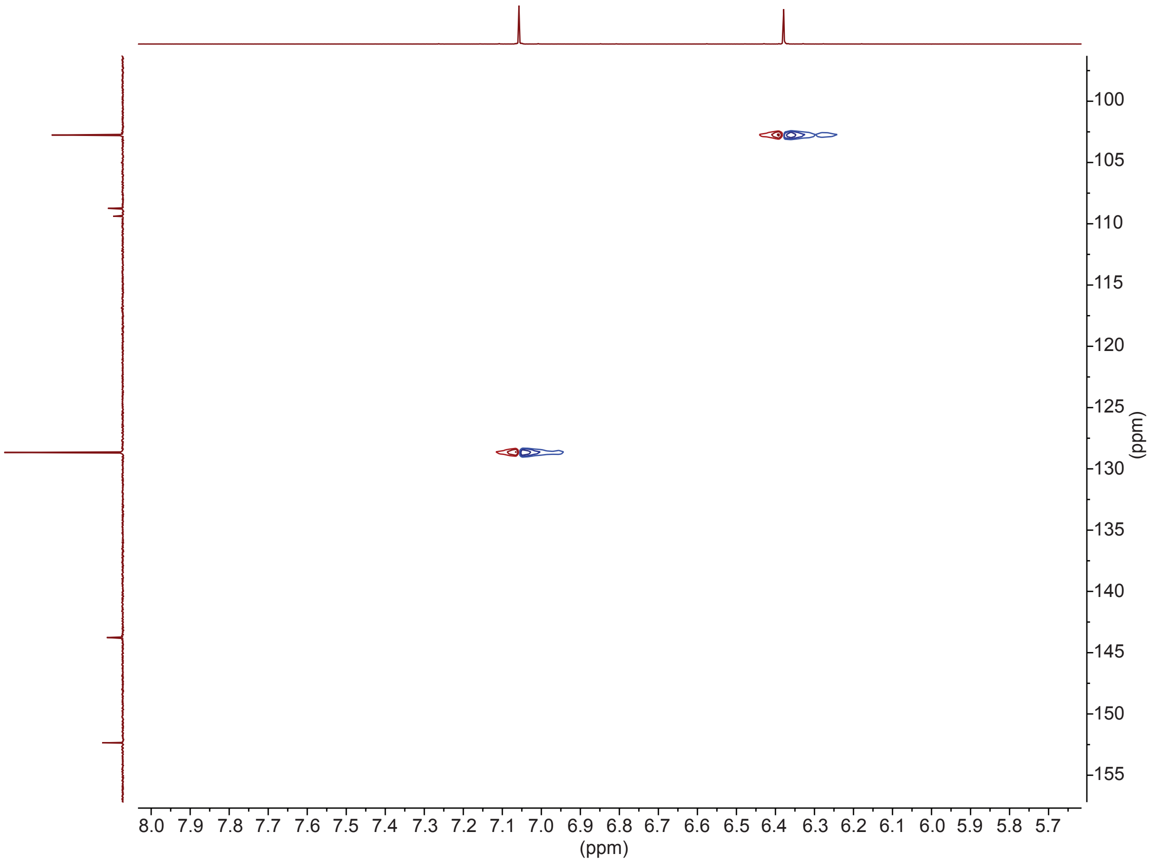

Supplementary Fig. 55. HMBC spectrum for compound 5-amino-2,4-dichlorophenol (2,4-diCl-**16**) in CD_3OD (100 MHz).

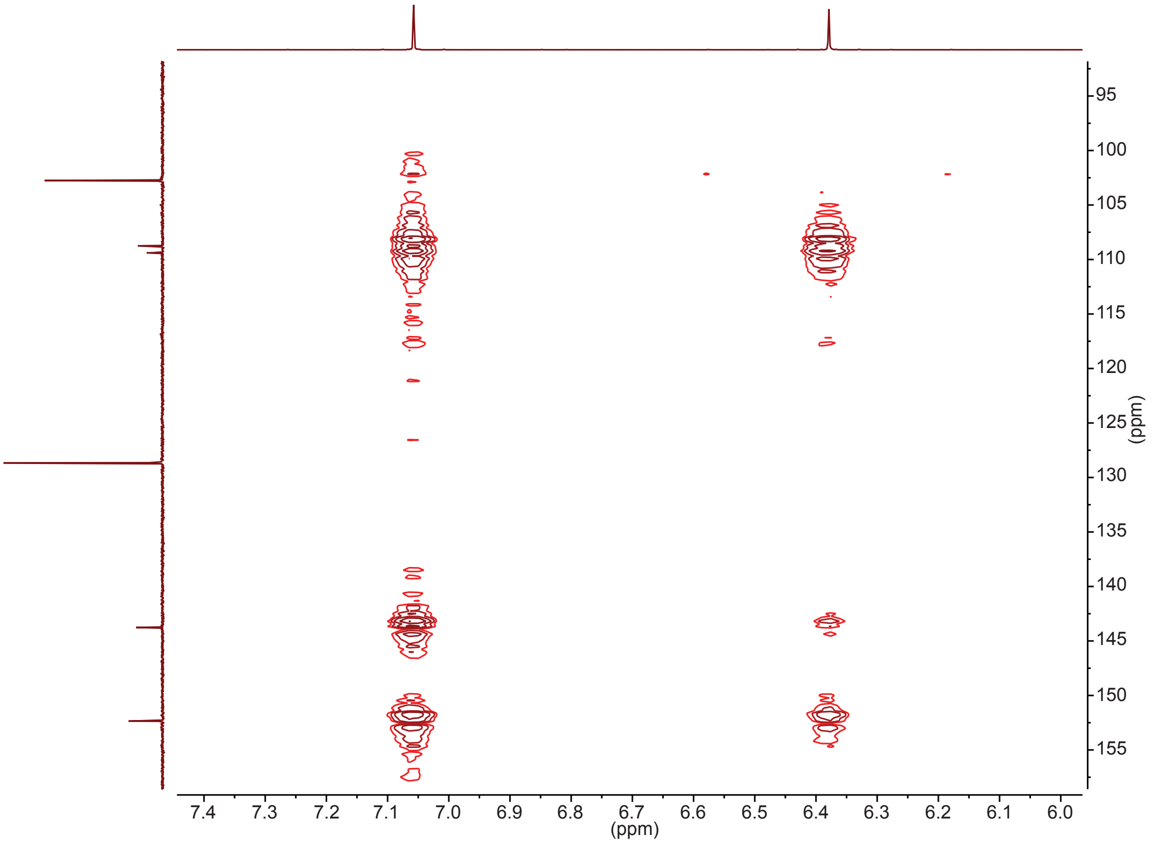

Supplementary Fig. 56. ^{1}H NMR spectrum for compound 2,4-dichloro-1,5-diaminobenzene (2,4-diCl-**18**) in CD_3OD (500 MHz).

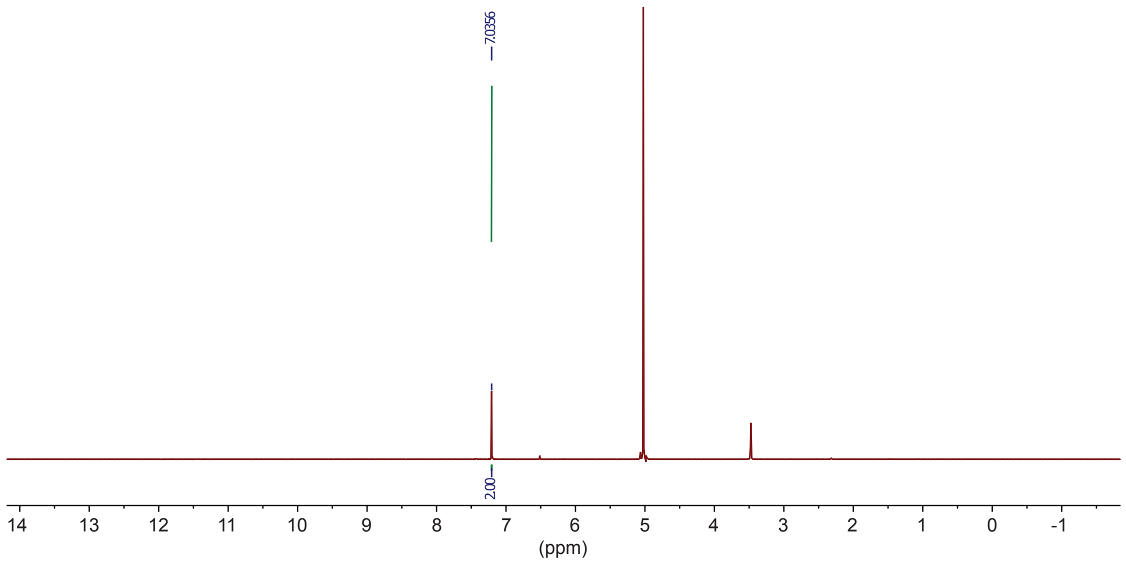

Supplementary Fig. 57. ^{13}C NMR spectrum for compound 2,4-dichloro-1,5-diaminobenzene (2,4-diCl-**18**) in CD_3OD (100 MHz).

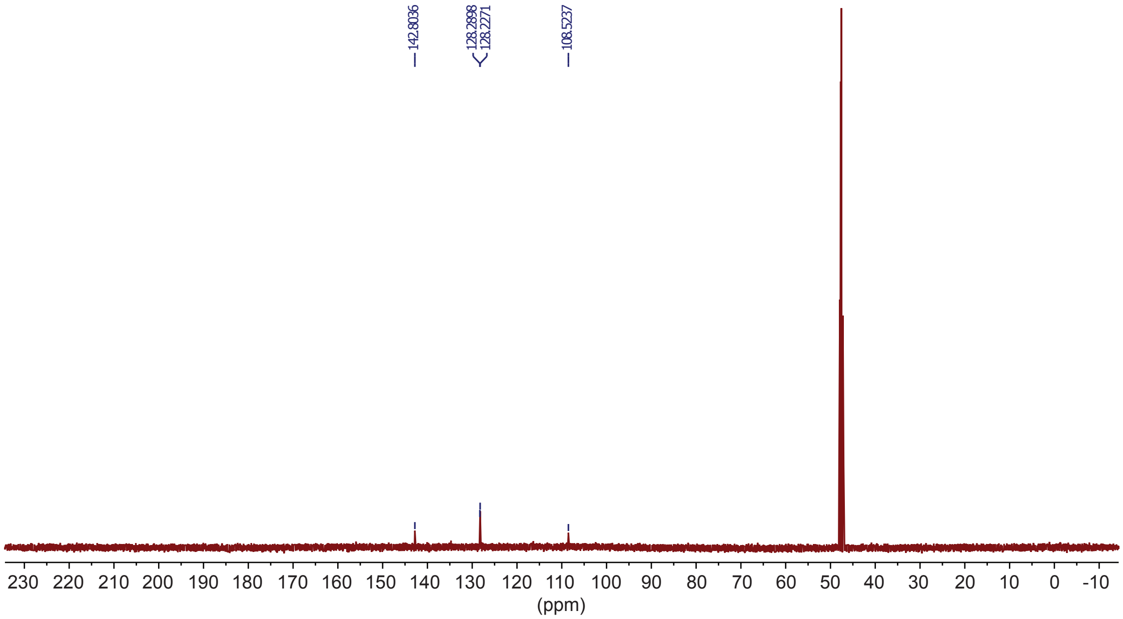

Supplementary Fig. 58. ^{1}H NMR spectrum for compound 4-chloro-resveratrol (4-Cl-**23**) in CD_3OD (400 MHz).

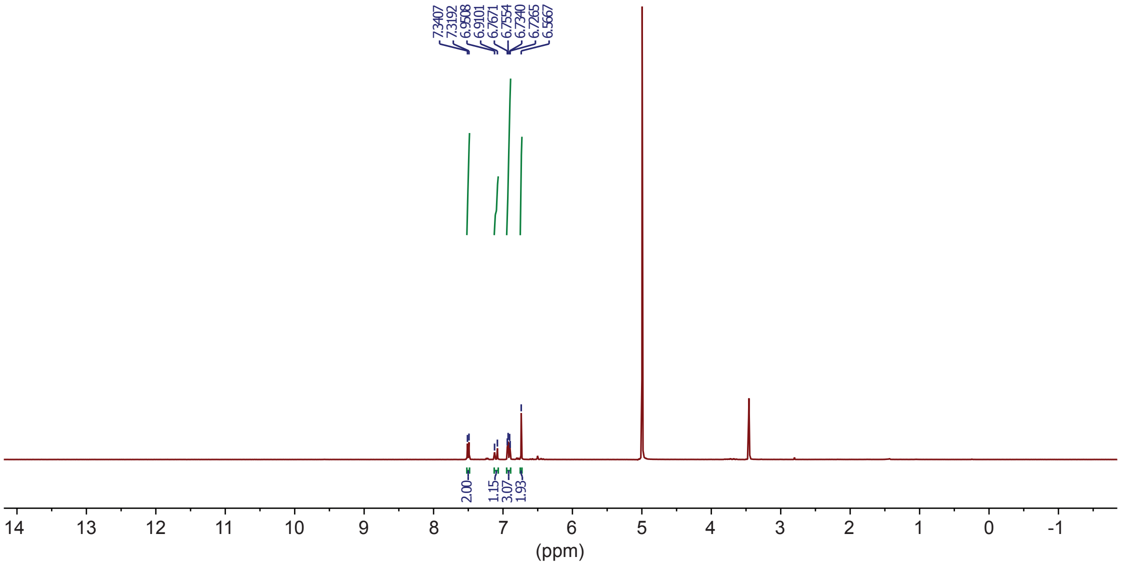

Supplementary Fig. 59. ^1H NMR spectrum for compound resveratrol (**23**) in CD_3OD (400 MHz). Commercially available resveratrol used for comparison with **Supplementary Fig. 58** to determine the position of Cl.

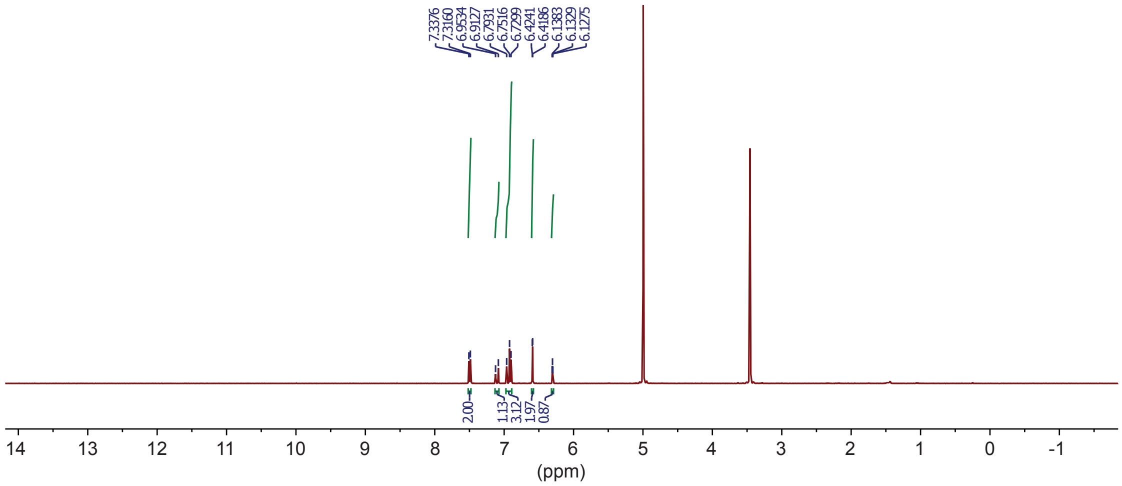

Supplementary references

1. Zhu, X.; De Laurentis, W.; Leang, K.; Herrmann, J.; Ihlefeld, K.; van Pee, K. H.; Naismith, J. H., Structural insights into regioselectivity in the enzymatic chlorination of tryptophan. *J. Mol. Biol.* **2009,** *391* (1), 74-85.
2. Ortega, M. A.; Cogan, D. P.; Mukherjee, S.; Garg, N.; Li, B.; Thibodeaux, G. N.; Maffioli, S. I.; Donadio, S.; Sosio, M.; Escano, J.; Smith, L.; Nair, S. K.; van der Donk, W. A., Two flavoenzymes catalyze the post-translational generation of 5-chlorotryptophan and 2-aminovinyl-cysteine during NAI-107 biosynthesis. *ACS Chem. Biol.* **2017,** *12* (2), 548-557.
3. Shepherd, S. A.; Menon, B. R.; Fisk, H.; Struck, A. W.; Levy, C.; Leys, D.; Micklefield, J., A structure-guided switch in the regioselectivity of a tryptophan halogenase. *ChemBioChem* **2016,** *17* (9), 821-824.
4. Dong, C.; Flecks, S.; Unversucht, S.; Haupt, C.; van Pee, K. H.; Naismith, J. H., Tryptophan 7-halogenase (PrnA) structure suggests a mechanism for regioselective chlorination. *Science* **2005,** *309* (5744), 2216-2219.
5. Flecks, S.; Patallo, E. P.; Zhu, X.; Ernyei, A. J.; Seifert, G.; Schneider, A.; Dong, C.; Naismith, J. H.; van Pee, K. H., New insights into the mechanism of enzymatic chlorination of tryptophan. *Angew. Chem.* **2008,** *47* (49), 9533-9536.
6. Shepherd, S. A.; Karthikeyan, C.; Latham, J.; Struck, A. W.; Thompson, M. L.; Menon, B. R.; Styles, M. Q.; Levy, C.; Leys, D.; Micklefield, J., Extending the biocatalytic scope of regiocomplementary flavin-dependent halogenase enzymes. *Chem. Sci.* **2015,** *6*, 3454-3460.
7. Bitto, E.; Huang, Y.; Bingman, C. A.; Singh, S.; Thorson, J. S.; Phillips, G. N., Jr., The structure of flavin-dependent tryptophan 7-halogenase RebH. *Proteins* **2008,** *70* (1), 289-293.
8. Yeh, E.; Blasiak, L. C.; Koglin, A.; Drennan, C. L.; Walsh, C. T., Chlorination by a long-lived intermediate in the mechanism of flavin-dependent halogenases. *Biochemistry* **2007,** *46* (5), 1284-1292.
9. Menon, B. R.; Latham, J.; Dunstan, M. S.; Brandenburger, E.; Klemstein, U.; Leys, D.; Karthikeyan, C.; Greaney, M. F.; Shepherd, S. A.; Micklefield, J., Structure and biocatalytic scope of thermophilic flavin-dependent halogenase and flavin reductase enzymes. *Org. Biomol. Chem.* **2016,** *14* (39), 9354-9361.
10. Fraley, A. E.; Garcia-Borras, M.; Tripathi, A.; Khare, D.; Mercado-Marin, E. V.; Tran, H.; Dan, Q.; Webb, G. P.; Watts, K. R.; Crews, P.; Sarpong, R.; Williams, R. M.; Smith, J. L.; Houk, K. N.; Sherman, D. H., Function and structure of MalA/MalA', iterative halogenases for late-stage C-H functionalization of indole alkaloids. *J. Am. Chem. Soc.* **2017,** *139* (34), 12060-12068.
11. Pang, A. H.; Garneau-Tsodikova, S.; Tsodikov, O. V., Crystal structure of halogenase PltA from the pyoluteorin biosynthetic pathway. *J. Struct. Biol.* **2015,** *192* (3), 349-357.
12. El Gamal, A.; Agarwal, V.; Diethelm, S.; Rahman, I.; Schorn, M. A.; Sneed, J. M.; Louie, G. V.; Whalen, K. E.; Mincer, T. J.; Noel, J. P.; Paul, V. J.; Moore, B. S., Biosynthesis of coral settlement cue tetrabromopyrrole in marine bacteria by a uniquely adapted brominase-thioesterase enzyme pair. *Proc. Natl. Acad. Sci., U. S. A.* **2016,** *113* (14), 3797-3802.
13. Buedenbender, S.; Rachid, S.; Muller, R.; Schulz, G. E., Structure and action of the myxobacterial chondrochloren halogenase CndH: a new variant of FAD-dependent halogenases. *J. Mol. Biol.* **2009,** *385* (2), 520-530.
14. Podzelinska, K.; Latimer, R.; Bhattacharya, A.; Vining, L. C.; Zechel, D. L.; Jia, Z., Chloramphenicol biosynthesis: the structure of CmlS, a flavin-dependent halogenase showing a covalent flavin-aspartate bond. *J. Mol. Biol.* **2010,** *397* (1), 316-331.

15. Lovell, S. C.; Davis, I. W.; Arendall, W. B., 3rd; de Bakker, P. I.; Word, J. M.; Prisant, M. G.; Richardson, J. S.; Richardson, D. C., Structure validation by Calpha geometry: phi,psi and Cbeta deviation. *Proteins* **2003,** *50* (3), 437-450.
16. Krissinel, E.; Henrick, K., Secondary-structure matching (SSM), a new tool for fast protein structure alignment in three dimensions. *Acta Crystallogr. D Biol Crystallogr.* **2004,** *60* (Pt 12 Pt 1), 2256-2268.
17. Robert, X.; Gouet, P., Deciphering key features in protein structures with the new ENDscript server. *Nucl. Acids Res.* **2014,** *42* (Web Server issue), W320-324.
18. Kuzmic, P., Program DYNAFIT for the analysis of enzyme kinetic data: application to HIV proteinase. *Anal. Biochem.* **1996,** *237* (2), 260-273.
