# Supplementary material for: Unusual substrate and halide versatility of phenolic halogenase PltM
Source: Nat Commun. 2019 Mar 19;10:1255. doi: 10.1038/s41467-019-09215-9 (PMC6424973; doi:10.1038/s41467-019-09215-9)
Supplement: Supplementary file 2 — Reporting Summary [file 41467_2019_9215_MOESM2_ESM.pdf]

nature research

Corresponding author(s): Oleg V. Tsodikov and Sylvie Garneau-Tsodikova

Life Sciences Reporting Summary

Nature Research wishes to improve the reproducibility of the work that we publish. This form is intended for publication with all accepted life science papers and provides structure for consistency and transparency in reporting. Every life science submission will use this form; some list items might not apply to an individual manuscript, but all fields must be completed for clarity.

For further information on the points included in this form, see Reporting Life Sciences Research. For further information on Nature Research policies, including our data availability policy, see Authors & Referees and the Editorial Policy Checklist.

Please do not complete any field with "not applicable" or n/a. Refer to the help text for what text to use if an item is not relevant to your study. For final submission: please carefully check your responses for accuracy; you will not be able to make changes later.

Experimental design

1. Sample size

Describe how sample size was determined.

> There is no statistical analysis in this manuscript.

2. Data exclusions

Describe any data exclusions.

> No data was excluded for this manuscript.

3. Replication

Describe the measures taken to verify the reproducibility of the experimental findings.

> Structural data were highly redundant. X-ray diffraction was highly reproducible from one crystal to another of the same type.

4. Randomization

Describe how samples/organisms/participants were allocated into experimental groups.

> Not applicable.

5. Blinding

Describe whether the investigators were blinded to group allocation during data collection and/or analysis.

> Not applicable.

Note: all in vivo studies must report how sample size was determined and whether blinding and randomization were used.

6. Statistical parameters

For all figures and tables that use statistical methods, confirm that the following items are present in relevant figure legends (or in the Methods section if additional space is needed).

n/a	Confirmed	
☒	☐	The exact sample size (*n*) for each experimental group/condition, given as a discrete number and unit of measurement (animals, litters, cultures, etc.)
☒	☐	A description of how samples were collected, noting whether measurements were taken from distinct samples or whether the same sample was measured repeatedly
☒	☐	A statement indicating how many times each experiment was replicated
☒	☐	The statistical test(s) used and whether they are one- or two-sided<br>*Only common tests should be described solely by name; describe more complex techniques in the Methods section.*
☒	☐	A description of any assumptions or corrections, such as an adjustment for multiple comparisons
☒	☐	Test values indicating whether an effect is present<br>*Provide confidence intervals or give results of significance tests (e.g. P values) as exact values whenever appropriate and with effect sizes noted.*
☒	☐	A clear description of statistics including central tendency (e.g. median, mean) and variation (e.g. standard deviation, interquartile range)
☒	☐	Clearly defined error bars in all relevant figure captions (with explicit mention of central tendency and variation)

See the web collection on statistics for biologists for further resources and guidance.

Software

Policy information about availability of computer code

7. Software

Describe the software used to analyze the data in this study.	PeakView was used to analyze and extract the LC/MS data. Adobe Illustrator CC 2017 was used to make figures. ChemDraw Prime 16.0 was used to draw chemical structures. HKL-2000 was used to process X-ray diffractions data. ShelXD followed by Autosolve in Phenix package were used to determine the crystal structure of the mercury derivative crystal of PltM. Phaser was used to determine the crystal structures of other crystal forms. Refmac and Coot were used to refine and build the crystal structure, respectively. Phenix.polder was used to generate the polder omit electron density map. Pymol was used to make figures of the crystal structure. DynaFit was used for the analysis of progress curves.

For manuscripts utilizing custom algorithms or software that are central to the paper but not yet described in the published literature, software must be made available to editors and reviewers upon request. We strongly encourage code deposition in a community repository (e.g. GitHub). *Nature Methods* guidance for providing algorithms and software for publication provides further information on this topic.

Materials and reagents

Policy information about availability of materials

8. Materials availability

Indicate whether there are restrictions on availability of unique materials or if these materials are only available for distribution by a third party.	Expression vectors will be available upon request from the authors upon manuscript publication, conditional upon proper material transfer documentation (as governed by the University of Kentucky and applicable state and federal regulations). The crystal structures and structure factor amplitudes were deposited in the Protein Data Bank under accession numbers 6BZN, 6BZI, 6BZA, 6BZQ, 6BZT, and 6BZZ. They will be available to general public upon manuscript publication.

9. Antibodies

Describe the antibodies used and how they were validated for use in the system under study (i.e. assay and species).	Not applicable.

10. Eukaryotic cell lines

a. State the source of each eukaryotic cell line used.	Not applicable.
b. Describe the method of cell line authentication used.	Not applicable.
c. Report whether the cell lines were tested for mycoplasma contamination.	Not applicable.
d. If any of the cell lines used are listed in the database of commonly misidentified cell lines maintained by ICLAC, provide a scientific rationale for their use.	Not applicable.

Animals and human research participants

Policy information about studies involving animals; when reporting animal research, follow the ARRIVE guidelines

11. Description of research animals

Provide all relevant details on animals and/or animal-derived materials used in the study.	Not applicable.

Policy information about studies involving human research participants

12. Description of human research participants

Describe the covariate-relevant population characteristics of the human research participants.	Not applicable.
